# A Serum‐Stable Antimicrobial Peptide‐Based Delivery Platform for Selective Treatment of Nontargetable and Chemoresistant Tumors

**DOI:** 10.1002/advs.202504710

**Published:** 2025-09-17

**Authors:** Tianxing Liu, Lingyu Ke, Ruize Sun, Xiaoling Chen, Mei Zhou, Olaf R. P. Bininda‐Emond, Chengbang Ma, Yangyang Jiang, Tao Wang, Chris Shaw, Lei Wang, Tianbao Chen, Alideertu Dong

**Affiliations:** ^1^ Natural Drug Discovery Group School of Pharmacy Queen's University Belfast Belfast Northern Ireland BT71NN UK; ^2^ AG Systematik und Evolutionsbiologie IBU‐Faculty V Carl von Ossietzky University Oldenburg 26111 Oldenburg Germany; ^3^ College of Chemistry and Chemical Engineering Engineering Research Center of Dairy Quality and Safety Control Technology Ministry of Education Inner Mongolia University 235 Daxue West Road Hohhot 010021 P. R. China

**Keywords:** activatable linkers, antimicrobial peptides, chemotherapy resistance, membrane affinity reconstruction, serum stability, therapeutic window

## Abstract

Antibody–drug conjugates (ADCs) have transformed cancer therapy but remain limited by their dependence on internalizing antigens, poor applicability to untargetable tumors, and susceptibility to drug resistance. Therefore, a modular antimicrobial‐peptide (AMP)‐based therapeutic system centered on a rationally designed conjugate, 270, is presented, which integrates three optimized components: a selectivity‐enhanced AMP core via a membrane affinity reconstruction strategy, a conformation‐driven polyethylene glycolylated blocker to minimize off‐target effects, and an N‐terminal cap to improve stability in human serum. By targeting nonendocytic membrane surface receptors via small‐molecule ligands, conjugate 270 exhibits potent and selective cytotoxicity against target tumor cells, effectively eliminating the majority of tumor cells within a few hours. Meanwhile, it exhibits high serum stability, minimal hemolysis, and negligible cytotoxicity toward normal cells at therapeutic concentrations. Mechanistic studies confirm ligand‐dependent membrane localization, rapid depolarization, and disruption, along with mitochondrial dysfunction. Moreover, it demonstrates significant therapeutic efficacy against four cell lines resistant to conventional chemotherapeutic agents. While additional in vivo validation is warranted, this work lays the foundation for a flexible AMP‐based approach to address untargetable and drug‐resistant cancers.

## Graphical abstract

A modular antimicrobial‐peptide‐based therapeutic system integrating membrane‐targeted selectivity, a conformation‐driven polyethylene glycolylated blocker, and serum‐stabilizing N‐cap is developed. The optimized conjugate 270 shows potent, rapid killing of the desired tumor cells, high serum stability, low off‐target toxicity, and broad applicability, offering a promising strategy for nontargetable and chemoresistant cancers.

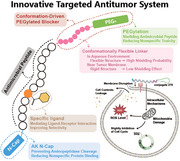

## 1 Introduction

The introduction of antibody–drug conjugates (ADCs) marks a significant milestone in targeted cancer therapy, offering highly selective cytotoxic effects against tumor cells while minimizing toxicity to healthy tissues.^[^ ^1^, ^2^ ^]^ Since the approval of the first ADC in 2000,^[^ ^3^ ^]^ a total of 14 ADCs have received market authorization from the U.S. Food and Drug Administration.^[^ ^4^ ^]^ Despite this success, ADCs face notable limitations. Their effectiveness depends on specific internalizing antigen targets, yet only ten proteins have been identified as ADC targets, with four in solid tumors and six in hematologic malignancies.^[^ ^4^ ^]^ Tumors expressing these proteins represent a small subset of all cancers.^[^ ^5^, ^6^ ^]^ This restricts ADC applicability, leaving many patients with tumors lacking these targets unable to benefit from therapy. Moreover, ADC payloads act intracellularly, making them vulnerable to drug resistance mechanisms such as efflux pumps and intracellular metabolism, significantly reducing therapeutic efficacy.^[^ ^7^, ^8^ ^]^ Overcoming these limitations requires novel therapeutic strategies that bypass reliance on receptor‐mediated endocytosis and intracellular delivery. Antimicrobial peptides (AMPs), known for their unique membrane‐disruptive mechanism,^[^ ^9^, ^10^, ^11^ ^]^ appear to align with these requirements. Recent studies have demonstrated promising anticancer applications of AMPs through rational modifications. For example, substitution of l‐amino acids with d‐amino acids in melittin analogs markedly reduced immunogenicity and proteolytic degradation while maintaining cytotoxic activity.^[^ ^12^ ^]^ Lipidation of amphipathic peptides such as CAMEL enhanced membrane interaction and anticancer efficacy in a chain‐length‐dependent manner.^[^ ^13^ ^]^ Tumor‐homing motifs like RGD have been introduced into α‐helical AMPs to increase binding selectivity toward integrin αvβ3‐expressing tumor cells.^[^ ^14^ ^]^ Backbone cyclization strategy has been used to stabilize secondary structure and enhance bioactivity under physiological conditions.^[^ ^15^ ^]^ In addition, polyethylene glycolylation (PEGylation) at the terminus has been shown to reduce hemolysis, improve serum half‐life, and decrease systemic toxicity without abolishing antitumor function.^[^ ^16^ ^]^ Together, these approaches highlight the adaptability of AMPs for cancer therapy. Despite these advances, current strategies often rely on case‐specific chemical modifications that are not readily generalizable across AMP families. Many engineered peptides still exhibit toxicity to normal cells or fail to retain their activity under systemic conditions. In particular, efforts to simultaneously improve tumor selectivity, protease resistance, and pharmacokinetics have yielded only limited success. Furthermore, a systematic framework for integrating multiple optimizations into a modular, general‐purpose AMP platform is still lacking. These challenges underscore the lack of generalizable frameworks and highlight the need for modular solutions to advance AMP‐based therapeutics toward clinical translation.

This study aimed to develop a series of first‐in‐class universal strategies and methodologies to establish an innovative targeted therapy system utilizing AMPs as payloads for treating conventionally untargetable tumors and chemotherapeutic‐resistant cancers. The H460‐cell‐line‐representing large cell lung carcinoma (LCLC), a subtype of non‐small cell lung cancer (NSCLC) was selected as the target model. This cell line lacks the specific receptors necessary for conventional targeted therapies,^[^ ^17^, ^18^ ^]^ highlighting it as a quintessential example of an untargetable tumor for which effective treatment options remain unavailable. The system would be constructed stepwise, starting with a representative AMP and culminating in the development of the final therapeutic compound. This approach demonstrated both the establishment and optimization of the system, as well as the feasibility of the proposed strategies.

## 2 Results and Discussions

### 2.1 Model AMP Selection and Initial Biological Evaluation

Temporin‐HLa (compound 111), discovered by our group in 2021,^[^ ^19^ ^]^ was selected as a model AMP due to its typical AMP properties, making it an appropriate candidate for investigating strategies to address the challenges of selectivity and stability in AMP‐based cancer therapeutics. Compound 111 exhibited broad‐spectrum antiproliferative activities and significant membrane disruption capabilities against various solid tumor cells at micromolar concentrations (Table S1 and Figure S37A,D, Supporting Information). Its anticancer effects were impacted by serum, as evidenced by a maximum 2.7‐fold increase in IC_50_ values following 4 h of coincubation with 50% human serum (Figure S37B,C, Supporting Information). It also showed significant cytotoxicity against all tested normal cell lines and erythrocytes (Table S1, Supporting Information), revealing its poor selectivity and a narrow therapeutic window (TW).

### 2.2 Selectivity Improvement Using “Membrane Affinity Reconstruction” Strategy

#### 2.2.1 Strategy Development and Compound Design

We hypothesized that the poor selectivity of AMPs is associated with their binding mechanism to cell membranes, specifically via electrostatic or hydrophobic interactions,^[^ ^10^ ^]^ and these interactions are insufficient to differentiate between tumor and normal cell membranes. To overcome this, the “membrane affinity reconstitution” strategy was developed, aiming to impart AMPs with specific membrane binding affinity through receptor–ligand interactions. Initially, certain amino acids would be substituted to decrease their net positive charge and hydrophobicity, thereby reducing the membrane binding affinity and disruption ability toward all cells. Subsequently, a small molecule group, capable of specifically binding to proteins overexpressed on the surface of tumor cell membranes, would be conjugated to the modified AMP at an appropriate position. The amphipathic helical structure of the peptide should be maintained throughout these processes to preserve its membrane disruption ability on target cells. In this way, the final conjugate was expected to exhibit significantly higher binding affinity and membrane disruption capability toward target tumor cells while being less toxic to normal cells.

**Figure 1:**
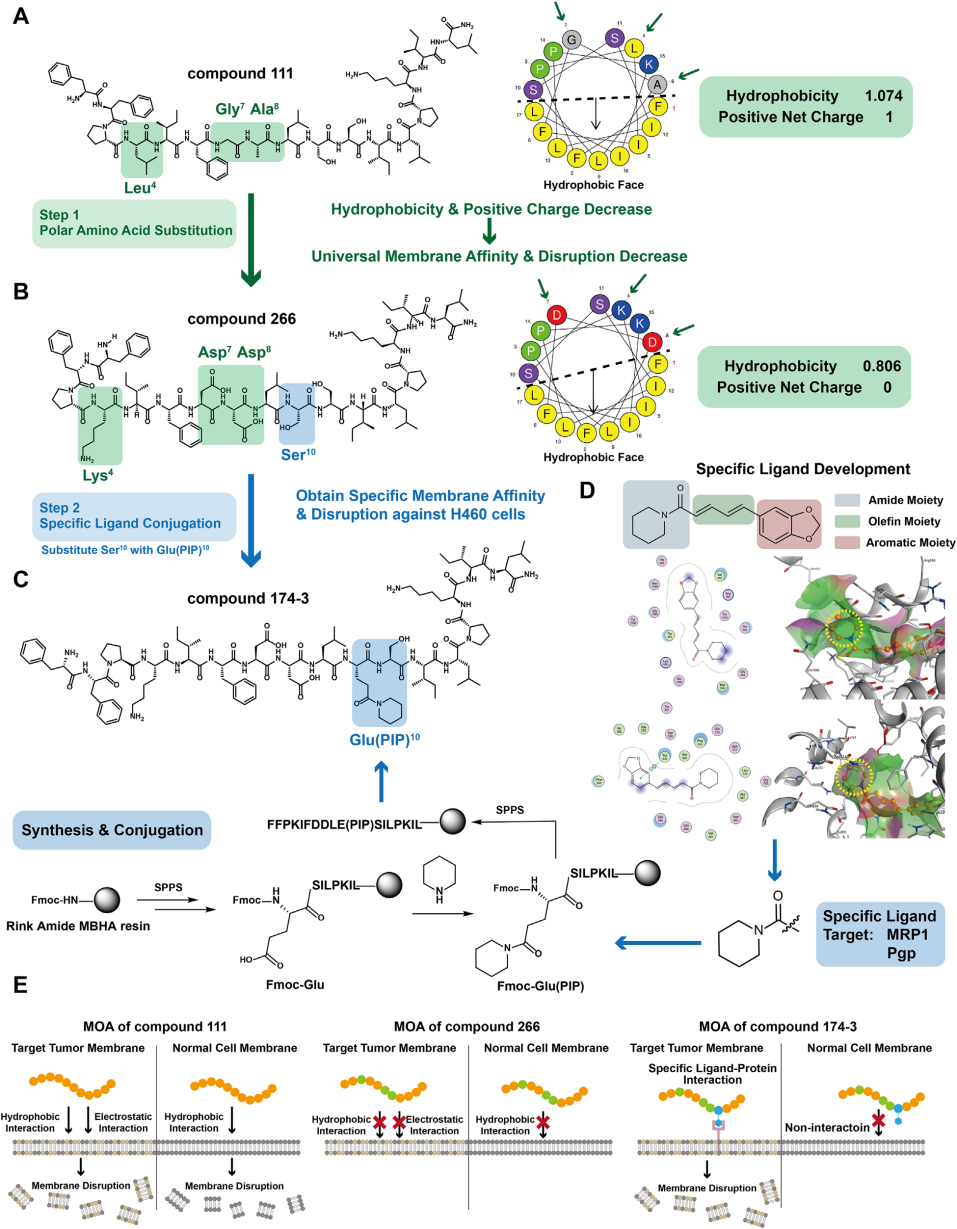
Schematic overview of the stepwise “membrane affinity reconstruction” strategy. A,B) The chemical structures, helical wheel diagrams, physicochemical properties (predicted using HeliQuest), and substituted amino acids of compounds 111 and 266 are presented. C) The chemical structure and synthetic route of compound 174‐3 are shown. D) The specific ligand development process is displayed. The chemical structure of piperine contains three moieties. Predicted docking 2D interaction patterns (left) and 3D cartoon interaction patterns (right) between piperine and MRP1 (upper) and Pgp (lower) are presented. In 3D cartoon interaction, receptors are gray, ligands are orange, and blue dashed lines represent hydrogen bonds. The interpretation of the legend in the 2D interaction patterns is provided in Figure S38A (Supporting Information). The piperidine ring is highlighted with a yellow circle. The ligand chemical structure is presented at the bottom of (D). E) The proposed modes of action (MOA) of three compounds are summarized.

Based on the above strategy, two compounds were generated. First, guided by an analysis of the overall amphipathic structure of compound 111, three residues on the nonhydrophobic face, Leu⁴, Gly⁷, and Ala⁸, were substituted with Lys⁴, Asp⁷, and Asp⁸, respectively, to yield compound 266. This modification aimed to reduce the original membrane affinity and disruptive potential toward all cell types by decreasing both hydrophobicity and net positive charge (**Figure** **1**A,B,E). Next, to endow compound 266 with selective binding affinity for the H460 cell line, a tumor‐targeting small‐molecule ligand was sought for conjugation (Figure 1C). Literature analysis of H460 surface proteins identified multidrug‐resistance‐associated protein 1 (MRP1) and P‐glycoprotein (Pgp) as promising targets due to their overexpression.^[^ ^20^, ^21^ ^]^ Inspired by piperine, a known small‐molecule inhibitor of both MRP1 and Pgp,^[^ ^22^ ^]^ molecular docking studies showed that its amide and aromatic moieties interacted with distinct binding pockets on the proteins, while the olefin moiety served as a linker. These findings suggested that both the amide and aromatic groups contribute to receptor binding. The amide moiety was selected as the ligand anchor due to its key role in targeted protein binding and synthetic accessibility, allowing site‐specific conjugation to the peptide scaffold. Specifically, the amino group of piperidine was conjugated to the side‐chain carboxyl group of a natural glutamic acid residue, generating an unnatural amino acid, Glu(piperidine) Glu(PIP), in which the side chain was structurally redesigned to mimic the amide functionality of piperine. Ser^10^ in compound 266 was then replaced with Glu(PIP)^10^, resulting in compound 174‐3. This final construct preserved the peptide backbone of 266 while incorporating a specific small‐molecule ligand, thereby minimizing off‐target membrane disruption and enhancing interaction with target tumor cell membranes through ligand‐directed targeting of MRP1/Pgp (Figure 1D,E).

#### 2.2.2 Compound 174‐3 Exhibits Enhanced Selective Cytotoxicity

**Figure 2:**
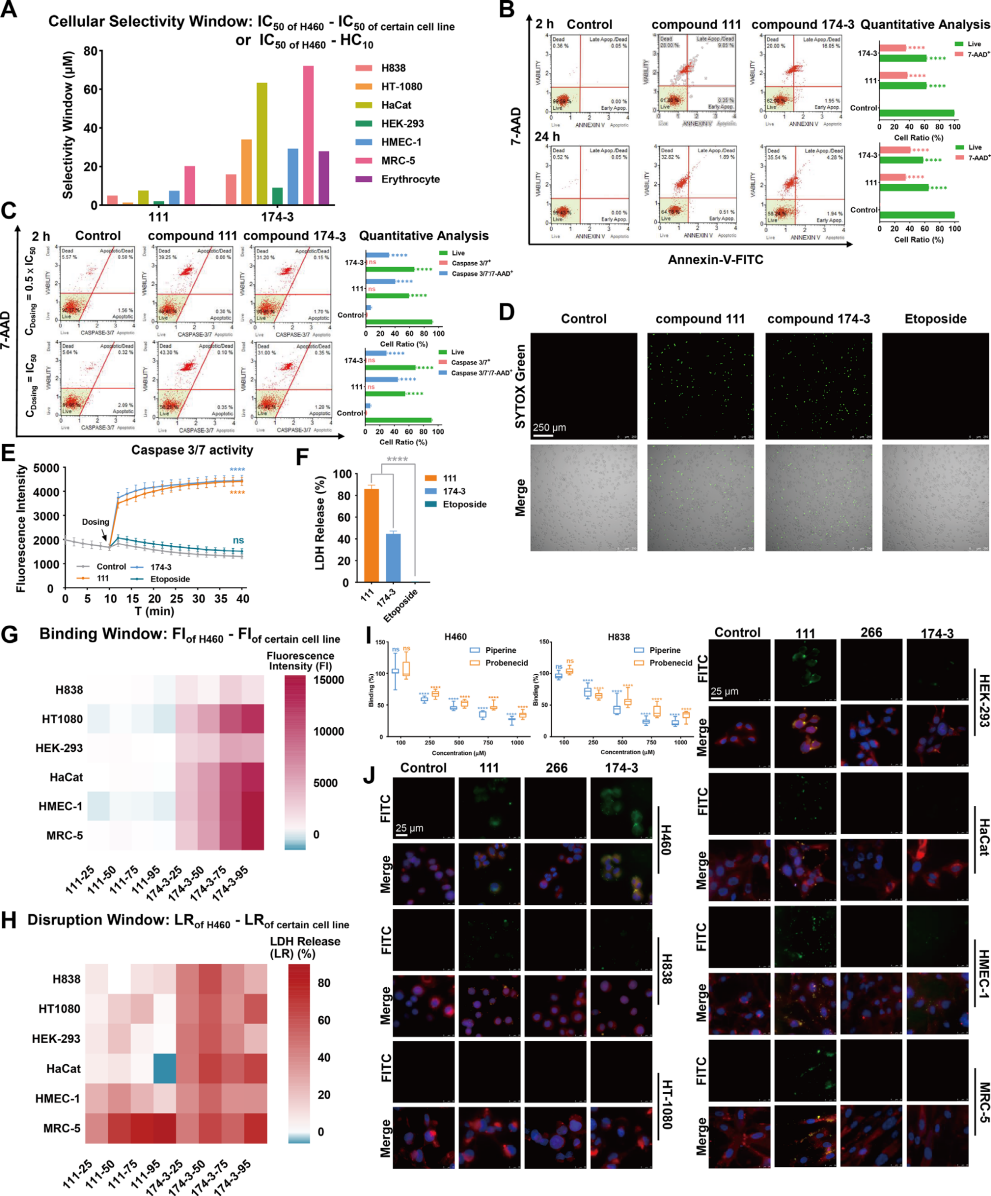
A) Cellular selectivity window of compounds 111 or 174‐3 against different cell lines. B) Annexin V/7‐aminoactinomycin D (7‐AAD) flow cytometry analysis of H460 cells treated with compounds 111 or 174‐3. C) Caspase‐3/7 and 7‐AAD flow cytometry analysis of H460 cells treated with compounds 111 or 174‐3. D) Fluorescence images of H460 cells treated with compounds 111, 174‐3, or etoposide at each IC_50_ value for 2 h. Green channel (SYTOX Green) was merged with bright fields (scale bar: 250 µm). E) Membrane potential of H460 cells treated with compounds 111, 174‐3, or etoposide at each IC_50_ value, evaluated by fluorescence intensity. F) Lactatedehydrogenase (LDH) release (%) of H460 cells after 4 h treatment with compounds 111, 174‐3, or etoposide at each IC_50_ value. G) Binding window and H) disruption window of compounds 111 and 174‐3 against different cell lines. I) Cell binding (%) of compound 174‐3 to H460 or H838 cell lines in the presence of MRP1/Pgp inhibitor, piperine, or MRP1 inhibitor, probenecid. J) Visualization of cell binding assay. Different cells were treated with fluorescein isothiocyanate isomer I (FITC)‐labeled peptides for 2 h and stained with 4′,6‐diamidino‐2′‐phenylindole (DAPI) and FM4‐64. Green channel (FITC) was merged with blue channel (DAPI) and red channel (FM4‐64) (scale bar: 25 µm). Data represent the mean ± standard error of the mean (SEM), *n* = 3. One‐way (ANOVA) followed by Dunnett's post hoc test was used to compare each treatment group with the control group in (B), (C), (E), and (I) (control group in (I) is not shown), or with etoposide group (F). ns for *p* > 0.05 and **** for *p* < 0.0001.

To assess whether the “membrane affinity reconstruction” strategy could achieve its intended purpose, we performed biological evaluations and mechanistic investigations. The H838 cell line, characterized by moderate MRP1 expression and absence of Pgp (confirmed at the mRNA level),^[^ ^20^ ^]^ was selected as a model for intermediate MRP1 expression. HT‐1080 and four normal cell lines, HEK‐293, MRC‐5, HMEC‐1, and HaCaT, lacking MRP1 and Pgp overexpression,^[^ ^23^, ^24^, ^25^, ^26^ ^]^ were included to evaluate potential off‐target effects. In total, seven tumor and normal cell lines with varying receptor expression profiles were used to assess the selectivity and efficacy of the designed compounds. To complement the cellular assays, hemolysis evaluation was incorporated as an additional measure of off‐target toxicity. We examined the biological activity of compounds 111, 266, and 174‐3 as a direct way to assess the effectiveness of the strategy (Table S1, Supporting Information). Compound 266 exhibited neither hemolytic activity nor antiproliferative effects against any of the tested cell lines at the evaluated concentrations. By contrast, compound 174‐3 regained cytotoxic activity across all cell lines. Notably, it displayed the greatest potency against the H460 cell line (IC_50_: 23.65 µm), followed by moderate activity against the H838 cell line (IC_50_: 39.54 µm), while demonstrating only weak effects on other cell lines and erythrocytes (IC_50_ or hemolysis concentration causing 10% lysis (HC_10_): 50–100 µm). These results suggest that the cytotoxic activity of compound 174‐3 correlates positively with receptor expression levels. Compound 111 showed similar cytotoxicity across all tested cell lines, with no significant preference for cancer cells. This was further supported by the selectivity window illustrated in **Figure** **2**A. These results provide preliminary evidence that the “membrane affinity reconstruction” strategy successfully enhanced the selectivity of peptide activity. To further determine whether the observed selectivity arose from the intended mechanism, we next performed detailed mechanism studies.

#### 2.2.3 Membrane Disruption Underlies the Antitumor Activity of Compound 174‐3

To investigate the antitumor mechanism of action of compounds 111 and 174‐3, we began by assessing the cell death pathway using annexin V/7‐AAD staining. H460 cells treated at their respective IC_50_ concentrations for 2 or 24 h predominantly exhibited a 7‐AAD⁺/annexin V^−^ population, indicating nonapoptotic death, probably necrosis caused by membrane disruption (Figure 2B). Notably, annexin V fluorescence was higher at 2 h than at 24 h within the 7‐AAD⁺ population, suggesting the possibility of transient early apoptotic signals. To further clarify this observation, we repeated the experiment using half of the IC_50_ dose, and no annexin V⁺/7‐AAD^−^ population was observed (Figure S38B, Supporting Information). In parallel, caspase‐3/7 and 7‐AAD staining revealed only 7‐AAD⁺ cells without caspase activation at both full and half IC_50_ doses, confirming the classical apoptosis pathway was not involved (Figure 2C). Subsequently, 3,3′‐dipropylthiadicarbocyanine iodide (DiSC_3_(5)), SYTOX Green, and LDH assays were used to detect whether two compounds disrupted the H460 cell membrane (Figure 2D–F). DiSC_3_(5) fluorescence increased rapidly after dosing at IC_50_, indicating membrane depolarization. SYTOX Green staining presented nuclear fluorescence in treated cells after 2 h, confirming compromised membrane integrity. LDH release ratio exceeded 50% after 4 h dosing, demonstrating substantial membrane damage. Etoposide, used as an apoptotic control, yielded no positive results in any assay, reinforcing that the observed effects stemmed from AMP‐induced disruption rather than downstream apoptotic events. Collectively, these results confirmed that both compounds 111 and 174‐3 induced rapid, potent, membrane‐disruption‐driven cytotoxicity in H460 cells. The membrane affinity reconstruction strategy preserved this fundamental mechanism during compound modification.

#### 2.2.4 Specific Ligand Drives the Selective Membrane Affinity and Disruption Ability of Compound 174‐3

We next validated whether the selectivity of compound 174‐3 was conferred by the introduced ligand, enabling receptor‐mediated membrane affinity and disruption. To assess this, FITC‐labeled analogs of compounds 111, 266, and 174‐3 were synthesized and used to evaluate membrane‐binding profiles across multiple cell lines (Figure S38C, Supporting Information). Given the significant difference in effective concentrations between compounds 111 and 174‐3, we compared their binding at concentrations corresponding to 25%, 50%, 75%, and 95% inhibition of H460 cell growth. Compound 266 was tested at the same concentration as the 95% potency level of compound 111. Fluorescence quantification showed that compound 111 exhibited uniformly fluorescence intensity across all tested cell lines and potency levels, indicating nonselective binding to cell membranes, while compound 266 showed markedly diminished fluorescence, confirming that the first step of the design strategy effectively reduced nonspecific membrane interactions. By contrast, compound 174‐3 displayed strong, concentration‐dependent binding to H460 (high receptor expression) and H838 (moderate expression), but not to low‐expressing lines—except HEK‐293. We introduced the concept of a “binding window,” an adaptation of the TW, to visualize selectivity differences in membrane affinity across cell types and potency levels (Figure 2G). Visualization images of binding assays further confirmed that compound 174‐3 exhibited significantly greater binding selectivity than compound 111 (Figure 2J). To validate receptor involvement, H460 and H838 cells were coincubated with compound 174‐3 in the presence of increasing concentrations of either the MRP1/Pgp dual inhibitor piperine or the MRP1‐specific inhibitor probenecid. A clear negative correlation between fluorescence intensity and inhibitor concentration confirmed receptor‐mediated, competitive binding (Figure 2I). Based on these findings, we next evaluated membrane disruption ability using LDH assays under the same potency‐aligned design (Figure S38D, Supporting Information). Compound 111 caused equivalent membrane damage across all cell lines, compound 266 showed negligible damage, while compound 174‐3 exhibited the highest degree of membrane damage in H460 cells at all potency levels, demonstrating selective membrane‐disruptive activity. Similarly, the concept of a “disruption window” was also introduced to visualize differences in membrane disruption selectivity between compounds 111 and 174‐3 (Figure 2H). However, at the IC_95_ potency level, 174‐3 also caused increased membrane disruption in HEK‐293 and HMEC‐1, indicating reduced selectivity at higher concentrations. To further validate the origin of membrane selectivity of compound 174‐3, Glu(PIP)^10^ was replaced with the structurally similar Phe^10^ to yield compound 228 (Figure S38E, Supporting Information). Compared to 174‐3, compound 228 lost membrane selectivity at both the functional and mechanistic levels (Figure S38F,G, Supporting Information), confirming that the introduced ligand was the key determinant driving the receptor‐mediated membrane selectivity of compound 174‐3. To initially evaluate the potential immunogenicity of compound 174‐3, TNF‐α secretion in RAW264.7 macrophages was assessed using an enzyme‐linked immunosorbent assay (ELISA) assay. Unlike lipopolysaccharide (LPS), compound 174‐3 did not induce any detectable proinflammatory response, suggesting that it did not elicit acute proinflammatory activation under in vitro tested conditions (Figure S38H, Supporting Information).

Collectively, Section 2.2 demonstrated that the “membrane affinity reconstruction” strategy successfully enhanced the tumor selectivity of AMP‐based therapeutics. By reducing electrostatic and hydrophobic interactions in compound 266 and introducing a receptor‐binding ligand in compound 174‐3, selective membrane affinity and disruption were achieved without altering the original mechanism of action. Compound 174‐3 exhibited strong cytotoxicity toward high MRP1/Pgp‐expressing tumor cells while sparing most normal cells, driven by ligand‐mediated, receptor‐dependent binding. Mechanistic studies confirmed that compound 174‐3 retained membrane disruption as its primary mode of action, and that enhanced selectivity arose specifically from ligand‐mediated, receptor‐dependent interactions, as evidenced by competitive inhibition assays and loss‐of‐function analog design. These findings highlight the ligand as the key determinant in achieving selective membrane targeting and underscore the broader utility potent of this design strategy for AMP functional optimization.

### 2.3 Conformation‐Driven Poly(Ethylene Glycol)‐Modified (PEGylated) Blocker Further Reduces Side Effects

**Figure 3:**
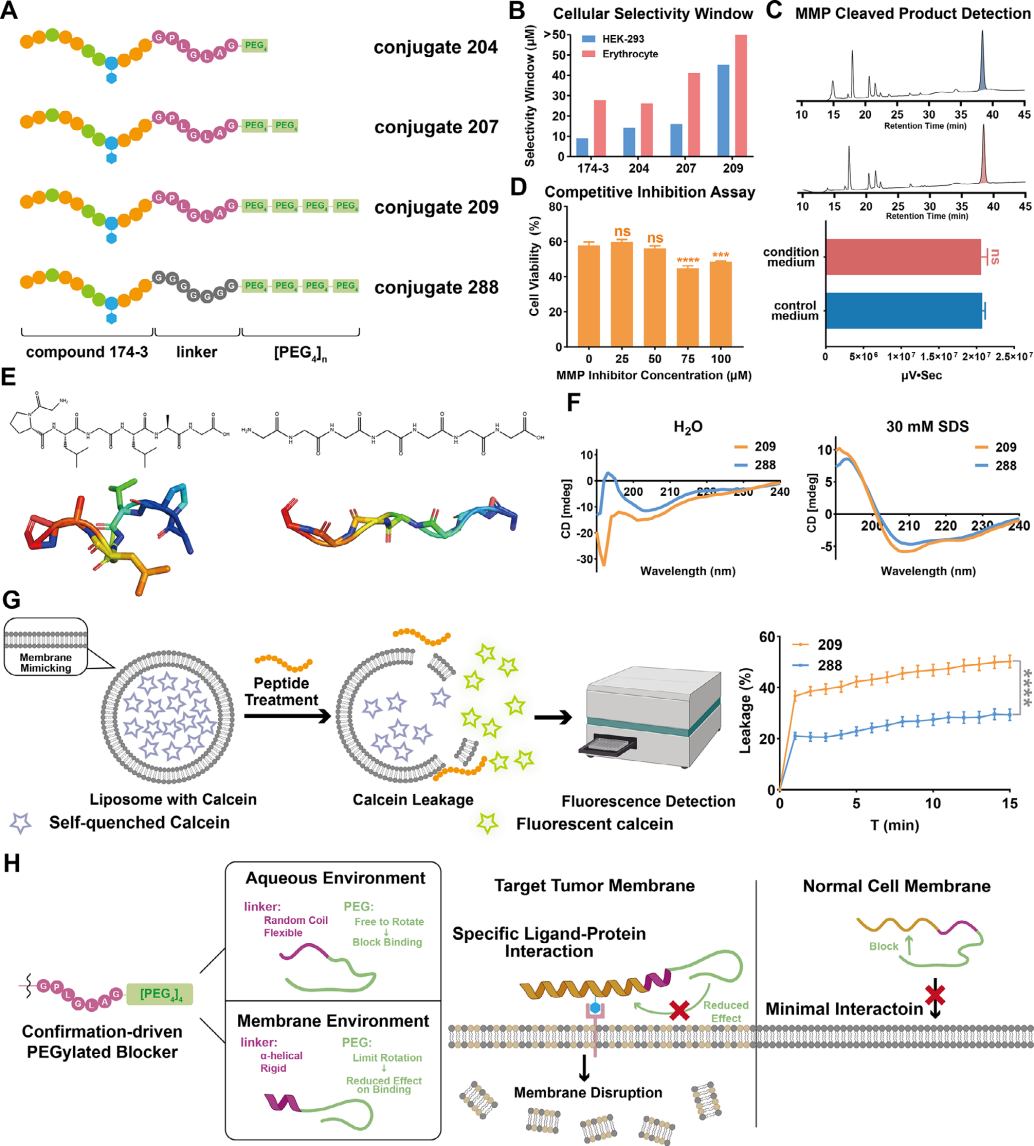
A) Structural components of conjugates 204, 207, 209, and 288. B) Cellular selectivity window of compounds 174‐3 and conjugates 204, 207, and 209 against HEK‐293 cell line and erythrocyte. C) MMP‐cleaved product detection assay using high‐performance liquid chromatography (HPLC) to analyze conjugate 209 sample after incubation with control or condition medium for 10 h. Representative HPLC chromatograms (upper) and peak area (lower) quantification are shown. D) Antiproliferative effect of conjugate 209 at IC_50_ concentration against H460 cells in the presence of different concentrations of MMP inhibitor. E) Chemical structures (upper) and predicted secondary structures (lower) of linker GPLGLAG (left) and GGGGGGG (right). F) Circular dichroism (CD) spectra of conjugates 209 and 288 in ddH_2_O (left) or sodium dodecyl sulfate (SDSsolution (right). G) Brief mechanism scheme of liposome leakage assays (left) and the leakage (%) of calcein‐containing liposomes after treatment with 50 µm of conjugates 209 and 288. H) Scheme illustrating MOA of conformation‐driven PEGylated blocker. Data represent the mean ± SEM, *n* = 2 (C) or *n* = 3 (D and G). One‐way ANOVA followed by Dunnett's post hoc test was used to compare each treatment group with 0 µm of MMP inhibitor dosing group (D) or with conjugate 288 dosing group (G). Unpaired two‐tailed *t*‐test was used to compare the condition medium group with control medium group (C). ns for *p* > 0.05 and **** for *p* < 0.0001.

We observed unintended membrane affinity and disruption by compound 174‐3 toward HEK‐293 cells (IC_50_: 32.63 µm), and its HC_10_ (51.52 µm) demonstrated the significant erythrocyte disruption potential. Since no literature has reported MRP1 or Pgp overexpression on HEK‐293 cells or erythrocytes, these off‐target effects were attributed to the enhanced hydrophobicity conferred by the piperidine moiety. To mitigate this issue, polyethylene glycol (PEG)PEG was conjugated to compound 174‐3 to increase hydrophilicity, using a matrix‐metalloproteinase (MMP)‐cleavable linker,^[^ ^27^ ^]^ GPLGLAG, designed to detach the PEG shield specifically within the tumor microenvironment (Figure S39A, Supporting Information). Three PEGylated conjugates, 204, 207, and 209, were synthesized with varying PEG lengths (**Figure** **3**A). Both PEG and the linker were attached to the C‐terminus to preserve the N‐terminal hydrophobicity, which is essential for maintaining the membrane‐disruptive function characteristic of temporin‐family AMPs.

Biological evaluations revealed that increasing PEG length reduced both hemolytic activity and cytotoxicity toward normal cells, while maintaining antitumor efficacy (Figure 3B; Table S1, Supporting Information). Among the three conjugates, 209 displayed the most favorable selectivity: it retained moderate activity against H460 cells (IC_50_: 53.37 µm), while its IC_50_ against HEK‐293 increased to 98.6 µm and its HC_10_ exceeded 100 µm. For other normal cell lines, the IC_50_ values were all above 100 µm. LDH assays further confirmed that conjugate 209 selectively disrupted H460 membranes at IC_95_, with significantly decreased membrane disruption to normal cells compared to compound 174‐3 (Figure S39B, Supporting Information). These results demonstrated that PEGylation with the linker significantly improved the membrane selectivity of conjugate 209.

To further investigate whether the GPLGLAG linker functioned as designed, we incubated conjugate 209 with condition or control medium for 12 h and analyzed the samples using HPLC and mass spectrometry (MS). No cleaved products were detected and conjugate 209 remained intact. Furthermore, the antitumor activity of conjugate 209 was not decreased in the presence of MMP inhibitors, indicating no enzymatic cleavage within tumor environment (Figure 3C,D). These unexpected findings prompted us to reconsider the function of the linker. We considered that the linker, instead of being cleaved, adopted a rigid conformation near tumor membranes, restricting PEG movement and preserving membrane disruption. In other environments, however, it is likely to remain flexible, enabling PEG to mask the AMP and reduce nonspecific toxicity. We synthesized conjugate 288 as a control by replacing the GPLGLAG linker with a fully flexible GGGGGGG sequence.^[^ ^28^ ^]^ Structural predictions using AlphaFold2 indicated that GPLGLAG adopted an α‐helical conformation, whereas GGGGGGG remained a random coil (Figure 3E). CD spectroscopy confirmed that both conjugates exhibited random coil structures in ddH_2_O but transitioned into α‐helical conformations in 30 mm SDS, which mimicked the membrane environment (Figure 3F). Notably, conjugate 209 displayed a higher α‐helical content than conjugate 288 in SDS, attributed to the differing secondary structures of the linkers. Calcein leakage assay further demonstrated that conjugate 209 induced a significantly greater fluorescence increase than conjugate 288, indicating stronger membrane disruption ability toward tumor‐mimicking membranes (Figure 3G). Finally, antiproliferative assays against H460 and HEK‐293 cell lines demonstrated that conjugate 288, as expected, lost inhibitory activity toward both cell types (Table S1, Supporting Information).

These findings indicated that although initially designed for enzymatic cleavage, the GPLGLAG linker in conjugate 209 functioned through a conformation‐driven mechanism (Figure 3H). Upon membrane environment, it adopted an α‐helical structure that rigidified the PEG moiety and reinstated the membrane disruption function. Crucially, this transformation occurred selectively near tumor membranes, where receptor‐mediated anchoring positioned the AMP in a membrane environment that favored α‐helical transition of the linker. By contrast, the linker remained flexible due to negligible affinity toward nontarget membranes, allowing PEG to maintain steric shielding. This synergy between ligand‐directed membrane affinity and environment‐responsive activation provided a dual mechanism of selectivity, advancing the selectivity of tumor‐targeted AMP conjugates.

### 2.4 N‐Terminal Capping Strategy Improves Serum Stability

#### 2.4.1 Strategy Development

**Figure 4:**
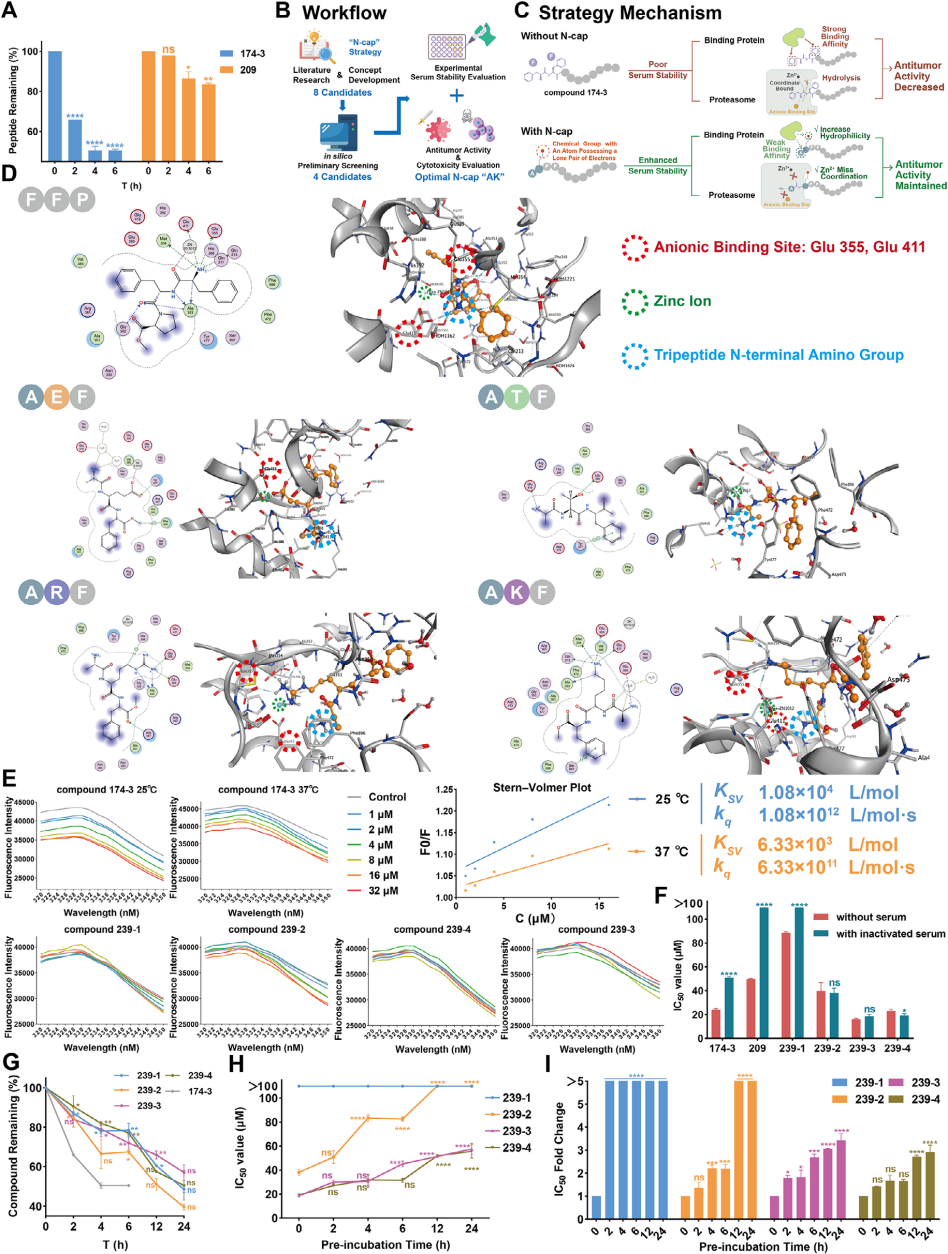
A) Peptide remaining (%) of compound 174‐3 and conjugate 209 after incubation with human serum for up to 6 h. B) Workflow of N‐cap development process. C) Schematic illustration of the proposed N‐cap mechanism. D) Predicted docking 2D interaction patterns (left) and 3D cartoon interaction patterns (right) between APN and tripeptides, FFP, AEF, ATF, ARF, and AKF. In 3D cartoon interaction, receptors are gray, and ligands are orange. Two anionic binding sites, Glu355 and Glu411, are highlighted by red circle, zinc ion is highlighted by green circle and the N‐terminal amino group of tripeptides is highlighted by blue circle. Blue dashed lines represent hydrogen bonds. For interpretation of the legend in the 2D interaction patterns, refer to Figure S38A (Supporting Information). E) Fluorescence emission spectra (320–350 nm) of 20 µm of human serum albumin (HSA) treated with different compounds at different concentrations. The tested temperature of compounds 239‐1–239‐4 was 25 °C. Stern–Volmer plot and *K*_SV_ and *k*_q_ values of compound 174‐3 at different tested temperatures are presented. F) IC_50_ values of peptides 174‐3, 209, and 239‐1–239‐4 against H460 cells, with or without preincubation in heat‐inactivated serum. G) Compound remaining (%) of compounds 239‐1, 239‐2, 239‐3, and 239‐4 preincubated with human serum for up to 24 h, compound 174‐3 served as control group. H) IC_50_ values comparison of compounds 239‐1–239‐4 on H460 cell line after preincubation with human serum for up to 24 h. I) Fold change comparison in IC_50_ values of compounds 239‐1–239‐4 on H460 cells after preincubation with human serum for up to 24 h. Data represent the mean ± SEM, *n* = 2 (A and G) or *n* = 3 (F, H, and I). One‐way ANOVA followed by Dunnett's post hoc test was used to compare each compound group with compound 174‐3 group at the same point (2, 4, and 6 h) or with the remaining (%) of compound 174‐3 at 6 h (12 and 24 h) (G), or compare each time point with the corresponding 0 h of the same compound (A, H, and I). An unpaired two‐tailed *t*‐test was used to compare treatments in serum versus without serum (F). ns for *p* > 0.05, * for *p* < 0.05, ** for *p* < 0.01, *** for *p* < 0.001, and **** for *p* < 0.0001.

Following the resolution of selectivity concerns, we next addressed serum instability, a major obstacle for AMP‐based therapeutics, typically caused by proteolytic degradation and nonspecific binding to serum proteins.^[^ ^29^ ^]^ To investigate this, we assessed both the functional and structural stability of compound 174‐3 and conjugate 209 in human serum (**Figure** **4**A; Table S2, Supporting Information). Upon immediate exposure to inactivated serum (0 h), the IC_50_ of compound 174‐3 doubled and conjugate 209 completely lost its antiproliferative activity, indicating a strong interaction with serum proteins rather than enzymatic degradation. However, after 2 h in active serum, compound 174‐3 also lost cytotoxicity. HPLC and MS/MS analyses revealed varying degrees of degradation for the two peptides after 6 h incubation, with cleavage at the N‐terminal Phe^1^ in both cases, suggesting involvement of aminopeptidase (Figures S40–S45, Tables S3,S4, Supporting Information). These observations, combined with literature reports that peptide–albumin binding is driven predominantly by hydrophobic moieties and that leucine and alanine aminopeptidases can broadly cleave N‐terminal residues due to low substrate specificity,^[^ ^30^, ^31^, ^32^ ^]^ highlight the dual challenges of protein binding and enzymatic attack to the N‐terminus of AMP. Conventional solutions such as PEGylation and lipidation often fall short: PEG reduces AMP activity, while lipidation increases off‐target toxicity.^[^ ^33^, ^34^ ^]^

To overcome these limitations, we developed an “N‐terminal capping” strategy. This strategy was informed by the hydrolysis mechanism of zinc‐dependent aminopeptidases, which coordinate a zinc ion with the carbonyl oxygen of the first amide bond and engage the N‐terminal amine via anionic residues such as Asp or Glu.^[^ ^35^, ^36^ ^]^ We considered that introducing a nearby functional group bearing a lone pair of electrons could interfere with zinc coordination and block hydrolysis. Based on this rationale, a dipeptide called “N‐cap” for attachment to the peptide N‐terminus was developed: alanine was selected as the first residue to maintain low hydrophobicity, and the second residue carried a polar side chain capable of disrupting zinc coordination and reducing serum protein binding. This dual‐action cap was expected to enhance both proteolytic resistance and serum compatibility, while minimally impacting the original bioactivity and toxicity profile. The development contained in silico and wet experiment two screening processes (Figure 4B,C).

#### 2.4.2 From In Silico Screening to Experimental Validation: Ala–Lys Emerges as the Optimal N‐Cap

To identify the optimal amino acid for the second position of the N‐cap, we shortlisted natural residues with side chains containing lone electron pairs (─COOH, ─CONH_2_, ─NH_2_, or ─OH), including Asp, Glu, Asn, Gln, Lys, Arg, Ser, and Thr. Molecular docking was employed to evaluate their capacity to interfere with aminopeptidase activity (Figure 4D; Figures S46,S47, Supporting Information). As structural models, we used human aminopeptidase N (APN, protein data bank (PDB): 4FYT) and bovine lens leucine aminopeptidase (LAP, PDB: 1BLL), serving as analogs for alanine and leucine aminopeptidases, respectively.^[^ ^37^, ^38^ ^]^ To ensure reliable docking results, only the first three amino acids of the N‐capped compound 174‐3 were used, resulting in the following tripeptide candidates: ADF, AEF, ANF, AQF, AKF, ARF, ASF, ATF, and the original FFP. Initial docking with FFP showed correct binding to the APN active site, including interactions with all key amino acids and the zinc ion, but it failed to bind correctly with LAP. This indicated that FFP would likely be hydrolyzed by alanine aminopeptidase but not leucine aminopeptidase. According to the predicted docking model of FFP bound to APN, two key anionic binding sites, Glu355 and Glu411 need have interaction with the N‐terminal amino group of FFP, and a coordinate bound was formed between zinc ion and the carbonyl oxygen of the first amide bond at the peptide N‐terminus. Subsequent docking of the remaining tripeptides with APN identified candidates whose chemical group with a lone pair of electrons interacted with zinc ion, key anionic binding sites, or other anionic amino acids near the binding pocket, and which induced zinc ion miscoordination to resist hydrolysis. Based on the in silico screening, four tripeptides (AEF, ATF, ARF, and AKF) were identified and further docked with LAP, where similar results confirmed that they were resistant to hydrolysis by LAP. Consequently, four N‐caps (AE, AT, AR, and Ala–Lys (AK)) were selected for the wet experiment screening.

Four N‐capped analogs of 174‐3 (compounds 239‐1–239‐4) were synthesized to evaluate the effect of different N‐cap structures on serum stability, antitumor efficacy, and cytotoxicity. First, fluorescence quenching assays were performed to evaluate the binding of compounds to HSA, the most abundant protein in human plasma. Compound 174‐3 exhibited concentration‐dependent quenching of HSA fluorescence, with stronger effects at 25 than 37 °C. The calculated 𝐾_SV_ values (1.08 × 10⁴ and 6.33 × 10^3^ L mol^−1^) and high 𝑘_𝑞_ values (>10^11^ L mol^−1^ s^−1^) exceeded the diffusion limit, indicating static quenching.^[^ ^39^ ^]^ This suggests that compound 174‐3 formed a ground‐state complex with HSA, while compounds 239‐1–239‐4 displayed minimal or no quenching, implying negligible binding (Figure 4E). Next, the long‐term serum stability of N‐capped compounds was evaluated by incubating peptides in 50% human serum for up to 24 h. Analysis of degradation products revealed that compounds 239‐2 and 239‐3 primarily lost the ATF tripeptide and AR dipeptide, respectively, suggesting that introducing Thr or Arg at the second position of the N‐cap effectively prevented the cleavage induced by aminopeptidase (Figures S51–S56, Tables S6,S7, Supporting Information). By contrast, compounds 239‐1 and 239‐4 still underwent N‐terminal Ala removal (Figures S48–S50,S57–S59, Tables S5,S8, Supporting Information). Additionally, all analogs exhibited slower degradation than compound 174‐3 (Figure 4G). These findings illustrated that N‐cap could effectively inhibit or prevent aminopeptidase‐mediated hydrolysis to AMPs. Subsequently, all compounds were either untreated or preincubated with human serum for up to 24 h prior to evaluation of their antiproliferative activity against H460 cells (Table S2, Supporting Information). Compound 239‐3 displayed the best efficiency against H460 cells without serum incubation(IC_50_: 14.69 µm), followed by compound 239‐4 (IC_50_: 22.22 µm) and 239‐2 (36.83 µm), while compound 239‐1 (IC_50_: 88.23 µm) lost major activity due to the positive charge decrease induced by Glu. The results of all compounds incubated with heat‐inactive serum (0 h) demonstrated that compounds 239‐2, 239‐3, and 239‐4 exhibited significantly improved activity retention compared to compound 174‐3 and conjugate 209 (Figure 4F). With the preincubation time extension, the IC_50_ values of those three compounds all increased (Figure 4H). The activity of compound 239‐2 significantly decreased after 4 h preincubation and totally lost after 12 h preincubation. By contrast, the antitumor capabilities of compounds 239‐3 and 239‐4 were much stable. Even after 24 h preincubation, the IC_50_ values of them could be detected (≈60 µm). The IC_50_ fold‐change of compound 239‐4 was the lowest among four compounds, which was no more than 3 after up to 24 h preincubation (Figure 4I). Further toxicity evaluation demonstrated that compound 239‐3 displayed the highest toxicity against HEK‐293 cells and erythrocytes among four compounds, while compound 239‐4 contained similar toxicity to HEK‐293 cells but stronger hemolytic activity compared to compound 174‐3. Compounds 239‐1 and 239‐2 presented much lower toxicity (Table S1, Supporting Information). Collectively, compound 239‐4 emerged as the optimal candidate, balancing enhanced serum stability and antitumor efficacy with minimal increase in toxicity.

To further verify the essential role of the Lys in the N‐cap of compound 239‐4, a control analog (239‐4‐Ac) was synthesized by acetylating the ε‐amino group of Lys^2^. Molecular docking with APN indicated that this modification disrupted key interactions necessary for hydrolytic resistance. Consistently, peptide stability assays in human serum revealed that compound 239‐4‐Ac was significantly more prone to degradation than compound 239‐4, with its degradation profile closely resembling that of compound 174‐3 (Figures S60–S64, Tables S9,S10, Supporting Information).

Collectively, Section 2.4 demonstrated that the “N‐terminal capping” strategy enhances AMP serum stability by preventing aminopeptidase cleavage and reducing nonspecific protein binding. Guided by the hydrolysis mechanism of zinc‐dependent enzymes, in silico screening identified AK as the optimal cap. The resulting analog, compound 239‐4, showed improved proteolytic resistance and similar antitumor efficacy with minimal toxicity increase. Mechanistic studies confirmed the essential role of the Lys side chain, establishing N‐cap as a generalizable approach to optimize AMP pharmacokinetics.

### 2.5 Therapeutic Profile and Mechanistic Evaluation of Conjugate 270

**Figure 5:**
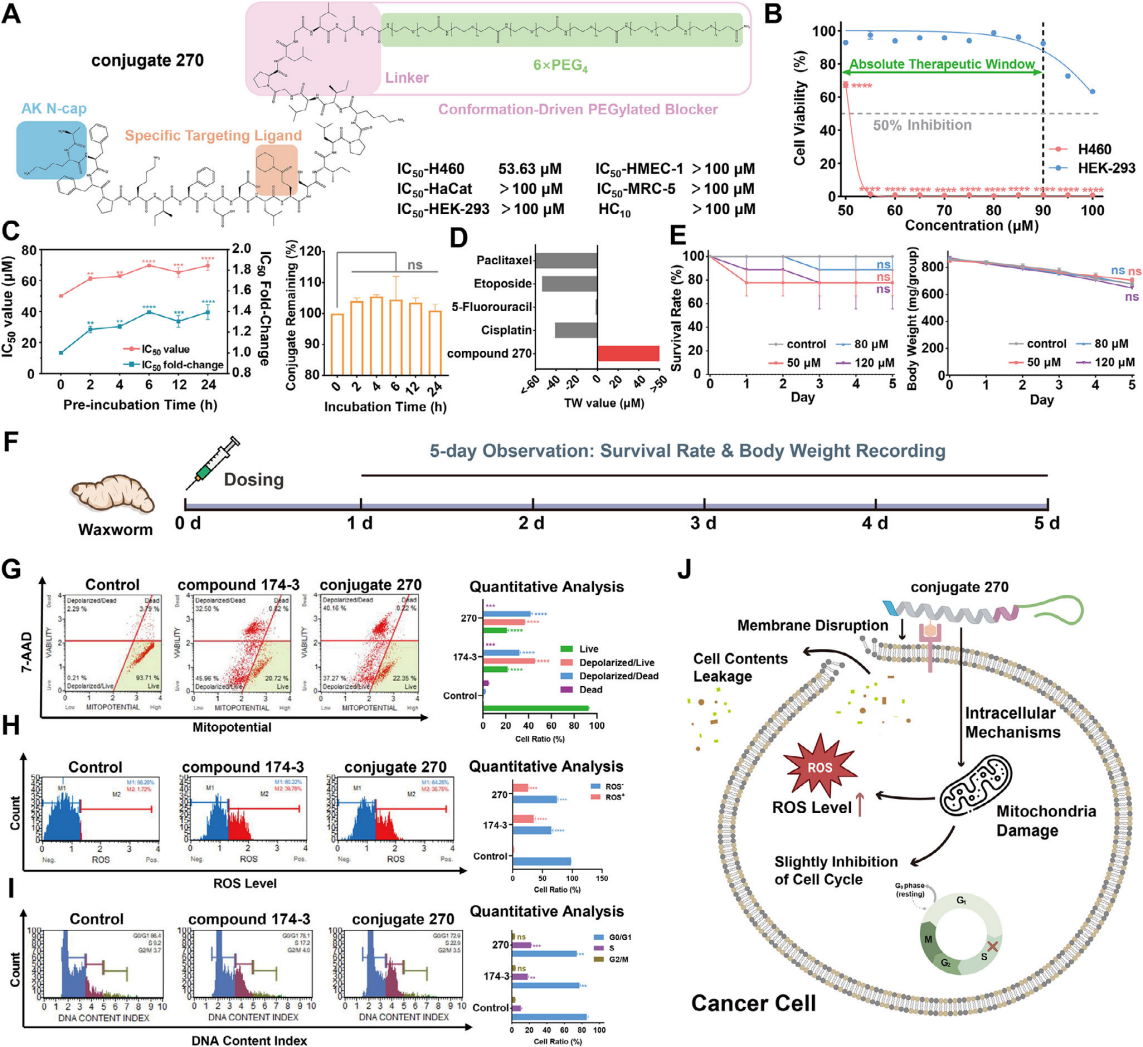
A) Chemical structure and antiproliferative activity of conjugate 270 against different cell lines. B) Antiproliferative effect of conjugate 270 against H460 and HEK‐293 cell lines at series of concentrations. Absolute therapeutic window is included. C) IC_50_ values and fold change against H460 cell line (left) and conjugate remaining (%) (right) of conjugate 270 after preincubation or incubation with human serum for up to 24 h. D) TW value comparison between conjugate 270 and four first‐line chemotherapeutics. E) In vivo safety evaluation of conjugating 270 using waxworm. Survival rate (left) and body weight (right) are presented. F) The whole process is schemed. G) MitoPotential/7‐AAD flow cytometry analysis of H460 cells treated with compound 174‐3 and conjugate 270 at IC_50_ value. H) Reactive oxygen species (ROS) ‐level‐based flow cytometry analysis of H460 cells treated with compound 174‐3 and conjugate 270 at IC_50_ value. I) DNA‐content‐index‐based flow cytometry analysis of H460 cells treated with compound 174‐3 and conjugate 270 at IC_50_ value. J) Brief scheme illustrating the antitumor mechanisms of conjugate 270 against H460 cells. Data represent as mean ± SEM, *n* = 2 (C right) or *n* = 3 (B, C left, E, G–I). One‐way ANOVA followed by Dunnett's post hoc test was used to compare each treatment group with the 0 h preincubation or incubation group (C), the control group at day 5 (E), or the control group (G–I). Unpaired two‐tailed *t*‐test was used to compare the treatment group against H460 cells with the treatment group against HEK‐293 cells at the same concentrations (B). ns for *p* > 0.05, ** for *p* < 0.01, *** for *p* < 0.001, and **** for *p* < 0.0001.

The AK N‐cap was incorporated at the N‐terminus of conjugate 209, resulting in conjugate 247. However, antiproliferative assays revealed that it retained significant cytotoxicity against the HEK‐293 cell line (Table S1, Supporting Information). To address this, the amount of conjugated PEG_4_ was increased from 4 to 6, yielding the final conjugate, 270 (**Figure** **5**A). Conjugate 270 demonstrated ideal antiproliferative activity against the H460 target cell line, with an IC_50_ value of 53.63 µm and an IC_95_ value of ≈55 µm. Notably, no inhibitory or disruptive effects were observed on HMEC‐1, HaCaT, MRC‐5, or erythrocytes at tested concentrations (Table S1, Supporting Information). Growth inhibition of HEK‐293 cells was only observed at concentrations ≥95 µm, with 40% inhibition at 100 µm (Figure 5B). This indicated an absolute therapeutic window (ATW) of 40 µm, within which tumor cells were completely eradicated without harming normal cells. LDH assays confirmed conjugate 270 maintained a mighty membrane disruptive capability against H460 cells but lacked strong membrane disruption on normal cells at all potency levels, with a more than 65% difference in LDH release between H460 and normal cell lines (Figure S65A, Supporting Information). Additionally, conjugate 270 exhibited exceptional serum stability, with an IC_50_ fold increase of only 1.4 and nearly 100% remaining ratio after 24 h of preincubation in serum (Figure 5C). These results highlight the ideal membrane selectivity of 270, resulting in minimal cytotoxicity to normal cells at effective concentrations, alongside robust resistance to hydrolysis in human serum. Given the lack of effective treatments for LCLC, we compared the therapeutic profile of conjugate 270 with four first‐line chemotherapeutics (cisplatin, 5‐fluorouracil, paclitaxel, and etoposide). TW values were calculated as the difference in IC_50_ values between H460 and HEK‐293 cells. Notably, 270 was the only one with a positive TW value exceeding 50, indicating superior safety (Figure 5D; Table S11, Supporting Information). Moreover, its IC_95_ value against the H460 cell line was only one‐third to one‐eighth of the IC_50_ values of conventional chemotherapeutics, highlighting its superior potency. Due to limitations in animal use and in alignment with the 3R principle, we employed *Galleria mellonella (G. mellonella)* larvae to preliminarily assess the in vivo safety of conjugate 270 (Figure 5E,F; Figure S65B, Supporting Information). This model offers credible correlation with mammalian outcomes and allows rapid toxicity screening.^[^ ^40^, ^41^, ^42^ ^]^ Larvae were injected with 50, 80, or 120 µm of conjugate 270. After 5 days, survival remained high across all groups: 77.8% for both 50 and 120 µm, and 88.9% for 80 µm. No melanization was observed at any dose, indicating a lack of systemic toxicity or immune overactivation. Larval weights declined gradually in all groups, including controls, consistent with expected fasting‐induced loss rather than compound‐related effects. No dosing group displayed accelerated weight loss. These results suggested that conjugate 270 was well tolerated at pharmacologically relevant doses, with no signs of acute toxicity, inflammation, or physiological stress in vivo.

We further investigated the antitumor mechanism of conjugate 270 (Figure 5J). Using the same approach described in Section 2, we confirmed that its mechanism mirrored that of compound 174‐3, inducing rapid membrane disruption in H460 cells (Figure S65C–G, Supporting Information). To explore potential downstream effects, we employed mitochondrial membrane potential, ROS, and cell cycle assays on two peptides (Figure 5G–I). The results revealed that conjugate 270 induced pronounced mitochondrial depolarization. Concurrently, a significant increase in intracellular ROS was observed in dosing groups, indicating that oxidative stress was actively involved in the cell death process. This suggested that mitochondrial dysfunction was likely driven by a combination of direct membrane disruption and ROS‐mediated respiratory damage. Cell cycle analysis displayed a modest increase in S‐phase cell population, consistent with observations from compound 174‐3. We speculated that this may result from mitochondrial dysfunction leading to ATP depletion and impaired DNA synthesis. However, this effect was likely secondary and not the primary mechanism underlying its antitumor activity.

### 2.6 Functional Validation of Rapid Killing and Chemoresistant Tumor Inhibition

**Figure 6:**
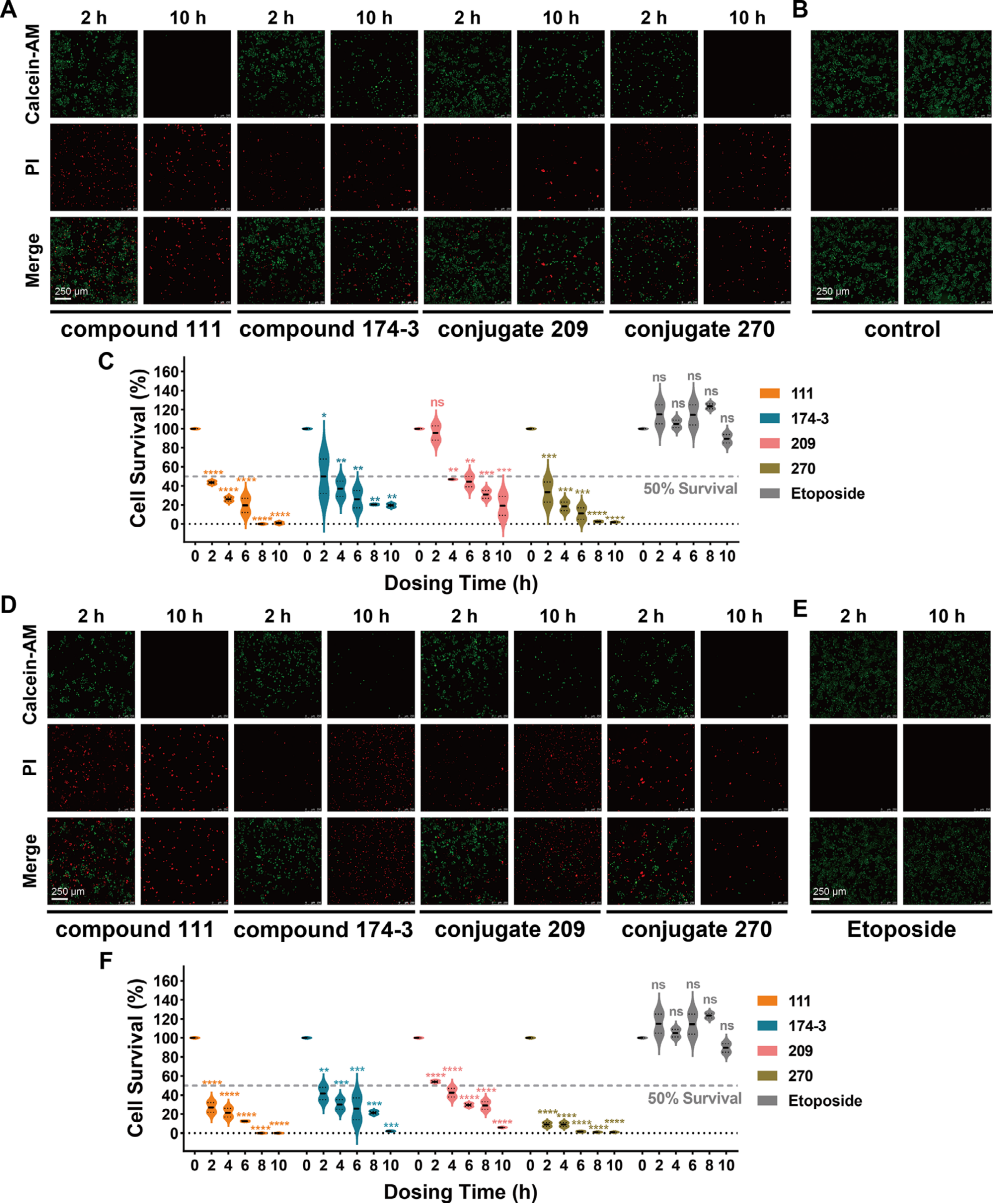
Killing rate evaluation of four peptides against H460 cells using calcein‐AM/PI double staining. Scale bar = 250 µm. To facilitate comparison, the 2 and 10 h time points for each peptide are presented. Full original images are provided in Figure S68 (Supporting Information). The dosing concentration of four peptides in (A) was IC_50_ and in (D) was IC_95_. B) The control group was treated with 0.5% dimethyl sulfoxide (DMSO) in serum free medium. Quantitative analysis at IC_50_ values (C) or IC_95_ values () are presented. E) The dosing concentration of etoposide group in each quantitative analysis figure was IC_50_. Data represent the mean ± SEM, *n* = 2. Statistical analysis was performed using one‐way ANOVA with Dunnett's post hoc test, comparing each treatment group with the 0 h dosing group of the corresponding peptide; ns = not significant, *p < 0.05, **p < 0.01, ***p < 0.001.

Given that the unique membrane‐disruption mechanism was preserved throughout the conjugate development and optimization process, we sought to investigate whether the key intermediate compound and the final compound retained the rapid‐killing property characteristic of typical AMPs. To this end, the killing rates of peptides 111, 174‐3, 209, and 270 at their IC_50_ and IC_95_ concentrations against the H460 cell line with etoposide serving as the control. The **c**alcein‐AM/PI staining assay results demonstrated that all compounds achieved full efficacy within 10 h, significantly outperforming etoposide, which only eliminated ≈10% of cells within the same time frame (**Figure** **6**). Notably, conjugate 270 displayed fast rate, killing ≈90% of cells within 2 h at its IC_95_ concentration. Across all compounds, killing rates were markedly faster at IC_95_ concentrations compared to IC_50_ concentrations.

Finally, we hypothesized that AMPs may exhibit activity against chemotherapeutic‐resistant (chemoresistant) tumors due to their membrane‐targeting mechanism of action, which bypasses intracellular resistance pathways. To proof this, we developed four chemoresistant H460 cell lines: cisplatin‐resistance, 5‐fluorouracil‐resistance, paclitaxel‐resistance (DR‐PTX), and etoposide‐resistance (DR‐EPT), each exhibiting high resistance to a specific chemotherapeutic agent across all tested concentrations, as confirmed by antiproliferative assays (Figure S69A, Supporting Information). Subsequently, peptides 111, 174‐3, 209, and 270 were assessed for their inhibitory and membrane‐disruptive capabilities against these resistant cell lines (**Table** **1**; Figure S69B,C, Supporting Information). The results aligned with our expectations. Except for conjugate 209, which lacked inhibitory activity against DR‐EPT cell line, all compounds demonstrated varying degrees of inhibition across the chemoresistant cell lines. Interestingly, conjugate 270 exhibited lower IC_50_ values against DR‐PTX and DR‐EPT cells compared to the normal H460 cells, indicating enhanced inhibitory activity against these resistant variants. LDH release assays confirmed that the antitumor mechanism of all four compounds remained membrane disruption, consistent with expectations. Although the underlying differences in antiproliferative and membrane‐disruptive activity between the normal and chemoresistant H460 cell line remain unclear, these findings suggest that our conjugate holds promise as potential therapeutic agent for treating drug‐resistant tumors.

**Table 1:** Antiproliferative activity of peptides 111, 174‐3, 209, and 270 against four chemoresistant H460 cell lines.

	IC_50_ value [µm]			
	Cisplatin‐resistance	5‐Fluorouracil‐resistance	Paclitaxel‐resistance	Etoposide‐resistance
111	5.24 ± 0.15	5.14 ± 0.22	5.54 ± 0.04	5.17 ± 0.16
174‐3	29.25 ± 2.29	28.87 ± 3.53	24.15 ± 2.13	17.68 ± 2.86
209	94.30	84.49 ± 4.55	97.83	>100
270	54.73 ± 3.28	56.44 ± 0.25	35.31 ± 1.83	28.06 ± 1.42

### 2.7 Advantages and Limitations

Compared with existing AMP‐based anticancer models, our system exhibits several significant improvements. First, instead of relying solely on peptide sequence modification or a single optimization strategy, we adopted a rational three‐step design, membrane affinity reconstruction, linker activation, and N‐terminal capping, to systematically improve selectivity and stability. This approach allows us to retain the membrane‐targeting mechanism of classical AMPs while minimizing off‐target toxicity and proteolytic degradation, thereby overcoming common limitations of AMP‐based therapies. Although comparable studies are limited, we identified three representative examples to contextualize our results. Chen et al.^[^ ^16^ ^]^ conjugated PEG chains of varying lengths to melittin. While toxicity was reduced to some extent, the antitumor activity was also significantly compromised, failed achieving an ideal selectivity against tumor cells. Saleh et al.^[^ ^43^ ^]^ used BMPA27 to conjugate melittin. However, due to its classical apoptosis‐based mechanism, the resulting conjugate did not demonstrate an ATW as seen with conjugate 270, resulting in selectivity toward tumor over normal cells remained extremely limited. Lu et al.^[^ ^15^ ^]^ constructed PEG–MP9–aPDL1 by coupling a stapled oncolytic peptide with a PD‐L1‐targeting ligand via a MMP‐2‐cleavable linker. While the conjugate showed immune‐potentiated efficacy, its dependence on PD‐L1 expression limited broader tumor applicability. By contrast, conjugate 270 functions independently of immune checkpoint status, and its ligand was modularly designed to allow replacement based on different tumor targets. In addition, conjugate 270 contains a simpler synthesis route and lower production cost compared to PEG–MP9–aPDL1. Second, conjugate 270 remained highly effective against multiple chemoresistant cell lines, supporting its potential to overcome multidrug resistance via a membrane‐disruptive mechanism that bypasses conventional intracellular targets, a capability rarely reported in current studies.

Despite these strengths, our study has several limitations. First, the incorporation of unnatural amino acids (Glu(PIP)) may pose immunogenicity risks. Although TNF‐α secretion assays in RAW264.7 macrophages showed no significant proinflammatory activation, and no melanization was observed in *G. mellonella* larvae following systemic administration, suggesting an absence of overt immune responses, comprehensive immunogenicity evaluation is still warranted. Future studies should include immunoinformatic prediction of T‐ and B‐cell epitope profiles, followed by in vivo assessment of systemic immune activation and antibody production. Second, due to laboratory constraints, in vivo validation in murine tumor models was not conducted. Instead, we employed the *G. mellonella* larvae model as a preliminary tool for systemic toxicity assessment. While increasingly used in early stage screening, this model does not substitute for mammalian pharmacokinetics, efficacy, or organ‐specific safety evaluation. Notably, although conjugate 270 exhibited excellent selectivity at therapeutic concentrations, higher doses (100 µm) induced moderate cytotoxicity in HEK‐293 kidney cells, suggesting a potential risk under overdose or renal accumulation conditions. Future studies should include rodent‐based dose escalation and organ toxicity profiling to establish a clinically safe exposure range. Finally, further validation across a broader panel of cancer cell lines and membrane receptors in the future is needed to assess the generalizability of our AMP‐based platform.

## 3 Conclusion

This study presents a structurally optimized AMP–drug conjugate, 270, that overcomes key limitations of existing AMP‐based anticancer models. By integrating membrane affinity reconstruction, conformation‐driven PEGylated blocker, and unnatural N‐terminal capping, conjugate 270 achieves selective membrane disruption and exceptional serum stability, without compromising potency. Its 40 µm absolute TW enables complete tumor cell eradication with minimal impact on normal cells. Mechanism studies confirmed a membrane‐driven killing mode, while *G. mellonella* data supported preliminary in vivo safety. Together, these findings establish a robust framework for AMP functionalization and highlight conjugate 270 as a promising candidate for precision therapy against hard‐to‐target or drug‐resistant tumors. Beyond its immediate therapeutic potential, this study establishes a versatile and adaptable platform for AMP‐based targeted therapies, providing a blueprint for expanding peptide therapeutics to previously untreatable cancers. By addressing the fundamental limitations of AMPs, this strategy sets a new benchmark in the field and lays a strong foundation for future clinical translation.

## 4 Experimental Section

### Chemistry—General Information

Fluorenylmethoxycarbonyl (Fmoc)─Glu(OAll)─OH, Fmoc─Lys((4‐methylphenyl)‐diphenylmethyl (Mtt))─OH, ethyl 2‐cyano‐2‐(hydroxyimino)acetate (Oxyma), 5,8,11,14‐Tetraoxa‐2‐azaheptadecanedioic acid 1‐(9*H*‐fluoren‐9‐ylmethyl) ester, and FITC were purchased from Fluorochem (UK). All natural amino acids used in the synthesis were purchased from Novabiochem (Merck, USA). *O*‐(Benzotriazol‐1‐yl)‐*N*,*N*,*N′*,*N′*‐tetramethyluronium hexafluorophosphate (HBTU) was purchased from GYROS PROTEIN Technologies (Sweden). Other chemicals were purchased from Sigma‐Aldrich (Merck, USA). All reagents were used without further purification. The ultrasonic agitation was performed through Fisher Scientific FB15049 Ultrasonic Bath (Thermo Fisher Scientific, USA) and the temperature for water bath was 30 °C.

### Purification and Characterization

Compounds were purified by preparative HPLC (Waters 1525 binary pump, 2489 UV–vis detector; Phenomenex Aeris5 µm PEPTIDE XB‐C18, 250 × 21.2 mm) using a gradient from 70% solvent A/30% solvent B to 100% solvent B over 90 min (solvent A: H_2_O/0.1% trifluoroacetic acid (TFA); solvent B: acetonitrile/0.1% TFA). Flow rate was 5 mL min^−1^ and detection at 214 nm. Purity was confirmed by analytical HPLC (Waters 1525 binary pump, 2489 UV–vis detector, 2707 autosampler; Vydac C18, 300 Å, 150 × 4.6 mm). The gradient was from 100% solvent A to 100% solvent B in 60 min. The flow rate was 1 mL min^−1^, and the wavelength of UV detector was 214 nm. The primary and tandem mass spectra were measured on Thermo Scientific LCQ Fleet Ion Trap LC/MS^n^ (Thermo Fisher Scientific, USA).

### General Synthetic Method

All compounds were synthesized using solid‐phase peptide synthesis. The detailed synthetic processes for each compound were given in the Supporting Information.

### Resin Swelling

Rink amide MBHA resin (128.2 mg, 0.1 mmol, 100–200 mesh, substitution: 0.78 mmol g^−1^) was placed in a 25 mL solid‐phase synthesis tube and swollen in 15 mL of *N*,*N*‐dimethylformamide (DMF) on a shaker for 1 h. The solvent was then removed by vacuum filtration.

### Resin Washing

DMF (3.3 mL) was added to the synthesis tube and the mixture was ultrasonically agitated for 1 min. Afterward, the DMF was removed.

### Fmoc Group Deprotection

Resin was treated with 3.3 mL of 20% PIP in DMF solvent and the mixture was ultrasonically agitated for 3 min. Subsequently, the resin was washed twice with DMF.

### Amino Acid Coupling

Fmoc‐protected amino acid (0.3 mmol), HBTU (114 mg, 0.3 mmol), and Oxyma (44 mg, 0.3 mmol) were dissolved in DMF (4.4 mL) and dichloromethane (DCM) (2.2 mL) in a 50 mL centrifuge tube. Next, *N*,*N*‐diisopropylethylamine (DIPEA) (78 µL, 0.45 mmol) was added, and the mixture was shaken for 5 min to activate the carboxyl group. Subsequently, the mixture was transferred to the solid‐phase synthesis tube and ultrasonically agitated with resin for another 10 min. Then, the resin was washed twice.

### Resin Washing and Drying

Ten mL of anhydrous methanol was added to the synthesis tube and the mixture was ultrasonically agitated twice, each time for 30 s. Subsequently, the resin was dried by vacuum evacuation for 1 h.

### Resin Cleavage

The dried resin was transferred into a 50 mL round‐bottom flask and 10 mL cleavage solvent containing 95% TFA (9.5 mL), 2.5% triisopropylsilane (TIS) (250 µL), and 2.5% H_2_O (250 µL) was added in the flask. The mixture was stirred for 2 h. Afterward, the cleavage solvent was separated from the resin by filtration and concentrated by an evaporator. Then, a tenfold volume of ‐20 °C diethyl ether was added in the concentrated solution to precipitate the crude product.

### Ally (OAll) Group Deprotection

Tetrakis(triphenylphosphine)palladium (36 mg, 0.03 mmol) and phenylsilane (62 µL, 0.5 mmol) were dissolved in DCM (4 mL). This solution was added to the synthesis tube and shaken with resin for 30 min. After removal of the solvent, a freshly prepared solution of the same composition was added, and the resin was shaken for another 30 min. Finally, the resin was washed thoroughly with DMF 6 times.

### Mtt Group Deprotection

The resin was shaken twice for 5 min with 5 mL of deprotection solution containing 5% TFA (250 µL), 5% TIS (250 µL), and 90% DCM (4.5 mL). Then, the resin was washed 6 times.

### PIP Group Conjugation

PIP (30 µL, 0.3 mmol), HBTU (114 mg, 0.3 mmol), and Oxyma (44 mg, 0.3 mmol) were dissolved in DMF (4.4 mL) and DCM (2.2 mL). DIPEA (78 µL, 0.45 mmol) was added, and the mixture was transferred to the synthesis vessel and ultrasonically agitated with the resin for 15 min. Afterward, the resin was washed 3 times.

### Acetylation

Five mL of acetic anhydride and 1 mL of pyridine were added to the synthesis tube and shaken with the resin for 2 h. After that, the resin was washed 6 times.

### FITC Group Conjugation

FITC (156 mg, 0.4 mmol) and DIPEA (50 µL, 0.29 mmol) were dissolved in 5 mL of DMF and shaken with the resin overnight. Subsequently, the resin was washed 6 times.

### Biological Evaluation and Mechanism Detection—General Information

RPMI‐1640 (1640) medium, Dulbecco's modified Eagle's medium (DMEM), minimum essential medium (MEM), fetal bovine serum (FBS), penicillin streptomycin (Ps), trypsin–ethylenediaminetetraacetic acid (EDTA) (0.5%), Earle's balanced salt solution (EBSS), and l‐glutamine were purchased from Gibco (Thermo Fisher Scientific, USA). 3‐(4,5‐Dimethyl‐2‐thiazolyl)‐2,5‐diphenyl‐2*H*‐tetrazolium bromide (MTT), trypan blue solution, cytotoxicity detection kit (LDH), human epidermal growth factor (hEGF), hydrocortisone, cisplatin, 5‐fluorouracil, etoposide, paclitaxel, piperine, probenecid, MMP‐2/MMP‐9 Inhibitor IV, Triton X‐100, human serum (from human male AB plasma, US origin), propidium iodide (PI), Calcein‐AM, 4′,6‐diamidino‐2′‐phenylindole (DAPI), SynaptoRed reagent (FM4‐64), and other reagents were purchased from Sigma‐Aldrich (Merck, USA). Defibrinated horse blood was purchased from TCS Biosciences Ltd. (Buckingham, UK). Complete growth medium (CGM) consisted of 89% medium, 10% FBS, and 1% Ps. Serum‐free medium (SFM) consisted of 95% medium and 5% Ps. NCI‐H460, NCI‐H838, and PC‐3 cell lines were cultured with 1640 CGM. HT‐1080, MCF‐7, U‐251 MG, U‐87 MG, RAW264.7, HaCaT, and MRC‐5 cell lines were cultured with DMEM CGM. HEK‐293 cell line was cultured with MEM CGM. HMEC‐1 cell line was cultured with special DMEM CGM, which consisted of 85% DMEM, 10% FBS, 5% Ps, 10 ng mL^−1^ of hEGF, and 1 µg mL^−1^ of hydrocortisone. All cell lines were cultured at 37 °C with 5% CO_2_. The absorbance and fluorescence intensity were measured by a Synergy HT plate reader (Bio Tek, Winooski, VT, USA). Human serum was centrifuged at 14 000 × *g* for 20 min at 4 °C to remove the lipid content.

### Cell Antiproliferation Evaluation

MTT assay was conducted as previously described.^[^ ^44^ ^]^ Tumor cell lines (NCI‐H460, NCI‐H838, HT‐1080, MCF‐7, PC‐3, U‐251‐MG, and U‐87 MG) were seeded in 96‐well plates at a density of 8000 cells per well in a volume of 100 µL and cultured for 24 h. Normal cell lines (HaCaT, HMEC‐1, MRC‐5, and HEK‐293) were seeded in 96‐well plates at a density of 10 000 cells per well in a volume of 100 µL and cultured for 48 h. After that, only tumor cell lines were starved with 100 µL of SFM for a further 4–6 h. Subsequently, cells were treated with 100 µL of compound solutions with different concentrations for 24 h. Cells treated with SFM and 0.5% DMSO in SFM served as blank control and growth control, respectively. Ten µL of MTT solution (5 mg mL^−1^) was added to each well, followed by an additional incubation period of 2 h for tumor cells and 4 h for normal cells. Finally, the solvent in each well was swapped with 100 µL of DMSO to dissolve the formazan crystals. The absorbance was measured by the plate reader at 570 nm.

The cell viability was calculated

(1) $$\begin{array}{ccc} \mathrm{Cell}\;\mathrm{viability}\left(\%\right) & = & \left({\mathrm{OD}}_{\mathrm{Sample}\;\mathrm{group}}-{\mathrm{OD}}_{\mathrm{Blank}}\right)\\ & & /\left({\mathrm{OD}}_{\mathrm{Growth}\;\mathrm{group}}-{\mathrm{OD}}_{\mathrm{Blank}}\right)\times 100\% \end{array}$$

### Hemolysis Assays

Defibrinated horse blood (1 mL) was washed 3 times with 0.9% NaCl solution to remove plasma and then resuspended in 20 mL of 0.9% NaCl solution to obtain a red blood cell (RBC) suspension. Aliquots of the RBC suspension (100 µL) were incubated with 100 µL of compound solutions at concentrations ranging from 100 to 1.5625 µm (twofold serial dilution) for 2 h at 37 °C. RBCs treated with 0.9% NaCl solution and 1% Triton X‐100 solution served as blank and positive controls, respectively. Subsequently, the samples were centrifuged at 900 × *g* for 10 min, and the absorbance of the supernatant was measured at 570 nm.

The hemolysis ratio was calculated as

(2) $$\begin{array}{ccc} \mathrm{Hemolysis}\left(\%\right) & = & \left(OD_{\mathrm{Sample}\;\mathrm{group}}-OD_{\mathrm{Blank}}\right)\\ & & /\left(OD_{\mathrm{Positive}\;\mathrm{group}}-OD_{\mathrm{Blank}}\right)\times 100\% \end{array}$$

### Killing Rate Evaluation

Calcien‐AM/PI double staining assay was used. NCI‐H460 cells were seeded in a chamber‐slide (154526, Thermo Fisher Scientific) at a density of 100 000 cells per unit in a volume of 500 µL and cultured for 24 h. After starvation with 300 µL of SFM for 4 h, cells were treated with 500 µL of compound solutions with different concentrations for 2, 4, 6, 8, 10 h, respectively. Cells treated with 0.5% DMSO in SFM served as control group. Subsequently, cells in each unit were washed with EBSS once, and incubated with 500 µL of 2 µg mL^−1^ calcien‐AM solution at 37 °C for 20 min. Afterward, cells were stained with 500 µL of 2 µg mL^−1^ PI solution at room temperature for 15 min and washed with EBSS 3 times. Finally, 300 µL of EBSS was added into each unit. Live (green fluorescence from calcein‐AM) and dead (red fluorescence from PI) cells were then observed and imaged under a fluorescence microscope (LEICA Dmi8). This evaluation was conducted with two biological replicates. Quantitative analyses of living cells were processed with ImageJ software (threshold set: 8‐255).

### Molecular Docking

Molecular Operating Environment (MOE) 2022.02 software was used to perform molecular docking study. Cryo‐EM structure of bovine MRP1 bound to leukotriene C4 (PDB ID: 5UJA), nanodisk reconstituted human ABCB1 in complex with UIC2 fab and taxol (PDB ID: 6QEX), human aminopeptidase N in complex with amastatin (PDB ID: 4FYT), and X‐ray crystallographic determination of the structure of bovine lens leucine aminopeptidase complexed with amastatin (PDB ID: 1BLL) were downloaded from https://www.rcsb.org/ as receptors. Amastatin was the reference ligand of human aminopeptidase N and bovine lens leucine aminopeptidase. All receptors were added hydrogen bonds and charges, fixed errors and missing structures, and minimized energy by executing Quickprep program. Subsequently, useless ligands and duplicate protein structures were manually removed. All ligand structures were generated by ChemDraw 19.0 software. Afterward, they were added hydrogen bonds and charges, fixed errors and missing structures by Structure Preparation program, and minimized energy in MOE. General docking type was selected. The active site of receptor was set “Ligand Atoms.” The placement was set “Triangle Matcher” and the refinement was set “Rigid Receptor.” The initial scoring methodology was set “London dG” and the final scoring methodology was set “GBVI/WSA dG.” Thirty poses would be passed to the refinement step, and 5 best poses would be finally output and analyzed. Surface maps and interaction pattern figures were presented by MOE software.

### LDH Release Assays

The seeding and starvation process were the same as cell antiproliferation evaluation. Cells were treated with 100 µL of compound solutions with different concentrations (compound 266 was tested at 12 µm) for 4 or 24 h. Cells treated with 0.5% DMSO in SFM and 1% Triton X‐100 in SFM served as blank control and positive control, respectively. Afterward, LDH working solution was prepared according to the instructions of the Cytotoxicity Detection Kit (LDH). Fifty µL of supernatant was transferred to another 96‐well plate, and 50 µL of LDH working solution was added to each well, followed by a 40 min incubation at room temperature. Subsequently, the absorbance of each well was measured by the plate reader at 490 nm.

The LDH release ratio was calculated as

(3) $$\begin{array}{ccc} \mathrm{LDH}\;\mathrm{release}\;\left(\%\right) & = & \left(OD_{\mathrm{Sample}\;\mathrm{group}}-OD_{\mathrm{Blank}}\right)\\ & & /\left(OD_{\mathrm{Positive}\;\mathrm{group}}-OD_{\mathrm{Blank}}\right)\times 100\% \end{array}$$

### Membrane Potential Assays

H460 cells were seeded in 96‐well black plates at a density of 8000 cells per well in a volume of 100 µL and cultured for 24 h. After that, cells were starved with 100 µL of SFM for a further 4–6 h. Subsequently, 75 µL of DiSC_3_(5) solution (2 µm) was added to each well, and the cells were incubated for 40 min. Baseline fluorescence was recorded for the first 10 min using a plate reader, after which 75 µL of compound solutions at their IC_50_ concentrations were added to each well. Cells treated with 0.5% DMSO in SFM served as the control. The fluorescence intensity of each well was continuously detected for other 40 min.

### SYTOX Green Assays

H460 cells were seeded in a chamber‐slide at a density of 100 000 cells per unit in a volume of 500 µL and cultured for 24 h. After that, cells were starved with 100 µL of SFM for a further 4–6 h. Three hundred µL of compound solution with the final concentration of IC_50_ was added in each unit and cells were treated for 2 h and washed with EBSS twice. Cells treated with 0.5% DMSO in SFM served as control group. Subsequently, 500 µL of solution containing 1.5 µm SYTOX Green (S7020, Thermo Fisher Scientific, Waltham, MA) was added in each well and cells were stained at room temperature for 15 min. Cells were washed with EBSS 3 times and 300 µL of EBSS was finally added in each well. The cells were imaged using a fluorescence microscope.

### Antitumor Mechanism Detection

The Muse Cell Analyzer (Merck, USA), which operated based on flow cytometry, was used for this analysis. H460 cells were seeded in 24‐well plate at a density of 200 000 cells per well in a volume of 100 µL and cultured for 24 h. After that, cells were starved with 100 µL of SFM for a further 4–6 h. Three hundred µL of compound solutions at different concentrations were added in each well, and cells were treated for different time points. Cells treated with 0.5% DMSO in SFM served as control group. Afterward, cells in each well were washed with 100 µL of EBSS and detached from the plate by incubating with 100 µL of trypsin–EDTA (0.05%) solution. Subsequently, 300 µL of CGM was added in each well to inactivate the trypsin. All the abovementioned solutions and medium were collected in 1.5 mL tubes. An additional 100 µL of EBSS was used to wash the well again and transferred in the tubes. All 1.5 mL tubes were centrifuged at 400 × *g* for 10 min. Subsequently, the supernatant of each tube was discarded, and the cell pellet was resuspended in 50 µL of EBSS. Cell suspensions were then mixed with the appropriate kit working solutions according to the manufacturer's instructions. After mixing and incubation, the samples were analyzed using the cell analyzer. Muse Annexin V and Dead Cell Kit, Muse Cell Cycle Kit, Muse Caspase‐3/7 Kit, Muse MitoPotential Kit, and Muse Oxidative Stress Kit were used.

### Cell Binding Assays

For the quantitative binding assay, tumor cells and normal cells were seeded in 96‐well black plates at a density of 8000 cells per well in a volume of 100 µL and cultured for 24 h. Afterward, cells were treated by 100 µL of FITC‐label compound solutions with different concentrations (compound 275 was tested at 12 µm) for 2 h and washed by phosphate‐buffered saline (PBS) 3 times. Cells treated with 0.5% DMSO in SFM were used as blank group. Finally, 100 µL of PBS was added to each well, and the fluorescence intensity was measured with an excitation wavelength of 490 nm and an emission wavelength of 525 nm. For the visualization assay, tumor and normal cells were seeded in chamber slides at a density of 100 000 cells per chamber in 500 µL of medium and cultured for 24 h. Afterward, cells in each well were treated by 300 µL of FITC‐label compound solutions with the final concentration of IC_50_ (compound 275 was tested at 12 µm) for 2 h and washed by EBSS 3 times. Cells treated with 0.5% DMSO in SFM served as the blank group. Subsequently, cells were fixed with 4% paraformaldehyde in PBS v/v at room temperature for 10 min. Afterward, cells were washed once and stained with 500 µL of mix solution containing 4 µg mL^−1^ DAPI and 2 µg mL^−1^ FM4‐64 at room temperature for 10 min and washed with EBSS 3 times. Finally, the cells were imaged using a fluorescence microscope.

### Competitive Binding Assays

NCI‐H460 and NCI‐H838 cells were seeded in 96‐well black plates at a density of 8000 cells per well in a volume of 100 µL and cultured for 24 h. Afterward, cells were treated with 75 µL of piperine or probenecid solutions with concentrations of 1, 0.75, 0.5, 0.25, and 0.1 mm for 2 h. Subsequently, 75 µL of compound 174‐3 solutions at their IC_50_ concentrations (specifically determined for H460 or H838 cells) was added to each well, followed by a 2 h incubation. Cells treated with SFM and only with compound 174‐3 solutions served as blank control and positive control, respectively. Finally, cells were washed by PBS 3 times, and 100 µL of PBS was added to each well. The fluorescence intensity was measured with an excitation wavelength of 490 nm and an emission wavelength of 525 nm.

The binding ratio was calculated as

(4) $$\begin{array}{ccc} \mathrm{Binding}\;\left(\%\right) & = & \left(OD_{\mathrm{Sample}\;\mathrm{group}}-OD_{\mathrm{Blank}\;\mathrm{group}}\right)\\ & & /\left(OD_{\mathrm{Positive}\;\mathrm{group}}-OD_{\mathrm{Blank}\mathrm{group}}\right)\times 100\% \end{array}$$

### TNF‐α Assays

RAW264.7 murine macrophages were seeded in 6‐well plates at the density of 500 000 cells per well in a volume of 1 mL and incubated for 24 h. Cells were then treated with SFM (negative group), 10 ng mL^−1^ LPS (positive group), or 50 µm of compound 174‐3 for an additional 24 h. Following incubation, culture supernatants were collected, and TNF‐α levels were measured using human TNF‐α ELISA Kit (ThermoFisher, Waltham, MA, USA), according to the instructions of the manufacturer.

### Secondary Structure Prediction and Conformation

The secondary structures of two linkers (GPLGLAG and GGGGGGG) were predicted by AlphaFold2 (https://colab.research.google.com/github/sokrypton/ColabFold/blob/main/AlphaFold2.ipynb#scrollTo = G4yBrceuFbf3) and displayed by PyMOL software. The secondary structures of conjugate 209 and conjugate 288 were investigated with a JASCO‐815 CD spectrometer (Jasco, Essex, UK). Compound stock solutions were diluted with ddH_2_O or SDS (30 mm) solution at a final concentration of 50 µm. The wavelength range of CD spectrum was from 190 to 240 nm and the temperature was 298 K.

### MMP Cleavage Detection

NCI‐H460 cells were seeded in 48‐well plates at a density of 24 000 cells per well in a volume of 300 µL and cultured for 24 h. Afterward, 300 µL of SFM was added to each well followed by a 10 h incubation. Next, the supernatant of each well was transferred to 1.5 mL tubes. The supernatant of control group was incubated at 60 °C for 30 min (control medium) while the supernatant of dosing group (condition medium) had no incubation. Subsequently, stock solutions of conjugates 209 and 288 were added in the supernatant with the final concentration of 50 µm followed by another 10 h incubation. Last, 150 µL of samples were analyzed by analytical HPLC and ESI‐MS. HPLC condition: gradient: 100% solution A from 0 to 5 min and 100% solution A to 0% solution A from 5 to 65 min; flow rate: 1 mL min^−1^; detector wavelength: 214 nm. Each sample had two replicates.

### Antiproliferative Activity of Conjugate 209 with MMP Inhibitor against NCI‐H460

NCI‐H460 cells were seeded in 96‐well plates at a density of 8000 cells per well in a volume of 100 µL and cultured for 24 h. Afterward, cells were treated with 75 µL of MMP‐2/MMP‐9 Inhibitor IV solutions at concentrations of 100, 75, 50, 25, and 0 µm for 4 h. Subsequently, another 75 µL of conjugate 209 solution was added in each well to make the final concentration of 50 µm followed by a 24 h incubation. Cells treated with 0.5% DMSO in SFM served as blank growth control. The following procedures were the same as cell antiproliferation evaluation.

### Liposome Leakage Assays

The lipid mixture (4:1 POPC:cholesterol) was prepared using 1‐palmitoyl‐2‐oleoyl‐glycero‐3‐phosphocholine (POPC, Avanti) and cholesterol (Sigma). A 10 mg lipid mixture was dissolved in chloroform, evaporated at 40 °C for 30 min under a stream of nitrogen, and vacuum‐dried for 4 h to remove residual solvents. The lipid film was hydrated with Tris buffer (10 mm Tris, 150 mm NaCl, 1 mm EDTA, pH 7.4) containing 70 mm calcein, subjected to five freeze–thaw cycles, and extruded 13 times through a 100 nm membrane (Avanti Polar Lipids, USA) to form unilamellar vesicles. Nonencapsulated calcein was removed using a PD10 desalting column (Sephadex G‐25M, GE Healthcare) pre‐equilibrated with Tris buffer. Fractions were pooled based on calcein fluorescence (excitation 490 nm, emission 520 nm), and the final suspension was adjusted to 1 mg mL^−1^ lipid concentration. Calcein release was monitored fluorometrically (Synergy HT, BioTek, USA), with the maximum fluorescence (100% leakage) measured after adding 1% Triton X‐100.

The leakage ratio was calculated as

(5) $$\begin{array}{ccc} \mathrm{Leakage}\left(\%\right) & = & \left(OD_{\mathrm{Sample}\;\mathrm{group}}-OD_{\mathrm{Blank}\;\mathrm{group}}\right)\\ & & /\left(OD_{\mathrm{Positive}\;\mathrm{group}}-OD_{\mathrm{Blank}\mathrm{group}}\right)\times 100\% \end{array}$$

### Antiproliferative Activity Stability Evaluation

The antimicrobial stability evaluation was determined in the presence of physiological concentrations of serum. Twenty five µL of compound solutions with the concentration of 3 mm and 25 µL of human serum were mixed. The mixture was incubated for 0, 2, 4, 6, 12, 24 h at 37 °C and then incubated for 15 min at 60 °C to inactivate the enzymes. Afterward, the mixture was diluted by SFM to prepare dosing solutions with concentrations from 100 to 1.5625 µm with twofold dilution. The antiproliferative activity evaluation procedure was the same as before mentioned.

### HSA Quenching Assays

HSA (20 µm) and different concentrations of compounds were coincubated at 25 or 37 °C for 15 min. Afterward, fluorescence emission spectra of each sample were detected with plate reader (BioTek Synergy H1, Agilent, USA). The excitation wavelength was 285 nm, and the emission wavelength range was from 320 to 350 nm. The emission step was 2. The optics position was top and the gain was 85. The assays were conducted with two biological replicates. The *K*_SV_ and *k*_q_ values were calculated by employing the Stern–Volmer equation

(6) F0/F=1+Ksv×Q=1+kq·δ0×Q

*F*0 and *F* represent the fluorescence intensity values without and with a quencher, respectively. Without a quencher, *δ*0 represents a molecular fluorescence lifetime of 10^−8^ s, and *Q* is the quencher concentration.

### Compounds Remaining Evaluation and Degradation Products Analysis in Human Serum

Compound solutions (50 µL, 3 mm) were mixed with human serum (50 µL) and incubated for 0, 2, 4, 6, 12, and 24 h at 37 °C. Ethanol (250 µL) was added to precipitate serum proteins, and the samples were centrifuged at 14 000 × *g* for 20 min at 4 °C. Subsequently, 150 µL of supernatant of each sample was analyzed with analytical HPLC and ESI‐MS/MS. HPLC condition: gradient: 100% solution A to 0% solution A from 0 to 60 min; flow rate: 1 mL min^−1^; detector wavelength: 214 nm. Each sample had two replicates.

The compound remaining ratio was calculated as

(7) $$\begin{array}{ccc} \mathrm{Compound}\;\mathrm{remaining}\left(\%\right) & = & \mathrm{Compound}\;\mathrm{Peak}\;\mathrm{Are}a_{\mathrm{not}\;\mathrm{at}\;0\;h}\\ & & /\;\mathrm{Compound}\;\mathrm{Peak}\;\mathrm{Are}a_{\mathrm{at}\;0\;h}\times 100\% \end{array}$$

### In Vivo Safety Evaluation

*G. mellonella* larvae (Livefoods Direct Ltd., Yorkshire, UK) were used to assess the in vivo safety of peptides. Experiments were conducted on larvae (250 ± 25 mg) incubated at 37 °C. Ten µL of compound 270 solutions with the final concentrations of 50, 80, and 120 µm were injected into the hemolymph via the second pair of prolegs (counting from the head). Control groups were injected with an equal volume of PBS. Larval survival and body weight were monitored every 1 day for a total of 5 days.

### Chemotherapeutics‐Resistant NCI‐H460 Cell Line Establishment

Wild type H460 cells were cultured in a T‐75 flask and exposed to 30 µm of cisplatin. Cells were passed when the confluency reached 90%. The IC_50_ value of cisplatin on this cell line was evaluated every two passages. After the IC_50_ value was higher than 500 µm, the cultured medium was changed to normal CGM, and the cell line was cultured for another three passages. The IC_50_ value was evaluated again to make sure the resistance was stable. The other three resistance cell lines establishment process was similar. Other resistant cell lines were established in a similar manner. Parental NCI‐H460 cells were exposed to 10 µm 5‐fluorouracil, 2.5 µm paclitaxel, or 2.5 µm etoposide to generate 5‐fluorouracil‐resistant, paclitaxel‐resistant, and etoposide‐resistant cell lines, respectively.

### Statistical Analysis

All assays without further specification were performed with three biological replicates and three technical replicates each. Data represented the mean ± SEM, and *n* values were indicated in the figure legends where applicable. GraphPad Prism 8.0.2 software (San Diego, USA) was used for all statistical analyses. No data transformation or outlier exclusion was applied. One‐way ANOVA with Dunnett's post hoc test or unpaired two‐tailed *t*‐tests was used to compare treatment groups with controls. All tests were two‐sided, with a significance level of *α* = 0.05. Statistical significance was defined as follows: ns for *p* > 0.05, * for *p* < 0.05, ** for *p* < 0.01, *** for *p* < 0.001, and **** for *p* < 0.0001.

## Conflict of Interest

The authors declare no conflict of interest.

## Supporting information



Supporting Information

## Data Availability

The data that support the findings of this study are available from the corresponding author upon reasonable request.
