# Supplementary material for: A Serum‐Stable Antimicrobial Peptide‐Based Delivery Platform for Selective Treatment of Nontargetable and Chemoresistant Tumors
Source: Adv Sci (Weinh). 2025 Sep 17;12(45):e04710. doi: 10.1002/advs.202504710 (PMC12677599; doi:10.1002/advs.202504710)
Supplement: Supplementary file 1 — Supporting Information [file ADVS-12-e04710-s001.docx]

A Serum-Stable Antimicrobial Peptide-Based Delivery Platform for Selective Treatment of Non-Targetable and Chemo-Resistant Tumors

Table of Contents

[Compound Synthesis and Characterization 4](#_Toc203987826)

[Compound 111 4](#_Toc203987827)

[Compound 266 5](#_Toc203987828)

[Compound 174-3 6](#_Toc203987829)

[Compound 228 7](#_Toc203987830)

[Conjugate 204 8](#_Toc203987831)

[Conjugate 207 9](#_Toc203987832)

[Conjugate 209 10](#_Toc203987833)

[Conjugate 288 11](#_Toc203987834)

[Compound 239-1 12](#_Toc203987835)

[Compound 239-2 13](#_Toc203987836)

[Compound 239-3 14](#_Toc203987837)

[Compound 239-4 15](#_Toc203987838)

[Compound 239-4-Ac 16](#_Toc203987839)

[Conjugate 247 17](#_Toc203987840)

[Conjugate 270 18](#_Toc203987841)

[Compound 273 19](#_Toc203987842)

[Compound 275 20](#_Toc203987843)

[Compound 277 21](#_Toc203987844)

[Table S1. 22](#_Toc203987845)

[Table S2. 23](#_Toc203987846)

[Figure S37. 24](#_Toc203987847)

[Figure S38. 25](#_Toc203987848)

[Figure S39. 27](#_Toc203987849)

[Figure S40. 28](#_Toc203987850)

[Figure S41. 28](#_Toc203987851)

[Table S3. 29](#_Toc203987852)

[Figure S42. 29](#_Toc203987853)

[Figure S43. 30](#_Toc203987854)

[Figure S44. 30](#_Toc203987855)

[Table S4. 31](#_Toc203987856)

[Figure S45. 31](#_Toc203987857)

[Figure S46. 32](#_Toc203987858)

[Figure S47. 33](#_Toc203987859)

[Figure S48. 34](#_Toc203987860)

[Figure S49. 34](#_Toc203987861)

[Table S5. 35](#_Toc203987862)

[Figure S50. 35](#_Toc203987863)

[Figure S51. 36](#_Toc203987864)

[Figure S52. 37](#_Toc203987865)

[Table S6. 38](#_Toc203987866)

[Figure S53. 38](#_Toc203987867)

[Figure S54. 39](#_Toc203987868)

[Figure S55. 40](#_Toc203987869)

[Table S7. 41](#_Toc203987870)

[Figure S56. 41](#_Toc203987871)

[Figure S57. 42](#_Toc203987872)

[Figure S58. 43](#_Toc203987873)

[Table S8. 44](#_Toc203987874)

[Figure S59. 44](#_Toc203987875)

[Figure S60. 45](#_Toc203987876)

[Figure S61. 46](#_Toc203987877)

[Figure S62. 46](#_Toc203987878)

[Table S9. 47](#_Toc203987879)

[Figure S63. 47](#_Toc203987880)

[Table S10. 48](#_Toc203987881)

[Figure S64. 48](#_Toc203987882)

[Figure S65. 50](#_Toc203987883)

[Figure S66. 51](#_Toc203987884)

[Figure S67. 51](#_Toc203987885)

[Figure S68. 53](#_Toc203987886)

[Table S11. 54](#_Toc203987887)

[Figure S69. 55](#_Toc203987888)

## Compound Synthesis and Characterization

### Compound 111

Sequence: FFPLIFGALSSILPKIL-NH_2_

After swelling the resin and removing the Fmoc protecting group, all amino acids were sequentially conjugated to the resin in a stepwise manner, following the peptide sequence from C- to N-terminus. Finally, the resin was cleaved, and the crude product was obtained.

HPLC purity: 98.21%

Electrospray-ionization mass spectrometry (ESI-MS): calculated for C_96_H_151_N_19_O_19_ [M+H]^+^: 1876.38, [M+2H]^2+^: 938.69, [M+3H]^3+^: 626.13; found for: [M+H]^+^: 1876.59, [M+2H]^2+^: 938.89, [M+3H]^3+^: 626.41.


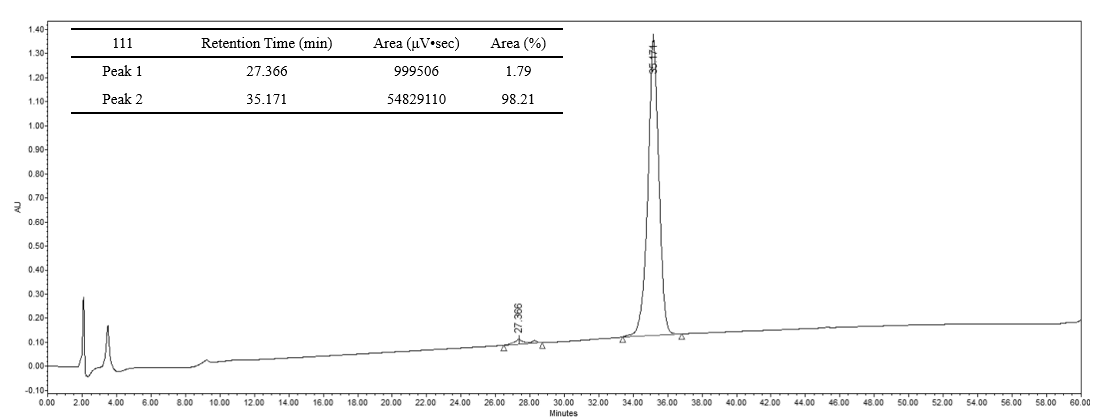


Figure S1. Analytical HPLC chromatogram of compound 111.

Figure S2. ESI-MS spectrum of compound 111.

### Compound 266

Sequence: FFPKIFDDLSSILPKIL-NH_2_

Compound 266 was synthesized according to the synthesis process of compound 111.

HPLC purity: 98.13%

ESI-MS: calculated for C_99_H_154_N_20_O_23_ [M+2H]^2+^: 997.22, [M+3H]^3+^: 665.15; found for: [M+2H]^2+^: 997.30, [M+3H]^3+^: 665.31.


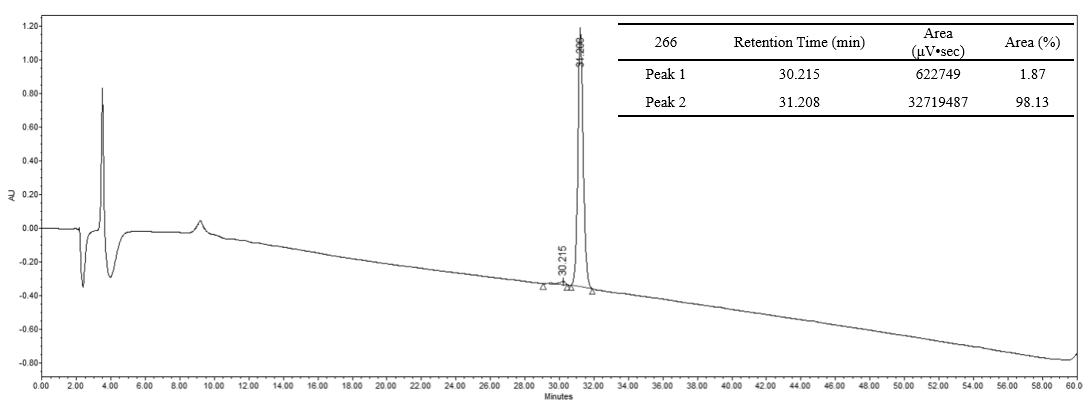


Figure S3. Analytical HPLC chromatogram of compound 266.

Figure S4. ESI-MS spectrum of compound 266.

### Compound 174-3

Sequence: FFPKIFDDLE(PIP)SILPKIL-NH_2_

After the SILPKIL peptide was attached to the resin and the Fmoc group of the Ser was deprotected, the Fmoc-Glu(OAll) was coupled to the resin and the OAll group was deprotected. Subsequently, the PIP group was conjugated to the side-chain carboxyl group of Glu. Then the Fmoc group was deprotected and the remaining amino acids were attached to the resin according to the sequence. The crude product was obtained after the final Fmoc group deprotection and resin cleavage.

HPLC purity: > 99%

ESI-MS: calculated for C_106_H_165_N_21_O_23_ [M+2H]^2+^: 1051.81, [M+3H]^3+^: 701.54; found for: [M+2H]^2+^: 1051.83, [M+3H]^3+^: 701.76.


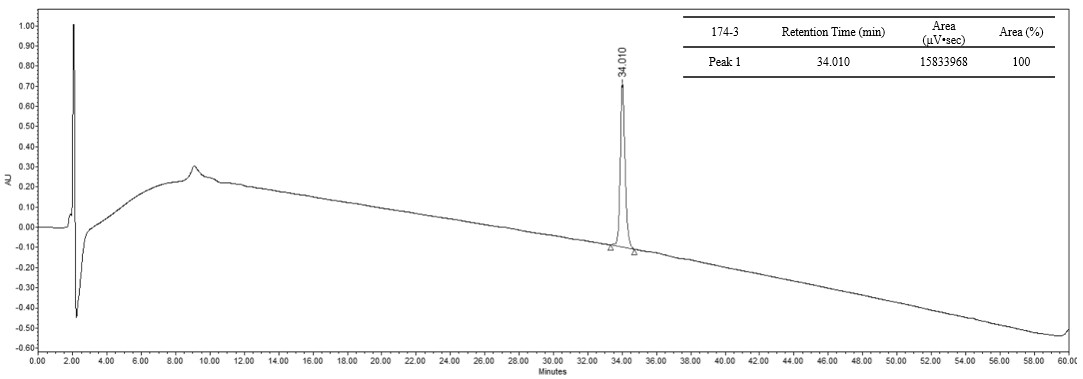


Figure S5. Analytical HPLC chromatogram of compound 174-3.

Figure S6. ESI-MS spectrum of compound 174-3.

### Compound 228

Sequence: FFPKIFDDLFSILPKIL-NH_2_

Compound 228 was synthesized according to the synthesis process of compound 111.

HPLC purity: > 99%

ESI-MS: calculated for C_105_H_158_N_20_O_22_ [M+2H]^2+^: 1027.12, [M+3H]^3+^: 685.08; found for: [M+2H]^2+^: 1027.23, [M+3H]^3+^: 685.19.


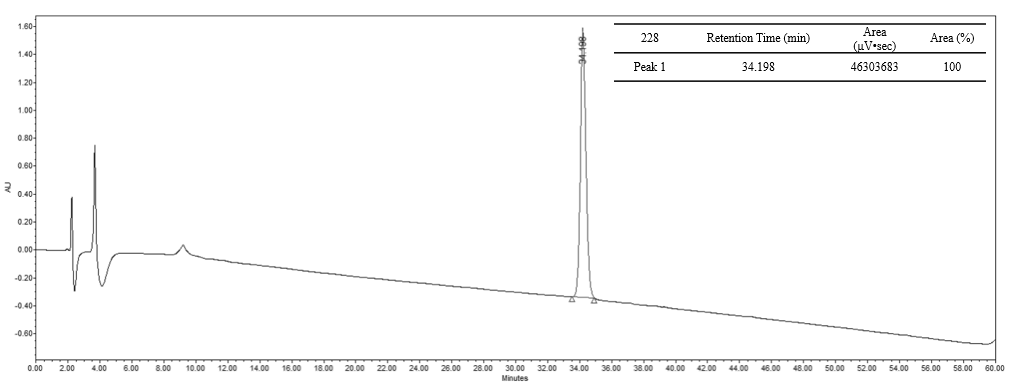


Figure S7. Analytical HPLC chromatogram of compound 228.

Figure S8. ESI-MS spectrum of compound 228.

### Conjugate 204

Sequence: FFPKIFDDLE(PIP)SILPKILGPLGLAG-PEG_4_-NH_2_

After Fmoc-PEG_4_-OH was attached to the resin as the C-terminal end of the peptide, the linker sequence GPLGLAG was added. The remaining portion of conjugate 204 was then synthesized using the same process as compound 174-3.

HPLC purity: 98.07%

ESI-MS: calculated for C_143_H_229_N_29_O_35_ [M+2H]^2+^: 1458.29, [M+3H]^3+^: 972.52; found for: [M+2H]^2+^: 1458.36, [M+3H]^3+^: 972.36.


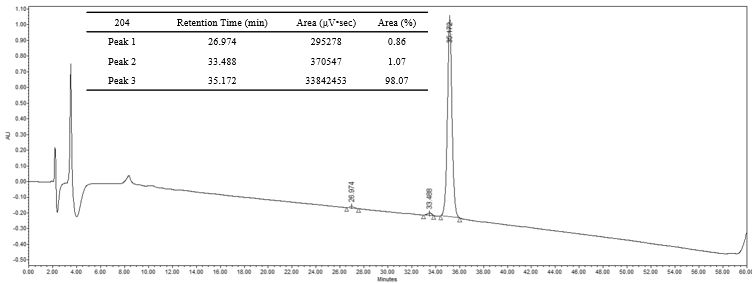


Figure S9. Analytical HPLC chromatogram of conjugate 204.

Figure S10. ESI-MS spectrum of conjugate 204.

### Conjugate 207

Sequence: FFPKIFDDLE(PIP)SILPKILGPLGLAG-(PEG_4_)_2_-NH_2_

Conjugate 207 was synthesized according to the synthesis process of conjugate 204.

HPLC purity: 99.00%

ESI-MS: calculated for C_154_H_250_N_30_O_40_ [M+2H]^2+^: 1581.93, [M+3H]^3+^: 1054.95, [M+4H]^4+^: 791.47, [M+5H]^5+^: 633.37; found for: [M+2H]^2+^: 1582.41, [M+3H]^3+^: 1054.76, [M+4H]^4+^: 791.54, [M+5H]^5+^: 633.45.


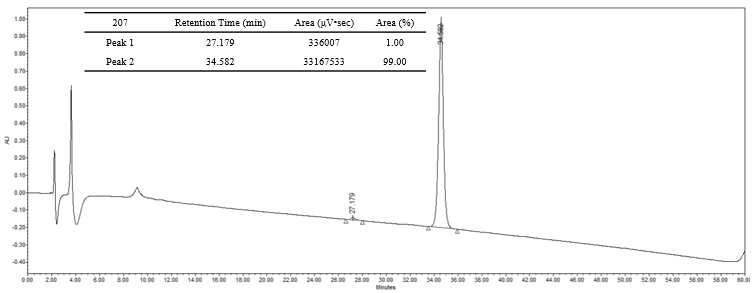


Figure S11. Analytical HPLC chromatogram of conjugate 207.

Figure S12. ESI-MS spectrum of conjugate 207.

### Conjugate 209

Sequence: FFPKIFDDLE(PIP)SILPKILGPLGLAG-(PEG_4_)_4_-NH_2_

Conjugate 209 was synthesized according to the synthesis process of conjugate 204.

HPLC purity: > 99%

ESI-MS: calculated for C_154_H_250_N_30_O_40_ [M+3H]^3+^: 1219.82, [M+4H]^4+^: 915.11; found for: [M+3H]^3+^: 1219.64, [M+4H]^4+^: 914.92.


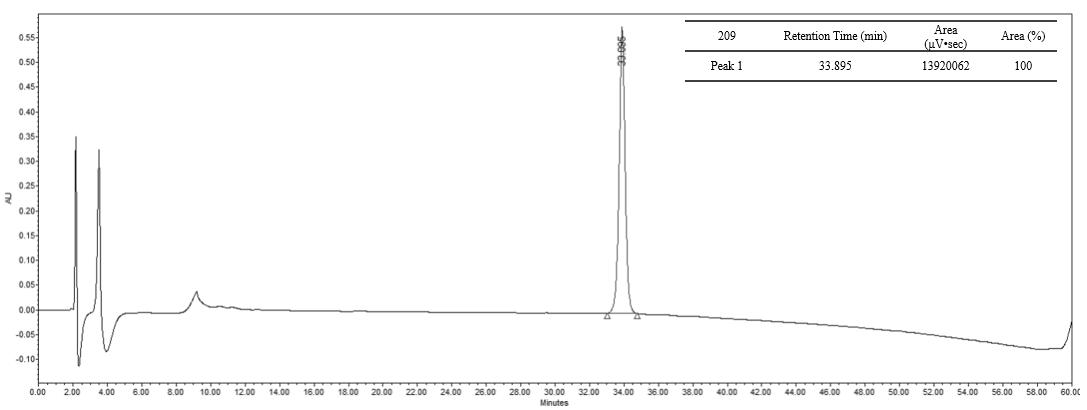


Figure S13. Analytical HPLC chromatogram of conjugate 209.

Figure S14. ESI-MS spectrum of conjugate 209.

### Conjugate 288

Sequence: FFPKIFDDLE(PIP)SILPKILGGGGGGG-(PEG_4_)_4_-NH_2_

Conjugate 288 was synthesized according to the synthesis process of conjugate 204.

HPLC purity: 99.35%

ESI-MS: calculated for C_154_H_250_N_30_O_40_ [M+2H]^2+^: 1746.07, [M+3H]^3+^: 1164.38, [M+4H]^4+^: 873.54, [M+5H]^5+^: 699.03, [M+6H]^6+^: 582.69; found for: [M+2H]^2+^: 1746.29, [M+3H]^3+^: 1164.12, [M+4H]^4+^: 873.46, [M+5H]^5+^: 698.93, [M+6H]^6+^: 582.68.


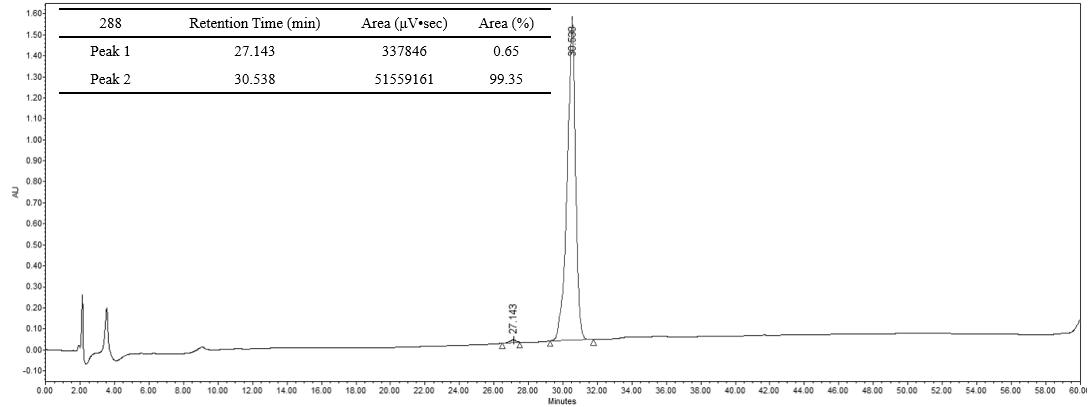


Figure S15. Analytical HPLC chromatogram of conjugate 288.

Figure S16. ESI-MS spectrum of conjugate 288.

### Compound 239-1

Sequence: AEFFPKIFDDLE(PIP)SILPKIL-NH_2_

Compound 239-1 was synthesized according to the synthesis process of compound 174-3.

HPLC purity: 97.81%

ESI-MS: calculated for C_114_H_177_N_23_O_27_ [M+2H]^2+^: 1151.90, [M+3H]^3+^: 768.27; found for: [M+2H]^2+^: 1152.39, [M+3H]^3+^: 768.73.


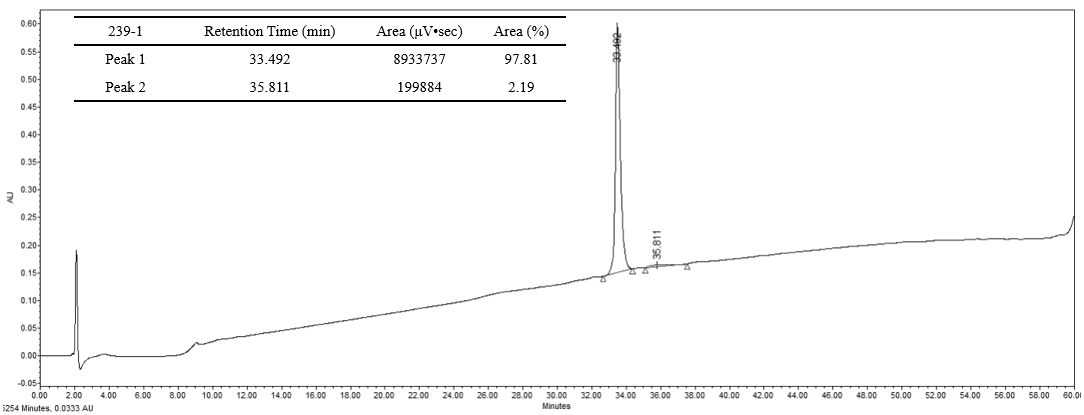


Figure S17. Analytical HPLC chromatogram of compound 239-1.

Figure S18. ESI-MS spectrum of compound 239-1.

### Compound 239-2

Sequence: ATFFPKIFDDLE(PIP)SILPKIL-NH_2_

Compound 239-2 was synthesized according to the synthesis process of compound 174-3.

HPLC purity: > 99%

ESI-MS: calculated for C_113_H_177_N_23_O_26_ [M+2H]^2+^: 1137.90, [M+3H]^3+^: 758.93; found for: [M+2H]^2+^: 1137.79, [M+3H]^3+^: 758.94.


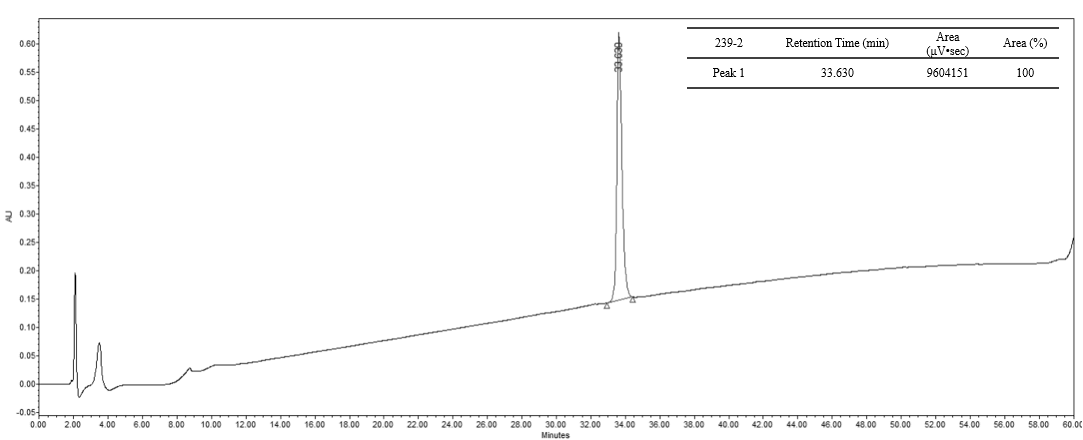


Figure S19. Analytical HPLC chromatogram of compound 239-2.

Figure S20. ESI-MS spectrum of compound 239-2.

### Compound 239-3

Sequence: ARFFPKIFDDLE(PIP)SILPKIL-NH_2_

Compound 239-3 was synthesized according to the synthesis process of compound 174-3.

HPLC purity: 98.70%

ESI-MS: calculated for C_115_H_182_N_26_O_25_ [M+2H]^2+^: 1165.44, [M+3H]^3+^: 777.29; found for: [M+2H]^2+^: 1165.38, [M+3H]^3+^: 777.26.


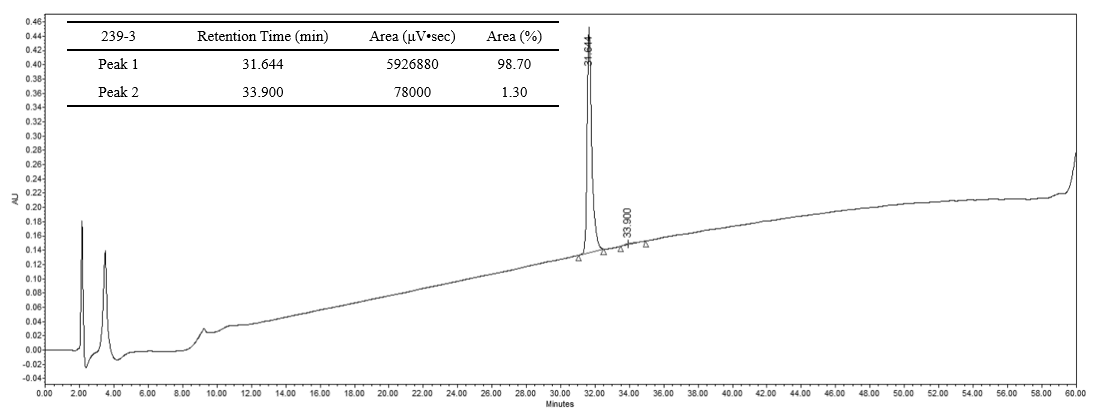


Figure S21. Analytical HPLC chromatogram of compound 239-3.

Figure S22. ESI-MS spectrum of compound 239-3.

### Compound 239-4

Sequence: AKFFPKIFDDLE(PIP)SILPKIL-NH_2_

Compound 239-4 was synthesized according to the synthesis process of compound 174-3.

HPLC purity: 96.67%

ESI-MS: calculated for C_115_H_182_N_24_O_25_ [M+2H]^2+^: 1151.43, [M+3H]^3+^: 767.95; found for: [M+2H]^2+^: 1151.33, [M+3H]^3+^: 767.93.


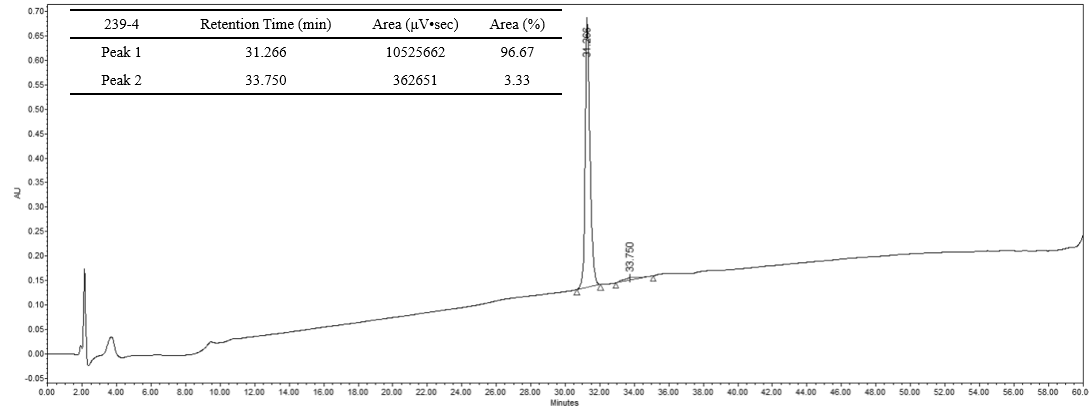


Figure S23. Analytical HPLC chromatogram of compound 239-4.

Figure S24. ESI-MS spectrum of compound 239-4.

### Compound 239-4-Ac

Sequence: AK(Ac)FFPKIFDDLE(PIP)SILPKIL-NH_2_

After compound 174-3 were coupled to the resin and the Fmoc group of the first Phe at N-terminus was deprotected, Fmoc-Lys (Mtt)-OH was attached to the resin, and the Mtt group was removed. Subsequently, the side-chain amino group of the Lys was acetylated and then the Fmoc at the main chain was removed. Following that, the last Ala was coupled. The crude product was obtained after the final Fmoc group deprotection and resin cleavage.

HPLC purity: 98.72%

ESI-MS: calculated for C_117_H_184_N_24_O_26_ [M+2H]^2+^: 1172.45, [M+3H]^3+^: 781.97; found for: [M+2H]^2+^: 1172.25, [M+3H]^3+^: 781.82.


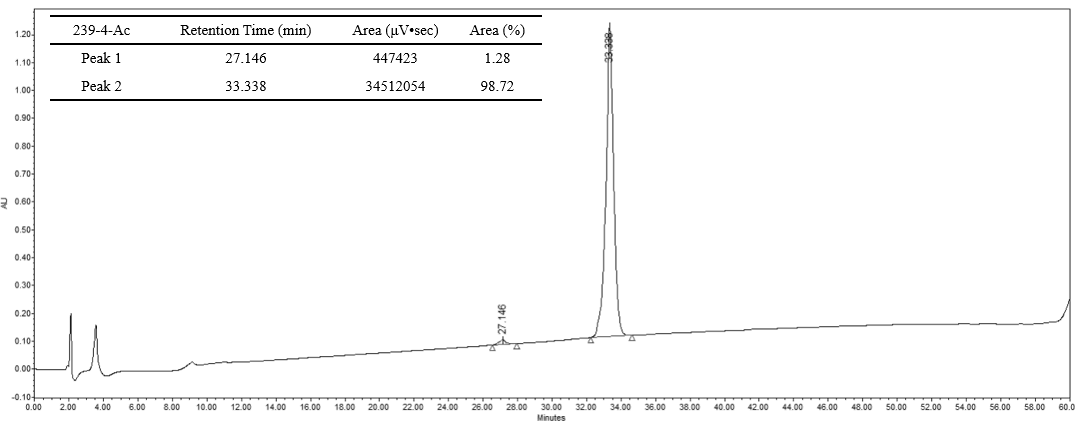


Figure S25. Analytical HPLC chromatogram of compound 239-4-Ac.

Figure S26. ESI-MS spectrum of compound 239-4-Ac.

### Conjugate 247

Sequence: AKFFPKIFDDLE(PIP)SILPKILGPLGLAG-(PEG_4_)_4_-NH_2_

Conjugate 247 was synthesized according to the synthesis process of conjugate 204.

HPLC purity: 98.96%

ESI-MS: calculated for C_185_H_309_N_35_O_52_ [M+2H]^2+^: 1928.85, [M+3H]^3+^: 1286.23, [M+4H]^4+^: 964.93, [M+5H]^5+^: 772.14, [M+6H]^6+^: 643.62; found for: [M+2H]^2+^: 1928.60, [M+3H]^3+^: 1285.91, [M+4H]^4+^: 964.90, [M+5H]^5+^: 771.97, [M+6H]^6+^: 643.50.


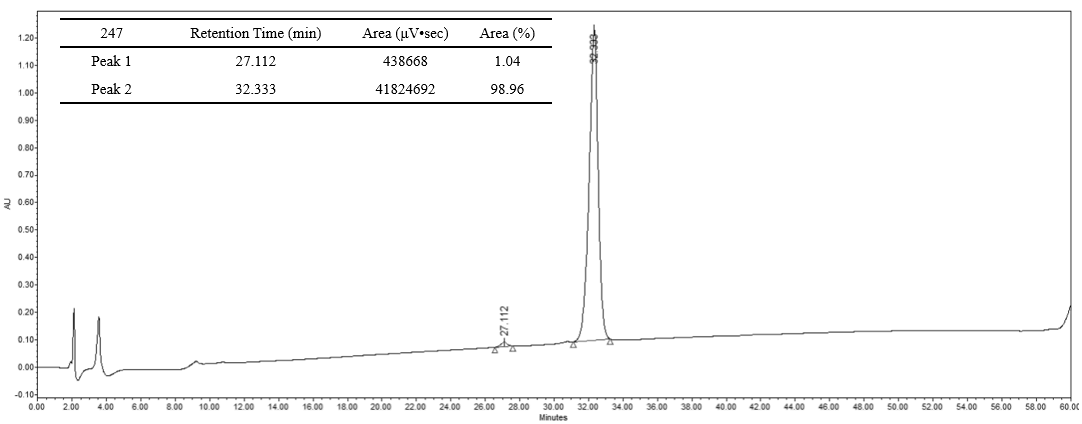


Figure S27. Analytical HPLC chromatogram of conjugate 247.

Figure S28. ESI-MS spectrum of conjugate 247.

### Conjugate 270

Sequence: AKFFPKIFDDLE(PIP)SILPKILGPLGLAG-(PEG_4_)_6_-NH_2_

Conjugate 270 was synthesized according to the synthesis process of conjugate 204.

HPLC purity: > 99%

ESI-MS: calculated for C_207_H_351_N_37_O_62_ [M+3H]^3+^: 1451.09, [M+4H]^4+^: 1088.57, [M+5H]^5+^: 871.06, [M+6H]^6+^: 726.05; found for: [M+3H]^3+^: 1451.02, [M+4H]^4+^:1088.50, [M+5H]^5+^: 870.97, [M+6H]^6+^: 726.03.


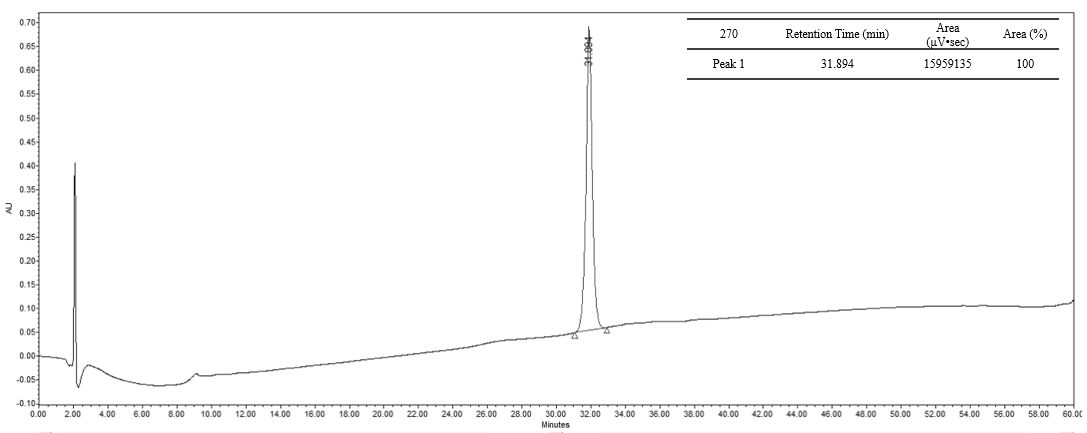


Figure S29. Analytical HPLC chromatogram of conjugate 270.

Figure S30. ESI-MS spectrum of conjugate 270.

### Compound 273

Sequence: FFPLIFGALSSILPKILK(FITC)-NH_2_

After the first Fmoc-Lys (Mtt)-OH was coupled to the resin, other amino acids were coupled to the resin sequentially without deprotecting the Fmoc group of the N-terminal Phe. The Mtt group was removed, and FITC group was conjugated to the side-chain amino group of the Lys at the C-terminus. The crude product was obtained after the final Fmoc group deprotection and resin cleavage.

HPLC purity: 99.71%

ESI-MS: calculated for C_123_H_174_N_22_O_25_S [M+2H]^2+^: 1197.47, [M+3H]^3+^: 798.64; found for: [M+2H]^2+^: 1197.64, [M+3H]^3+^: 798.87.


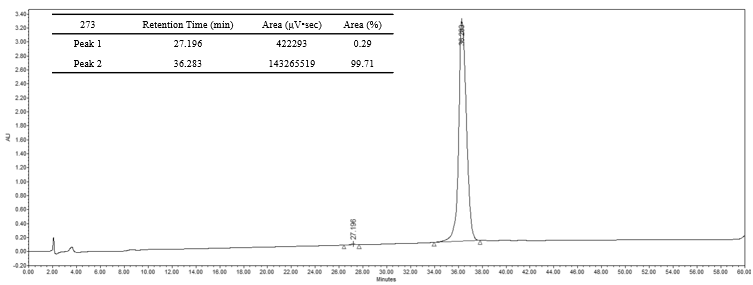


Figure S31. Analytical HPLC chromatogram of compound 273.

Figure S32. ESI-MS spectrum of compound 273.

### Compound 275

Sequence: FFPKIFDDLSSILPKILK(FITC)-NH_2_

Compound 275 was synthesized according to the synthesis process of compound 273.

HPLC purity: 99.27%

ESI-MS: calculated for C_126_H_177_N_23_O_29_S [M+2H]^2+^: 1256.00, [M+3H]^3+^: 837.66, [M+4H]^4+^: 628.50; found for: [M+2H]^2+^: 1256.12, [M+3H]^3+^: 838.00. [M+4H]^4+^: 628.85.


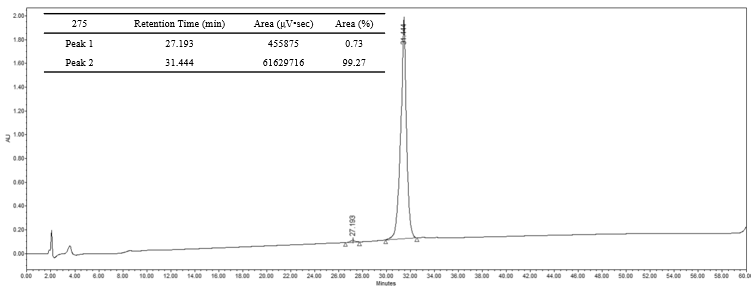


Figure S33. Analytical HPLC chromatogram of compound 275.

Figure S34. ESI-MS spectrum of compound 275.

### Compound 277

Sequence: FFPKIFDDLE(PIP)SILPKILK(FITC)-NH_2_

Compound 275 was synthesized according to the synthesis process of compound 273 and compound 174-3.

HPLC purity: > 99%

ESI-MS: calculated for C_133_H_188_N_24_O_29_S [M+2H]^2+^: 1310.59, [M+3H]^3+^: 874.06, [M+4H]^4+^: 655.79; found for: [M+2H]^2+^: 1311.26, [M+3H]^3+^:874.29. [M+4H]^4+^: 656.09.


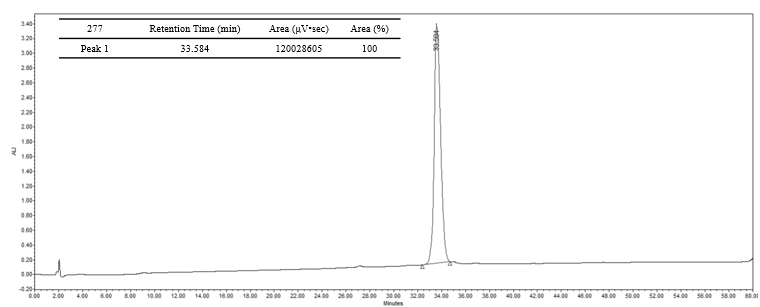


Figure S35. Analytical HPLC chromatogram of compound 277.

Figure S36. ESI-MS spectrum of compound 277.

Table S1. IC_50_ and HC_10_ values of all peptides.

|  | **​** | 111 | 266 | 174-3 | 228 | 204 | 207 | 209 | 288 | 239-1 | 239-2 | 239-3 | 239-4 | 247 | 270 |
| --- | --- | --- | --- | --- | --- | --- | --- | --- | --- | --- | --- | --- | --- | --- | --- |
| Cancer Cell Line | H460 | 6.03±0.18 | >100 | 23.65±1.03 | 17.51±0.91 | 19.96±1.05 | 28.03±1.59 | 53.37±2.81 | >100 | 88.23 | 36.83±3.25 | 14.69±1.22 | 22.22±1.06 | 29.20±1.33 | 53.63±2.10 |
|  | H838 | 10.95±1.06 | >100 | 39.54±2.24 | 26.41±1.78 |  |  | 84.50±5.91 |  |  |  |  |  |  |  |
|  | HT-1080 | 7.37±0.70 | >100 | 57.64±8.46 | 30.52±6.05 |  |  | 86.78±49.1 |  |  |  |  |  |  |  |
| Normal Cell Line | HaCaT | 13.57±0.91 | >100 | 87.05 | 34.03±2.66 |  |  | >100 |  |  |  |  |  | >100 | >100 |
|  | HEK-293 | 8.03±1.10 | >100 | 32.63±1.55 | 13.84±1.42 | 34.10±3.41 | 44.18±3.21 | 98.6 | >100 | >100 | 44.79±4.06 | 15.01±1.38 | 31.48±4.28 | 51.86±5.64 | >100 |
|  | HMEC-1 | 13.45±0.80 | >100 | 52.85±7.31 | 30.70±3.14 |  |  | >100 |  |  |  |  |  | >100 | >100 |
|  | MRC-5 | 26.28±2.59 | >100 | 95.76 | 34.14±2.32 |  |  | >100 |  |  |  |  |  | >100 | >100 |
|  | HC_10_^a^ | 6.59 | >100 | 51.52 | 14.57 | 46.10 | 69.26 | >100 | >100 | 97.64 | 57.04 | 24.72 | 25.61 | >100 | >100 |

^a^: HC_10_ value stands for the concentration causing 10% of erythrocytes lysis.

Table S2. IC_50_ values of peptides 174-3, 209, 239-1, 239-2, 239-3, 239-4 and 270 untreated or pre-incubated with 50% human serum for different time against H460 cell line.

| **​** | IC_50_ Value (µM)​ | | | | | | |
| --- | --- | --- | --- | --- | --- | --- | --- |
|  | 174-3 | 209 | 239-1 | 239-2 | 239-3 | 239-4 | 270 |
| without serum | 23.65±1.03​ | 53.37±2.81​ | 88.23​ | 36.83±3.25​ | 14.69±1.22​ | 22.22±1.06​ | 53.63±2.04​ |
| 0 h | 50.82±2.05​ | >100​ | >100​ | 36.10±4.41 | 16.24±1.50​ | 18.12±1.00 | 50.95±3.00​ |
| 2 h | >100​ | >100 | >100​ | 49.23±5.25​ | 24.73±2.89​ | 35.80±2.96 | 59.42±2.19​ |
| 4 h | >100 | >100 | >100​ | 89.65±20.16​ | 32.72±1.98​ | 37.50±1.77 | 59.12±3.26​ |
| 6 h | >100 | >100 | >100​ | 87.01±16.29​ | 43.14±3.12​ | 41.18±5.07 | 67.43±2.08​ |
| 12 h |  |  | >100​ | >100​ | 55.62±8.93​ | 64.55±6.87 | 68.70±3.86​ |
| 24 h |  |  | >100​ | >100​ | 69.58±8.61​ | 54.46±1.12 | 68.43±4.00​ |


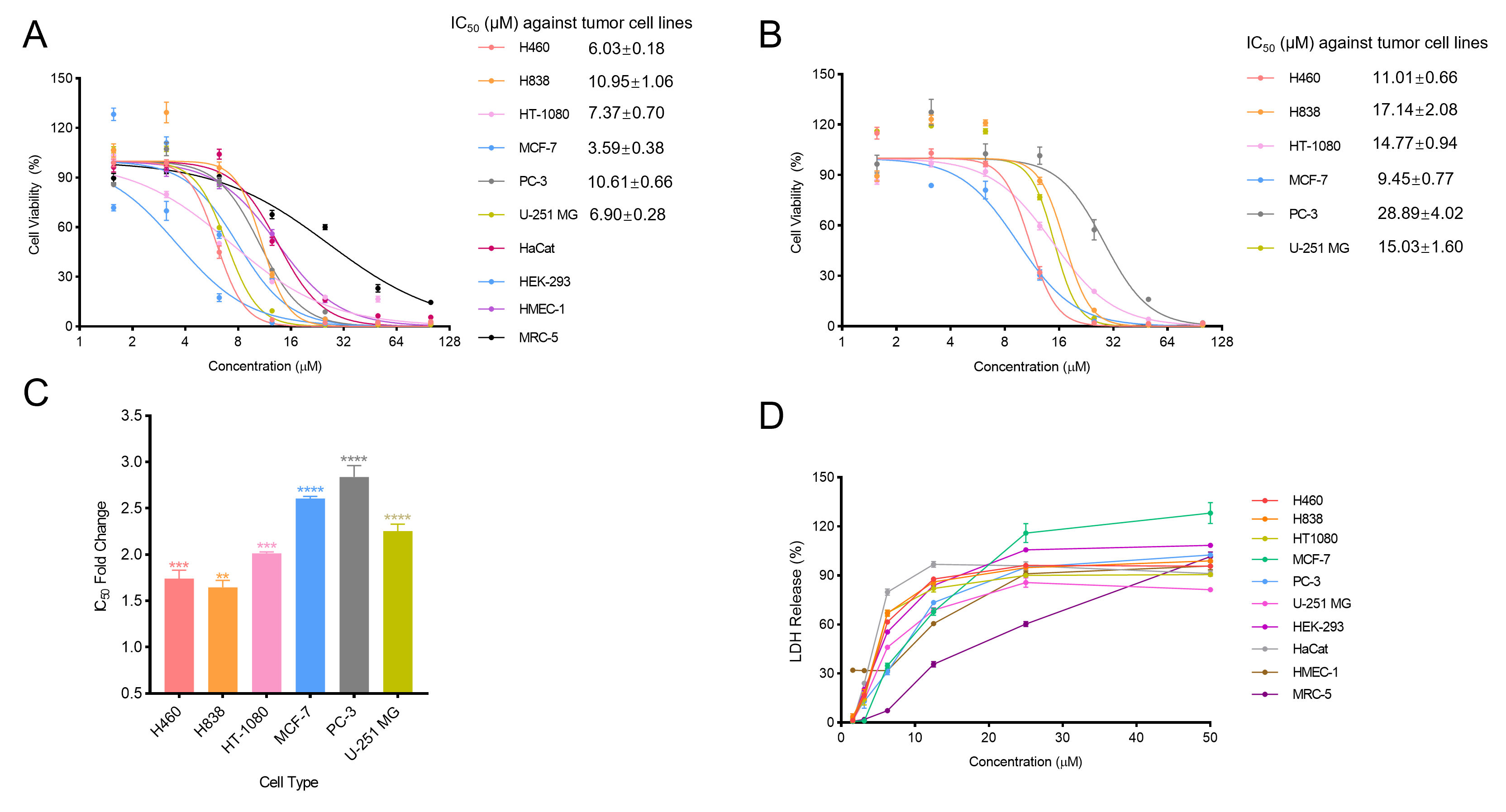


Figure S37. (A) Antiproliferative effect of compounds 111 against different cell lines detected by MTT assays and IC_50_ values of compound 111 against different tumor cell lines. (B) Antiproliferative activity and IC₅₀ values of compound 111 following 4 h pre-incubation with 50% human serum, assessed by MTT assays across various tumor cell lines to evaluate its serum stability. (C) Fold change in IC₅₀ values of compound 111 across different tumor cell lines after pre-incubation with human serum, compared to untreated conditions (control group, fold change = 1, not presented). Data are presented as mean ± SEM, n=3. One-way ANOVA followed by Dunnett's multiple comparisons test was used to compare each treatment group with the control. ** for P < 0.01, *** for P < 0.001, and **** for P < 0.0001. (D) LDH release (%) of compound 111 against different cell lines after 24 h-treatment.


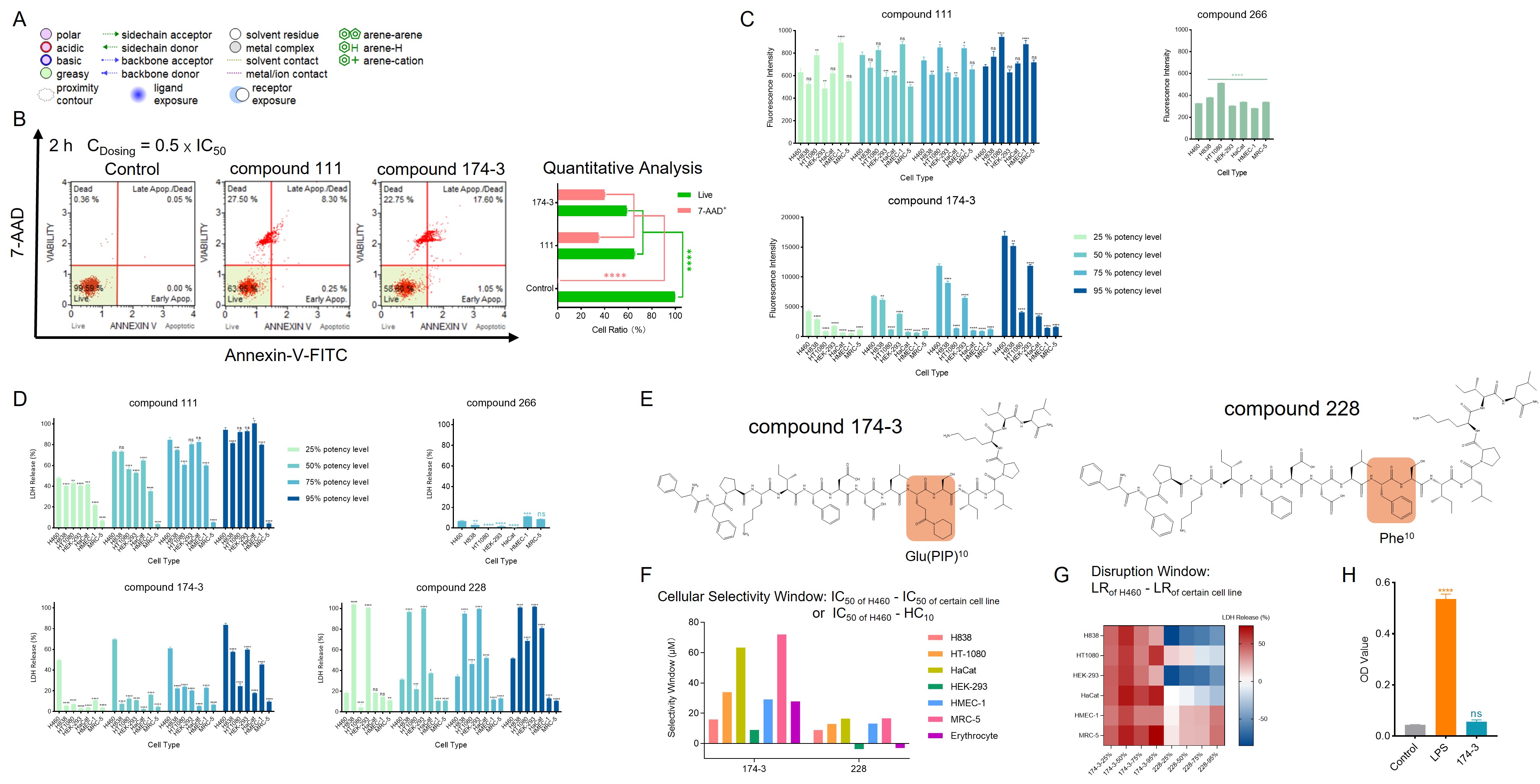


Figure S38. (A) Interpretation of the legend in the 2D interaction patterns presented by MOE. (B) Annexin V/7-AAD-based flow cytometry analysis of H460 cells treated with compounds 111 or 174-3. (C) Cell binding of three compounds to different cell lines after 2 h-treatment, evaluated by fluorescence intensity. (D) LDH release (%) of different cell lines after 24 h-treatment with four compounds. The assays presented in (C) and (D) were performed with compound 266 at a dosing concentration of 12 μM. (E) Chemical structure comparison of compounds 174-3 and 228. Cellular Selectivity window (F) and disruption window (G) of compounds 174-3 and 228 against different cell lines. (H) TNF-α secretion levels of RAW264.7 cells after incubation with compound 174-3 or LPS for 24 h measured by ELISA. Data are presented as mean ± SEM, n=3 (B, C and D) or n=2 (H). One-way ANOVA followed by Dunnett's multiple comparisons test was used to compare each treatment group with the control group in (B) and (H), or with the H460 dosing group at the same potency level in (C and D). ns for P > 0.05, * for P < 0.05, ** for P < 0.01, *** for P < 0.001, and **** for P < 0.0001.


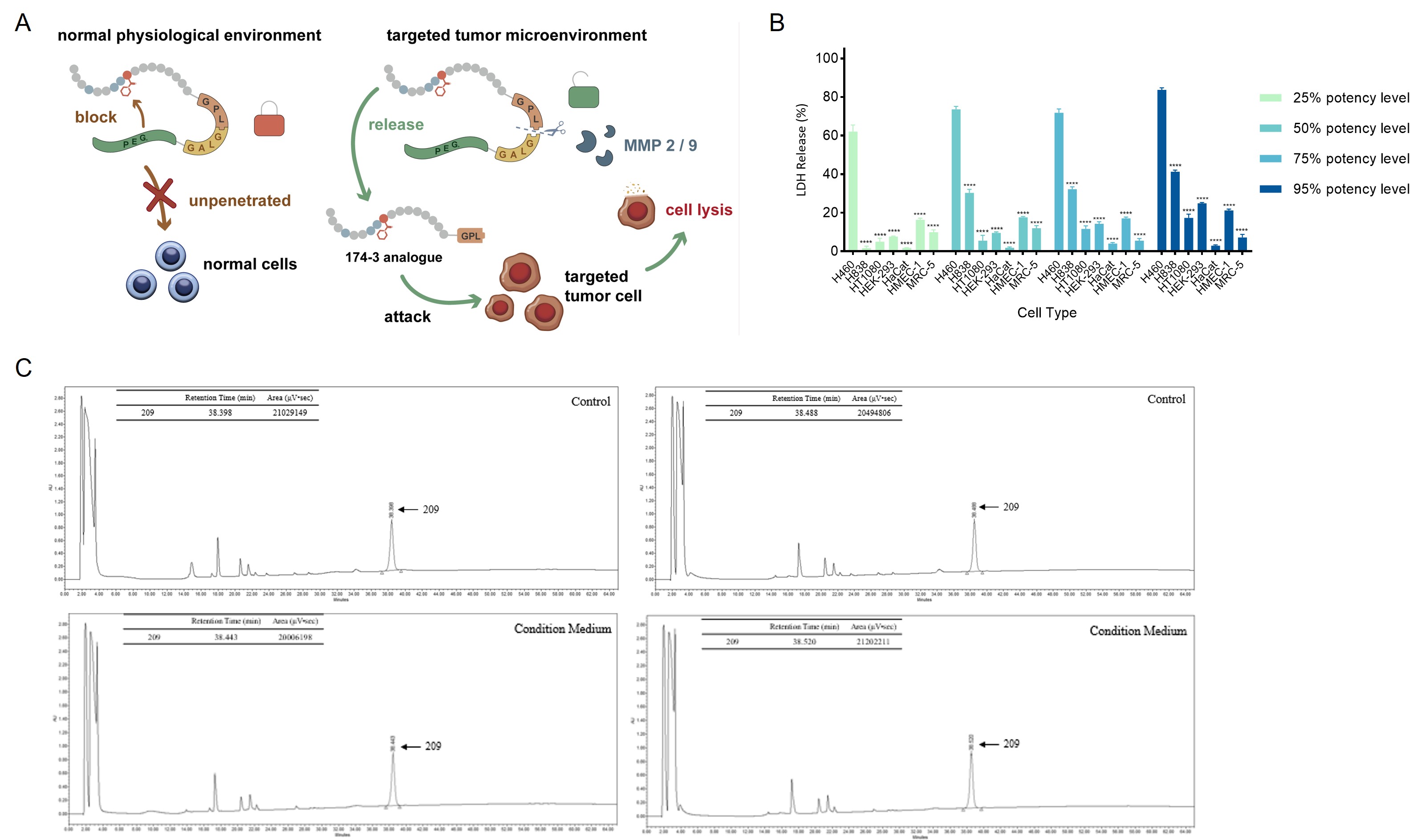


Figure S39. (A) Schematic diagram of the hypothetical mechanism of the MMP-cleavable linker and PEG blocker. (B) LDH release (%) of different cell lines after 24 h-treatment with conjugate 209. Data are presented as mean ± SEM, n=3. One-way ANOVA followed by Dunnett's multiple comparisons test was used to compare each treatment group with the H460 dosing group at the same potency level. **** for P < 0.0001. (C) HPLC analysis of conjugate 209 samples after incubation with control or condition medium for 10 h. Left and right panels represent two independent biological replicates (n = 2).


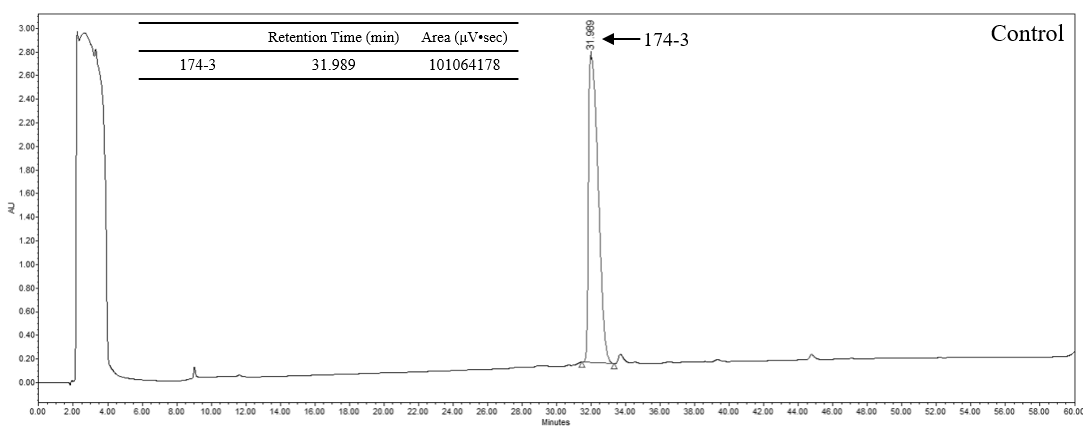

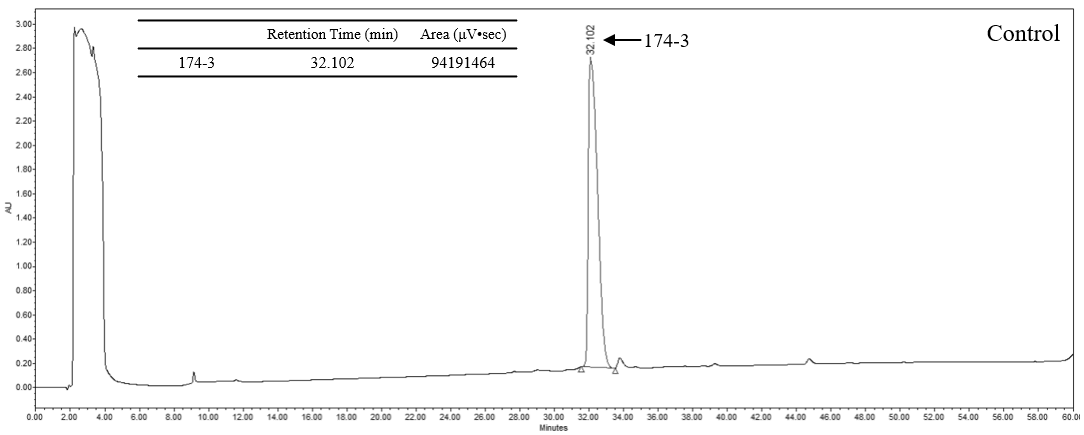


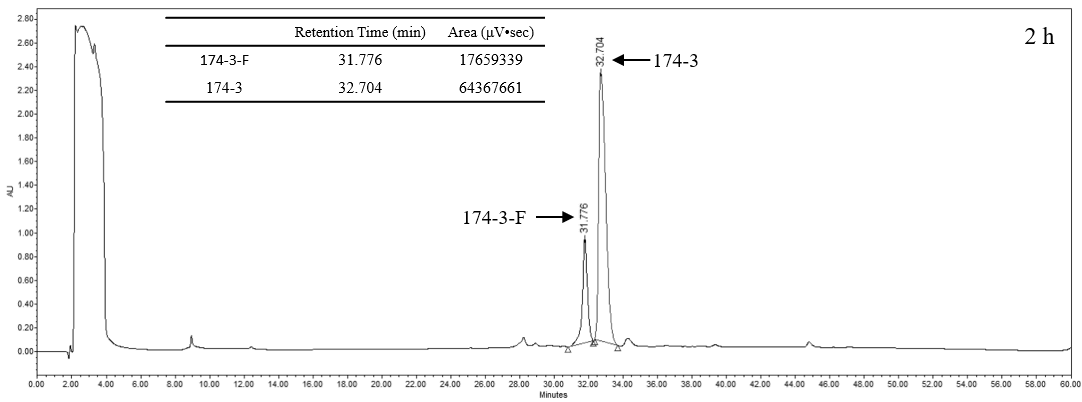

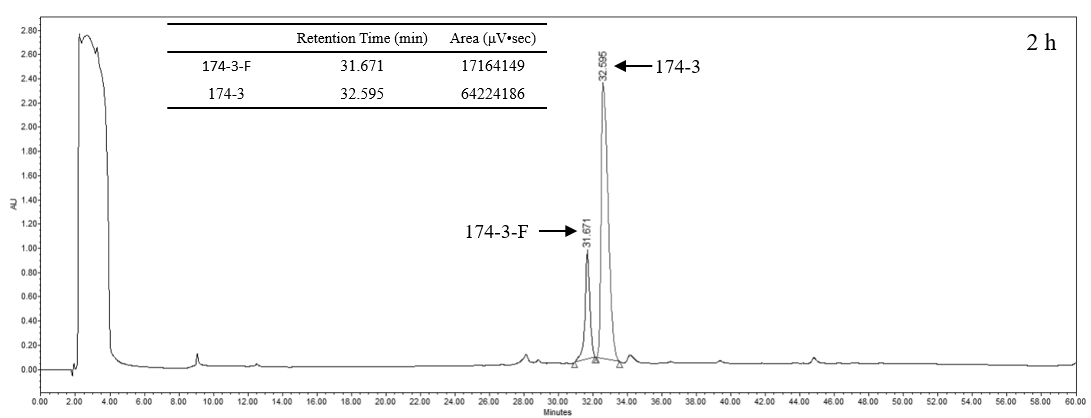


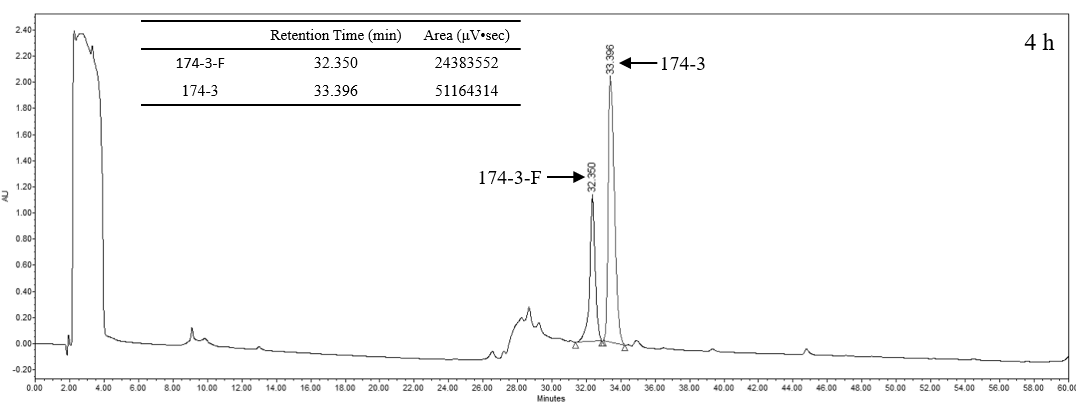

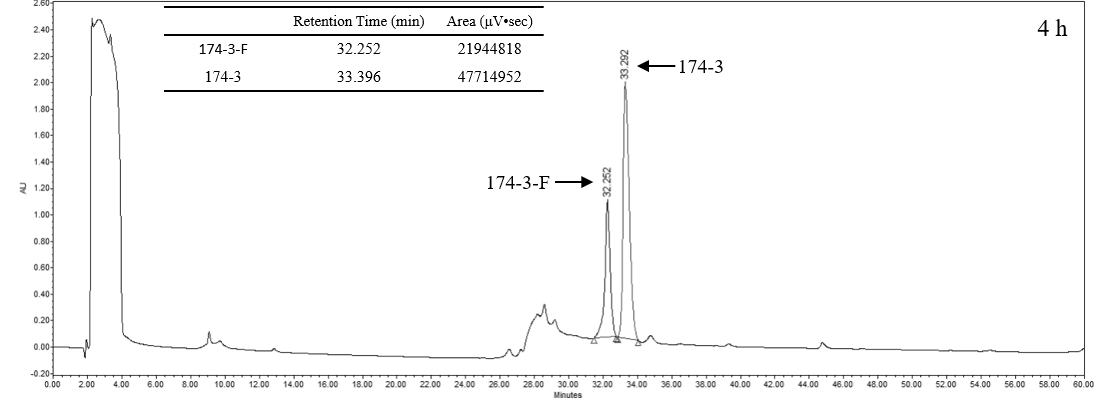


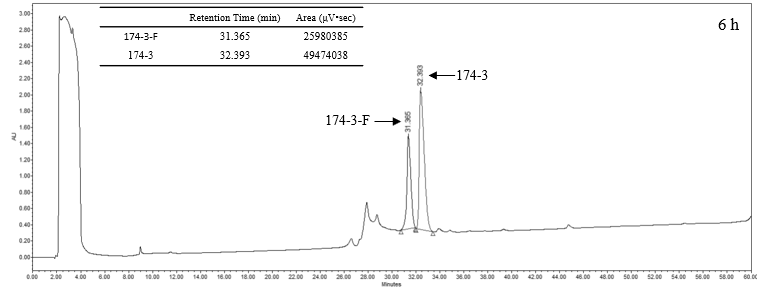

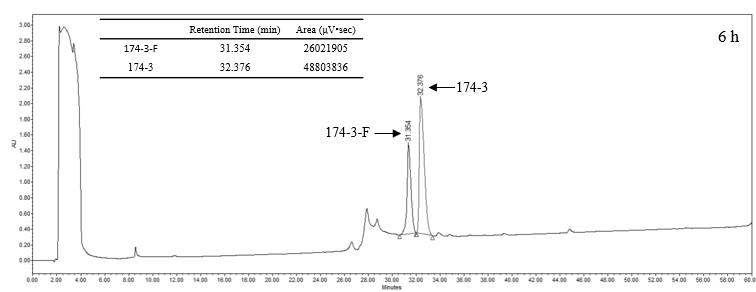


Figure S40. HPLC analysis of compound 174-3 incubated with 50% human serum for 0, 2, 4 and 6 h. Left and right panels represent two independent biological replicates (n = 2).


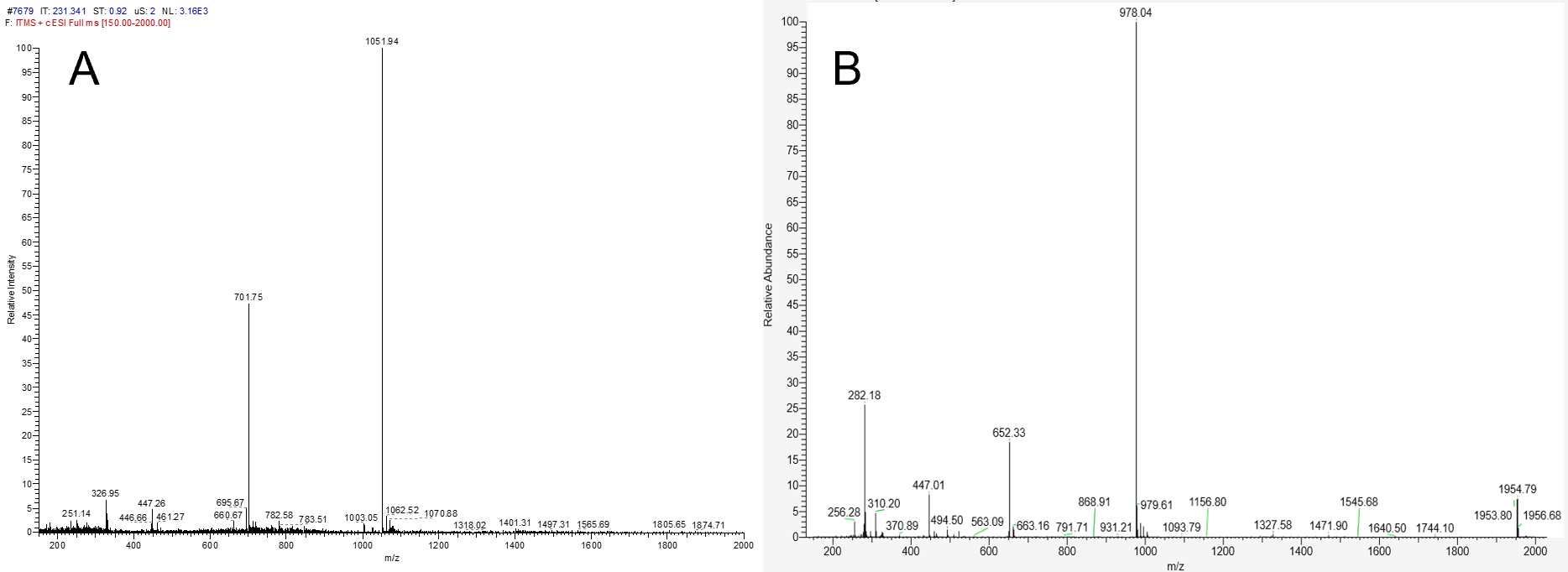


Figure S41. MS spectra of compound 174-3 peak (A) and compound 174-3-F peak (B). Compound 174-3-F calculated: [M+2H]^2+^: 978.22, [M+3H]^3+^: 652.48; found: [M+2H]^2+^: 978.04, [M+3H]^3+^: 652.33.

Table S3. MS/MS fragmentation sequencing data of compound 174-3-F.

| 174-3-F | FPKIFDDLE(PIP)SILPKIL-NH_2_ | | | | | | | |
| --- | --- | --- | --- | --- | --- | --- | --- | --- |
|  | calculated | found | |  | | calculated | | found |
| b_1_^+^ | 148.08 | |  | | y_1_^+^ | | 1807.10 |  |
| b_2_^+^ | 245.13 | |  | | y_2_^+^ | | 1710.05 | 1709.82 |
| b_3_^+^ | 373.22 | | 373.18 | | y_3_^+^ | | 1581.95 | 1581.62 |
| b_4_^+^ | 486.31 | | 486.21 | | y_4_^+^ | | 1468.87 | 1468.59 |
| b_5_^+^ | 633.37 | | 633.19 | | y_5_^+^ | | 1321.80 | 1321.46 |
| b_6_^+^ | 748.40 | |  | | y_6_^+^ | | 1206.77 | 1206.41 |
| b_7_^+^ | 863.43 | | 863.21 | | y_7_^+^ | | 1091.75 | 1091.39 |
| b_8_^+^ | 976.51 | |  | | y_8_^+^ | | 978.66 | 978.54 |
| b_9_^+^ | 1172.63 | | 1172.35 | | y_9_^+^ | | 782.54 |  |
| b_10_^+^ | 1259.67 | | 1259.41 | | y_10_^+^ | | 695.51 |  |
| b_11_^+^ | 1372.75 | | 1372.45 | | y_11_^+^ | | 582.43 | 582.22 |
| b_12_^+^ | 1485.83 | | 1485.51 | | y_12_^+^ | | 469.34 | 469.19 |
| b_13_^+^ | 1582.89 | |  | | y_13_^+^ | | 372.29 |  |
| b_14_^+^ | 1710.98 | |  | | y_14_^+^ | | 244.19 |  |
| b_15_^+^ | 1824.07 | |  | | y_15_^+^ | | 131.11 |  |
| b_16_^+^ | 1937.15 | |  | | y_16_^+^ | | 17.03 |  |


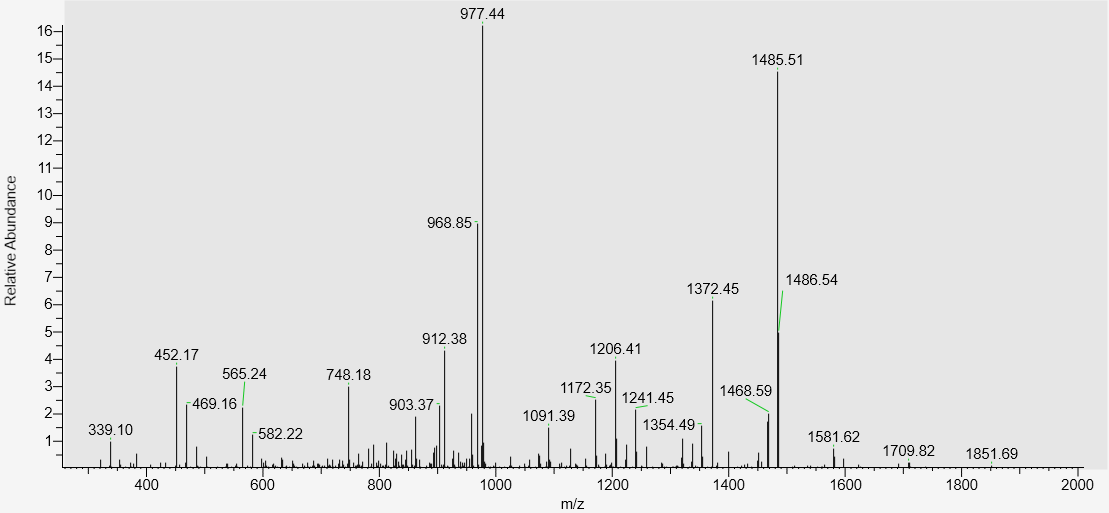


Figure S42. MS/MS spectrum of compound 174-3-F.


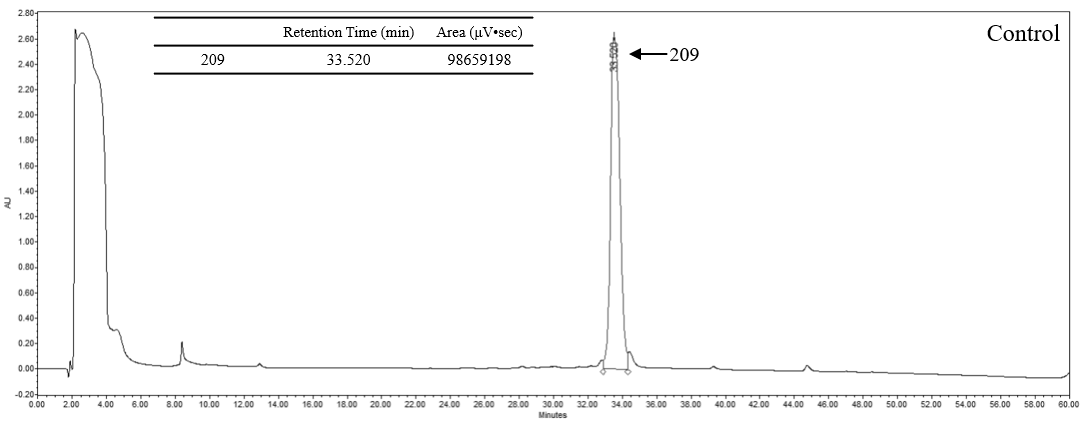

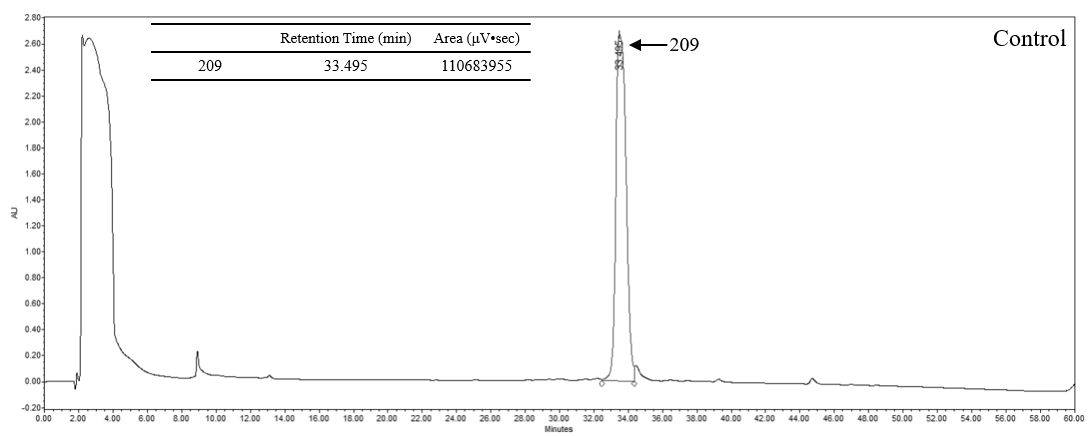


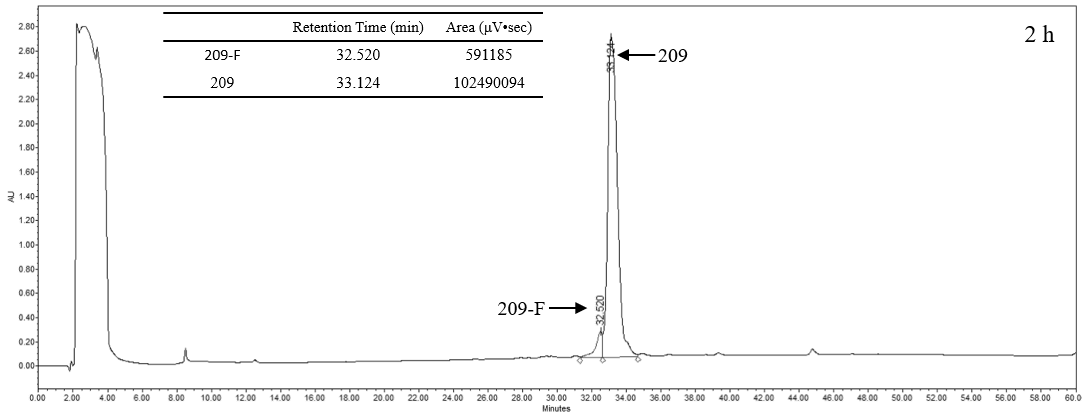

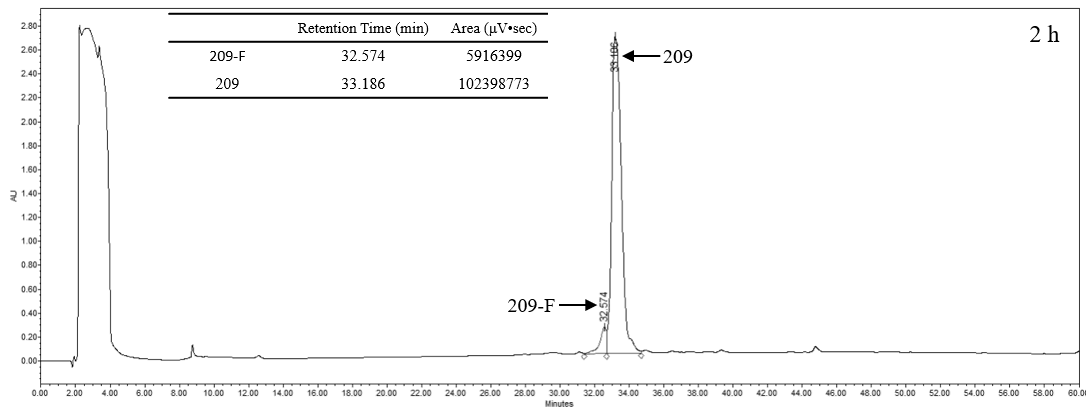


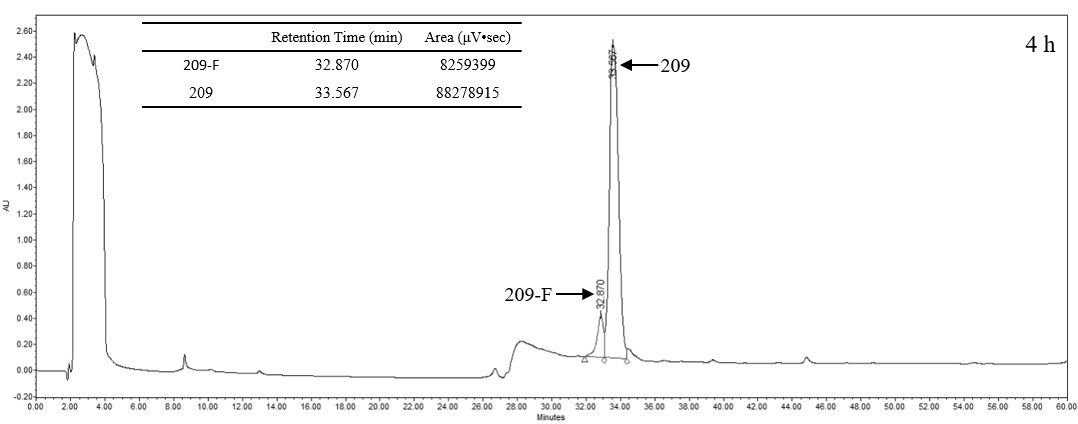

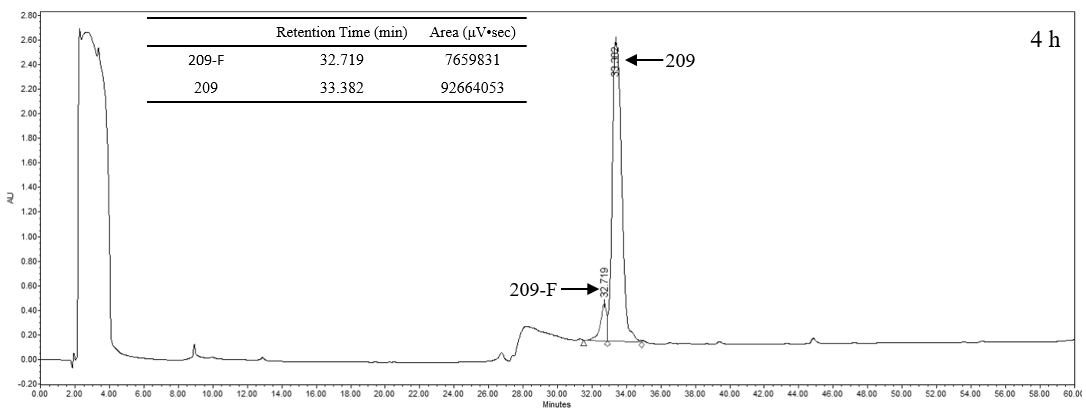


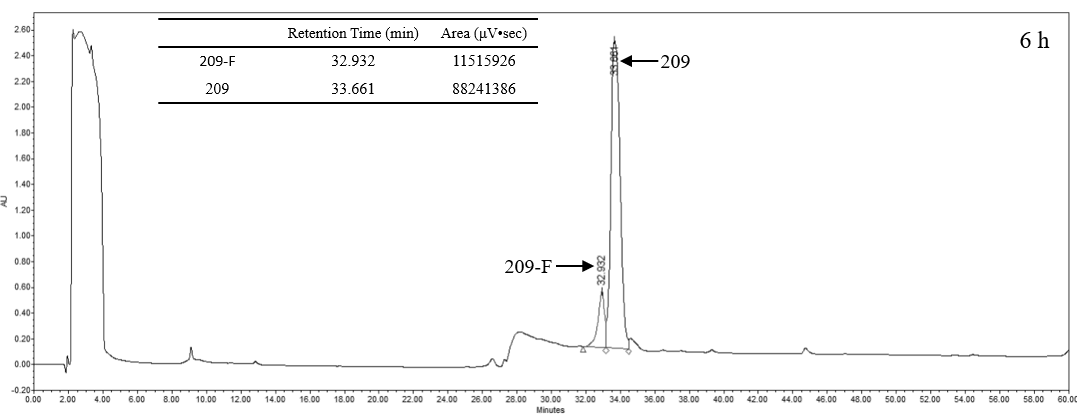

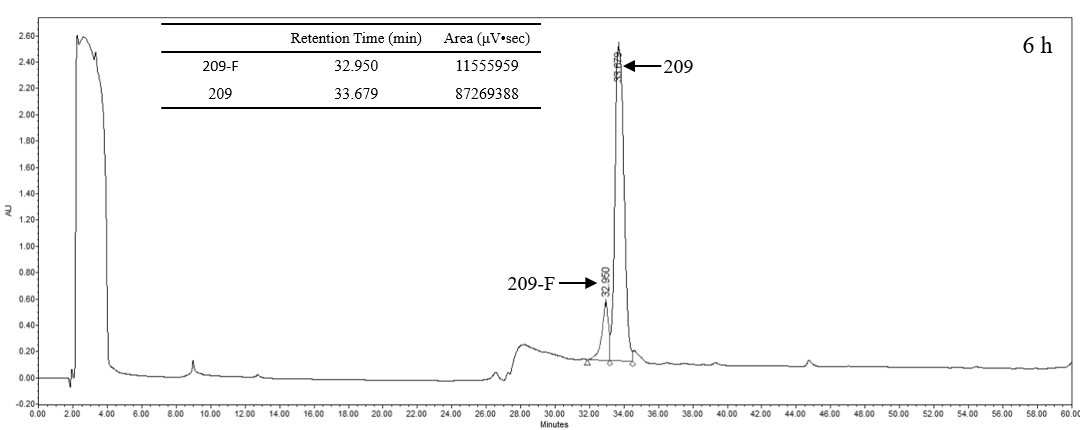


Figure S43. HPLC analysis of conjugate 209 incubated with 50% human serum for 0, 2, 4 and 6 h. Left and right panels represent two independent biological replicates (n = 2).


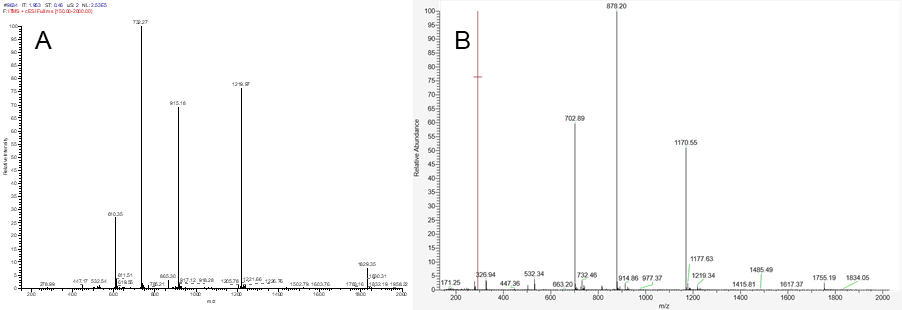


Figure S44. MS spectra of conjugate 209 peak (A) and compound 209-F peak (B). Conjugate 209-F calculated: [M+3H]^3+^: 1170.76, [M+4H]^4+^: 878.32, [M+5H]^5+^: 702.85; found: [M+3H]^3+^: 1170.55, [M+4H]^4+^: 878.20, [M+5H]^5+^: 702.89.

Table S4. MS/MS fragmentation sequencing data of conjugate 209-F.

| 209-F |  | FPKIFDDLE(PIP)SILPKILGPLGLAG(PEG_4_)_4_-NH_2_ | | | | | |  |
| --- | --- | --- | --- | --- | --- | --- | --- | --- |
|  | calculated | found | |  | | calculated | | found |
| b_1_^+^ | 149.08 | |  | | y_1_^2+^ | | 1681.00 |  |
| b_2_^+^ | 246.14 | |  | | y_2_^2+^ | | 1632.47 |  |
| b_3_^+^ | 374.23 | |  | | y_3_^2+^ | | 1568.42 |  |
| b_4_^+^ | 487.32 | |  | | y_4_^2+^ | | 1511.88 |  |
| b_5_^+^ | 634.38 | | 634.11 | | y_5_^2+^ | | 1438.35 |  |
| b_6_^+^ | 749.41 | |  | | y_6_^2+^ | | 1380.32 | 1380.97 |
| b_7_^+^ | 864.44 | | 864.17 | | y_7_^2+^ | | 1323.32 |  |
| b_8_^+^ | 977.52 | | 977.53 | | y_8_^2+^ | | 1266.78 |  |
| b_9_^+^ | 1173.64 | |  | | y_9_^2+^ | | 1168.72 |  |
| b_10_^+^ | 1260.68 | |  | | y_10_^2+^ | | 1125.20 |  |
| b_11_^+^ | 1373.76 | | 1373.19 | | y_11_^2+^ | | 1068.66 | 1068.81 |
| b_12_^+^ | 1486.84 | | 1486.58 | | y_12_^2+^ | | 1012.12 | 1012.25 |
| b_13_^+^ | 1583.90 | | 1583.88 | | y_13_^+^ | | 1926.18 |  |
| b_14_^+^ | 1711.99 | |  | | y_14_^+^ | | 1798.08 |  |
| b_15_^+^ | 1825.08 | | 1824.77 | | y_15_^+^ | | 1684.00 |  |
| b_16_^+^ | 1952.18 | |  | | y_16_^+^ | | 1571.92 |  |
| b_17_^2+^ | 1005.10 | | 1004.88 | | y_17_^+^ | | 1514.90 | 1514.62 |
| b_18_^2+^ | 1053.63 | |  | | y_18_^+^ | | 1417.84 | 1417.91 |
| b_19_^2+^ | 1110.17 | | 1110.21 | | y_19_^+^ | | 1304.76 | 1304.69 |
| b_20_^2+^ | 1138.68 | |  | | y_20_^+^ | | 1247.74 | 1247.51 |
| b_21_^2+^ | 1195.22 | |  | | y_21_^+^ | | 1134.65 | 1134.35 |
| b_22_^2+^ | 1230.74 | |  | | y_22_^+^ | | 1063.62 | 1063.58 |
| b_23_^2+^ | 1382.82 | |  | | y_23_^+^ | | 759.45 |  |
| b_24_^2+^ | 1506.39 | | 1505.69 | | y_24_^+^ | | 512.31 |  |
| b_25_^2+^ | 1629.69 | |  | | y_25_^+^ | | 265.17 |  |
| b_26_^2+^ | 1753.54 | |  | | y_26_^+^ | | 17.03 |  |


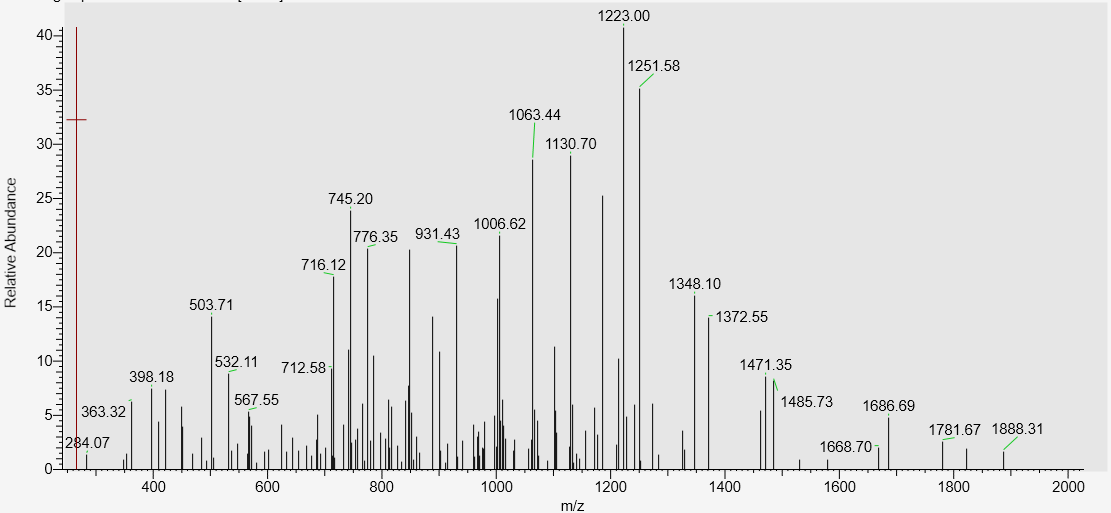


Figure S45. MS/MS spectrum of conjugate 209-F.


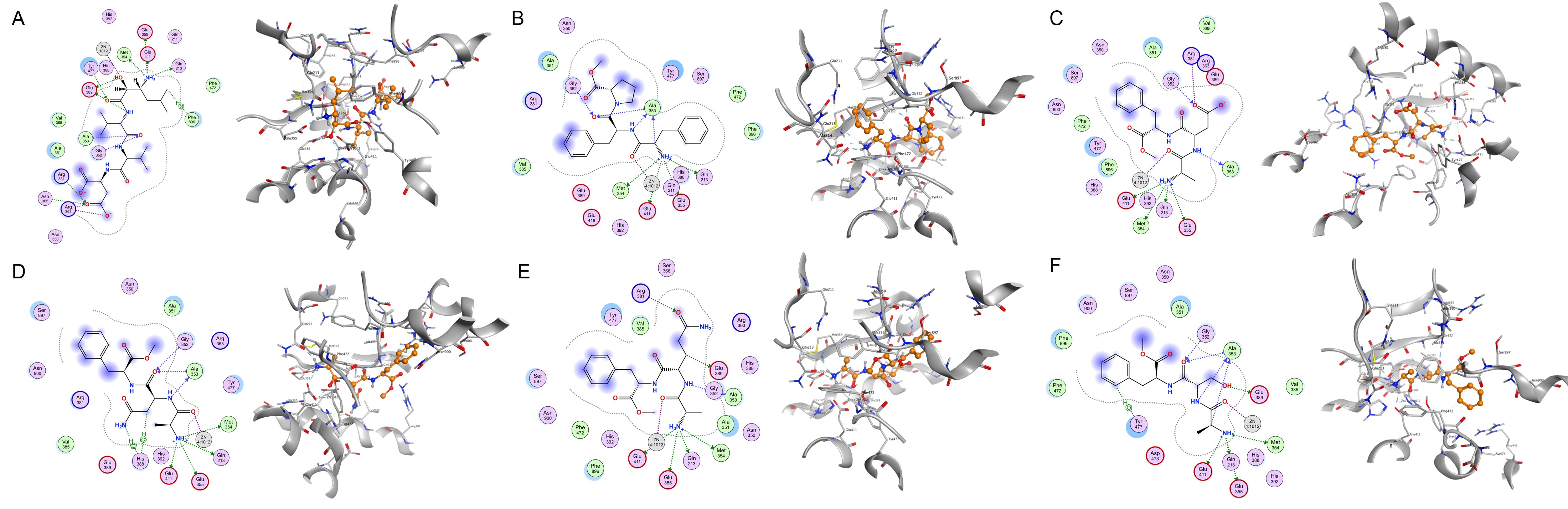


Figure S46. Predicted binding patterns between APN and six ligands: amastatin (control, A), and tripeptides FFP (B), ADF (C), ANF (D), AQF (E), and ASF (F). Left panels show 2D interaction diagrams; right panels show 3D cartoon representations. In the 3D diagrams, receptors are shown in grey and ligands in orange. Blue dashed lines indicate hydrogen bonds. For interpretation of the legend in the 2D interaction patterns, refer to Figure S38 A.


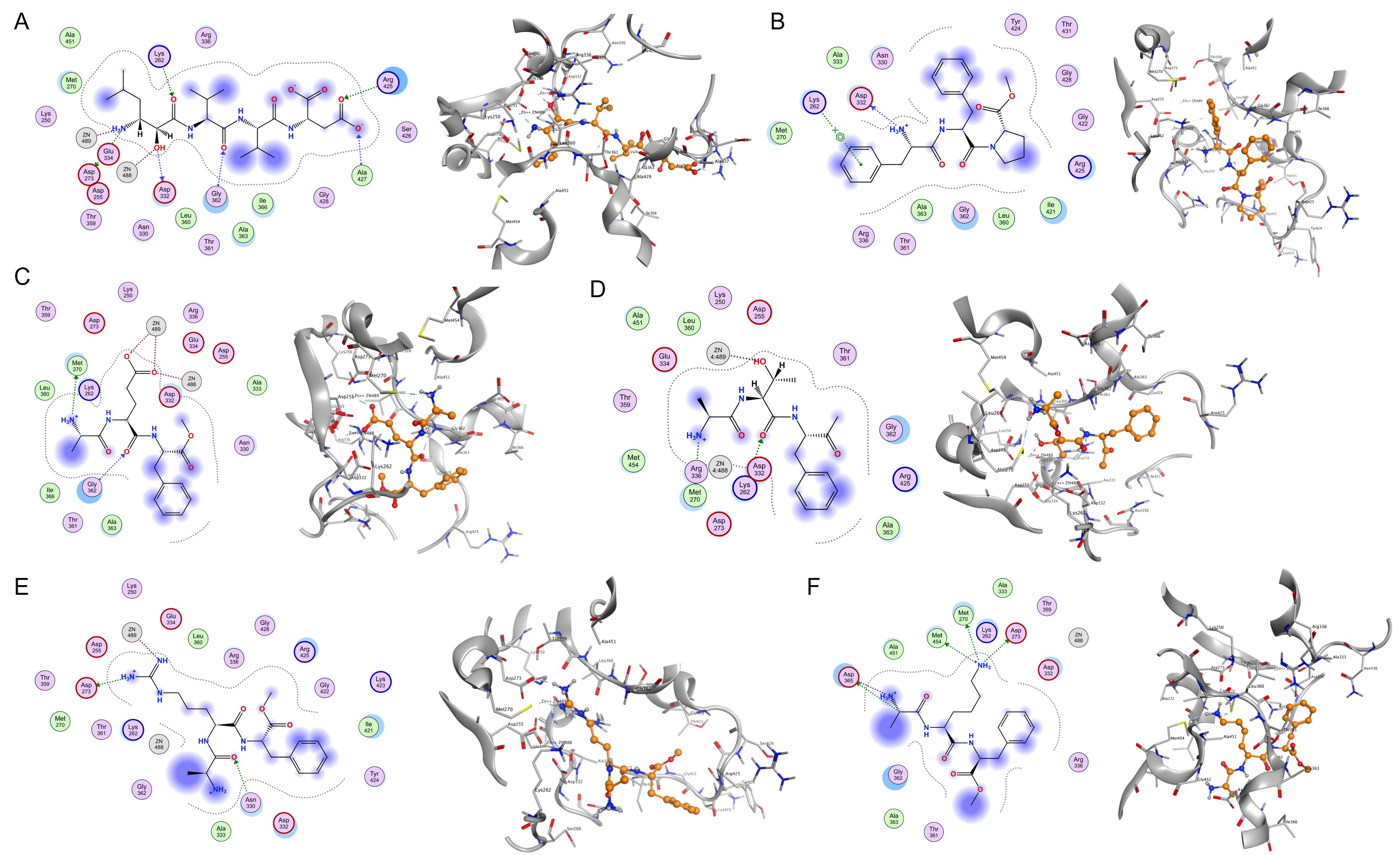


Figure S47. Predicted docking 2D interaction patterns (left) and 3D cartoon interaction patterns (right) between LAP and amastatin (control, A), tripeptides FFP (B), AEF (C), ATF (D), ARF (E) and AKF (F). In 3D cartoon interaction, receptors are grey, and ligands are orange. Blue dashed lines represent hydrogen bonds. For interpretation of the legend in the 2D interaction patterns, refer to Figure S38 A.


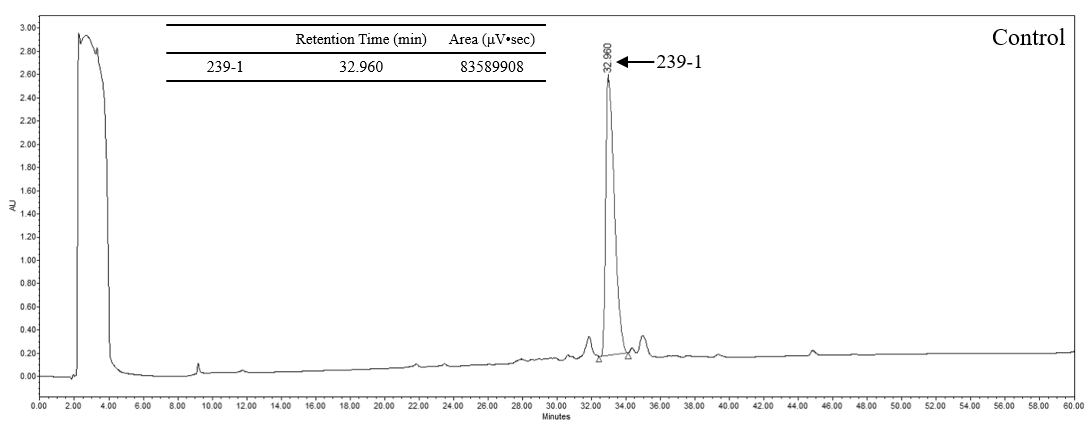

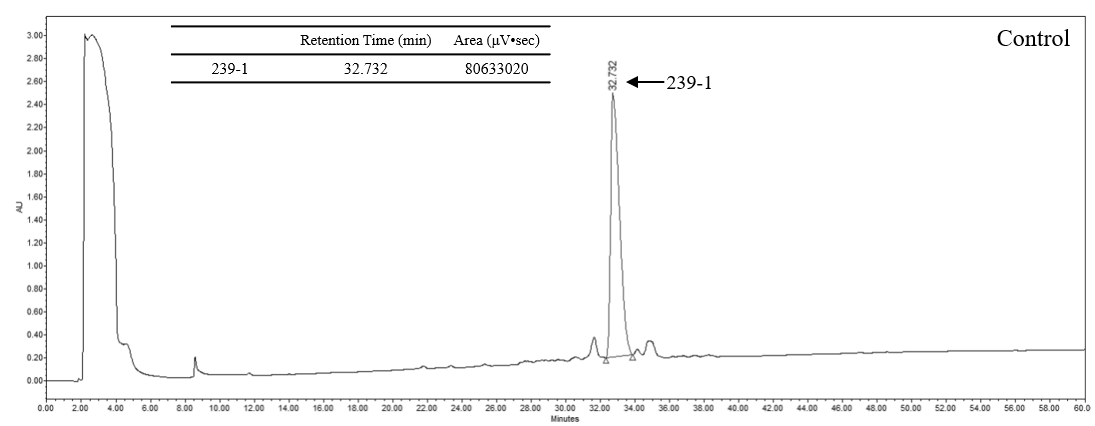


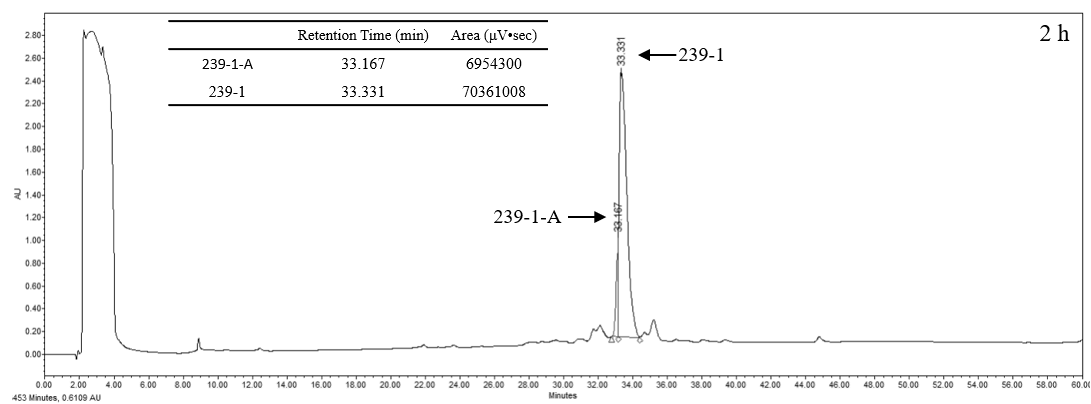

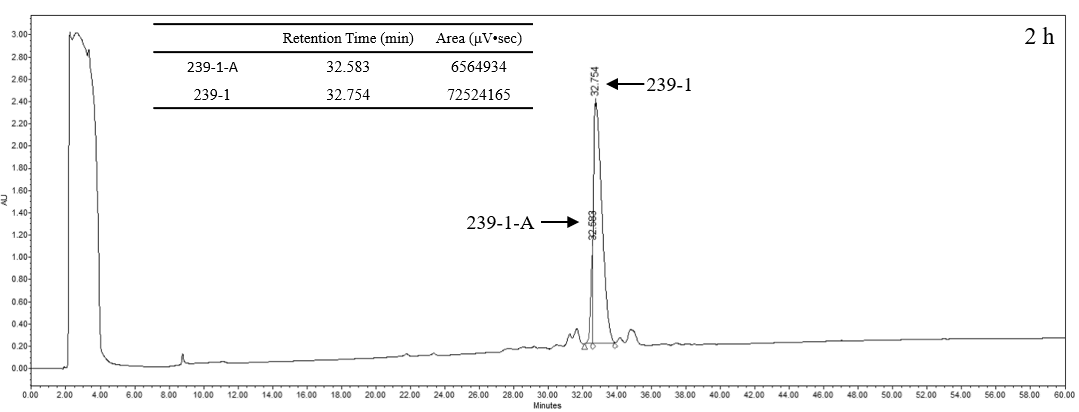


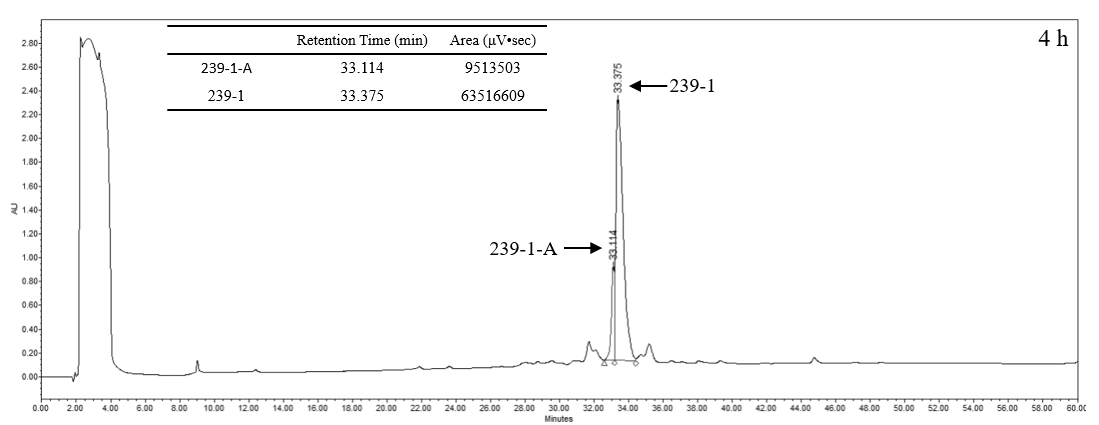

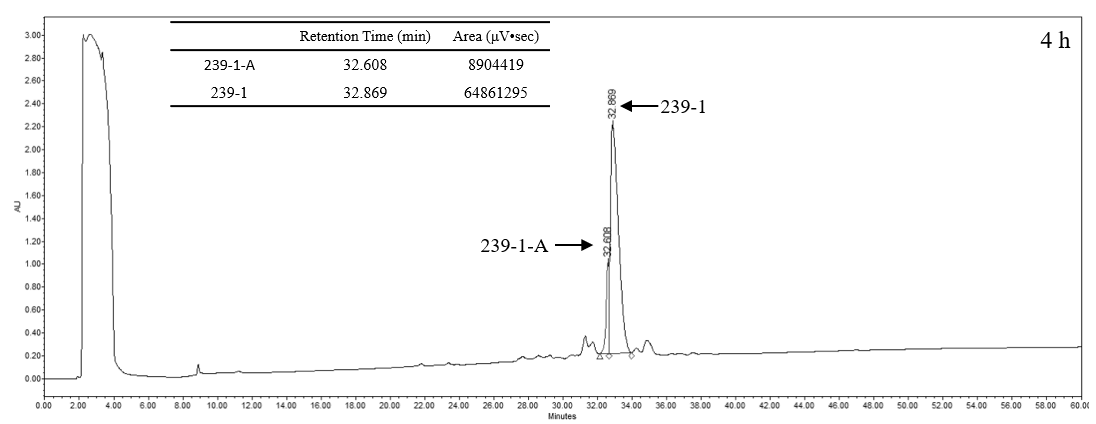


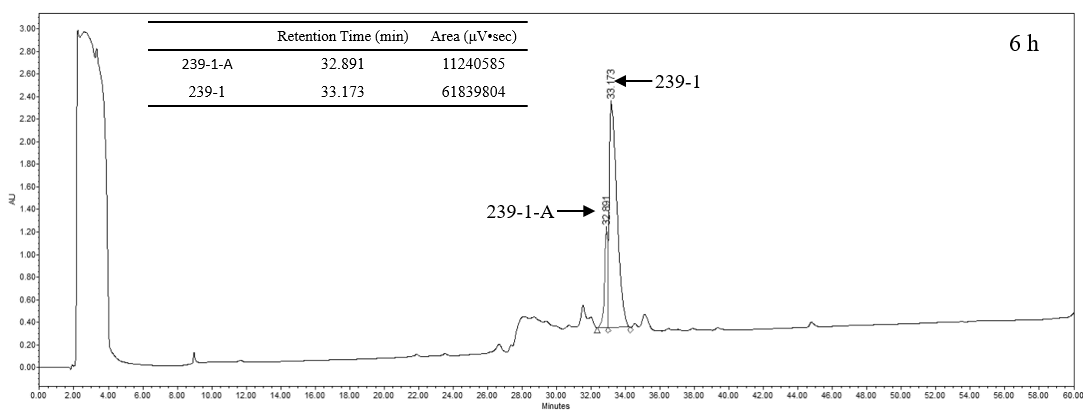

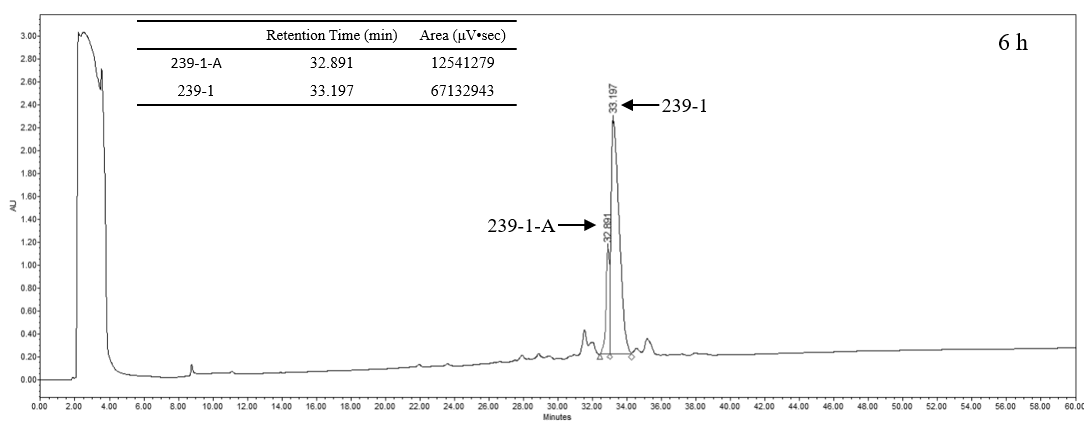


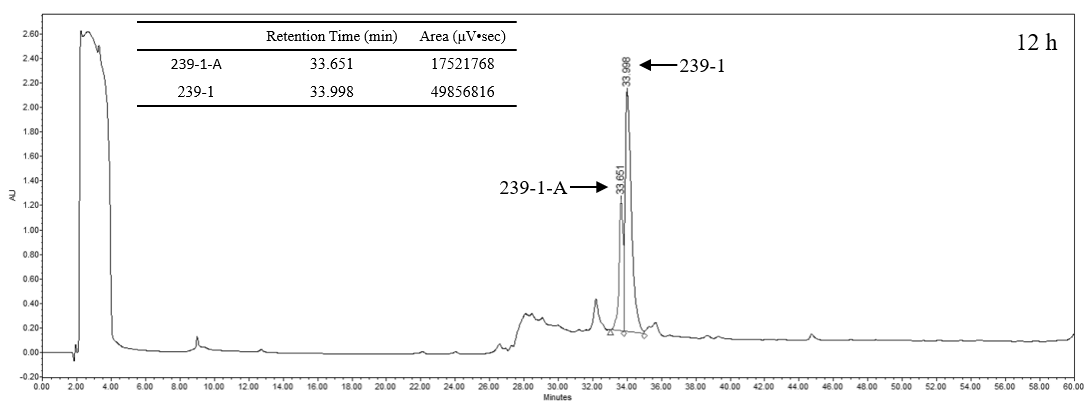

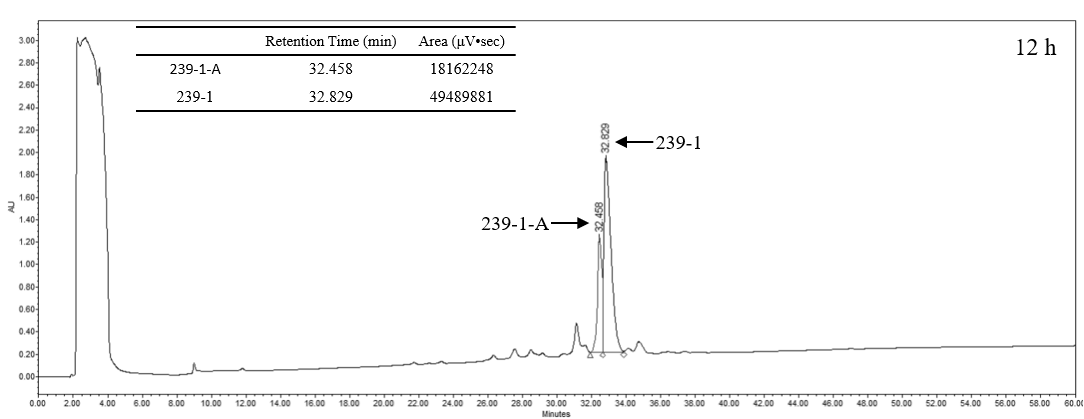


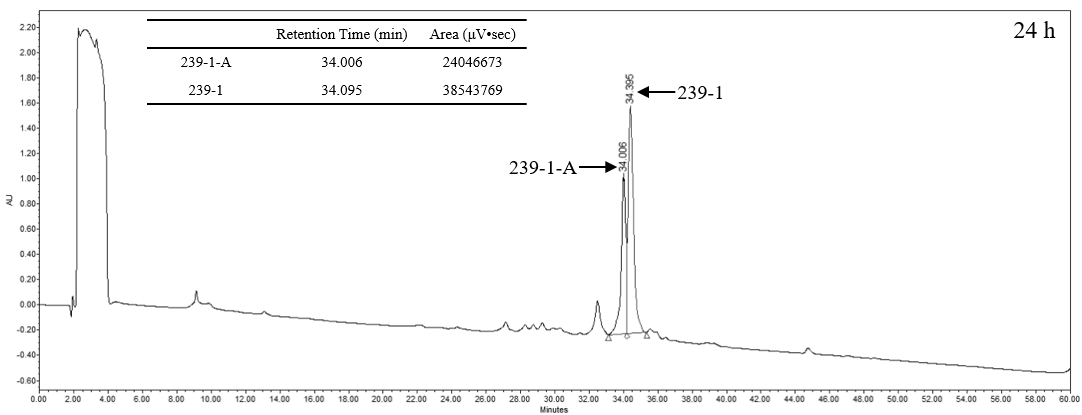

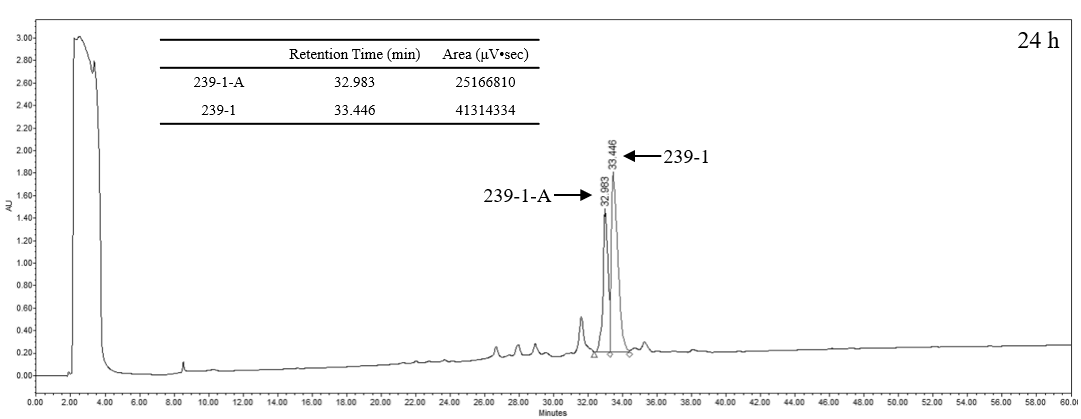


Figure S48. HPLC analysis of compound 239-1 incubated with 50% human serum for 0, 2, 4, 6, 12 and 24 h. Left and right panels represent two independent biological replicates (n = 2).


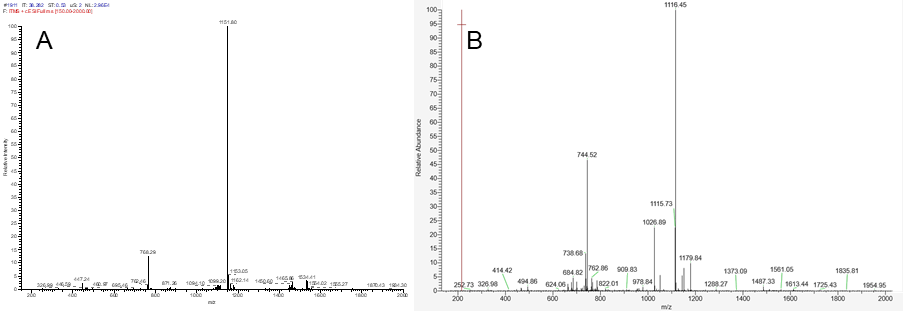


Figure S49. MS spectra of compound 239-1 peak (A) and compound 239-1-A peak (B). Compound 239-1-A calculated: [M+2H]^2+^: 1116.37, [M+3H]^3+^: 744.58; found: [M+2H]^2+^: 1116.45, [M+3H]^3+^: 744.52.

Table S5. MS/MS fragmentation sequencing data of compound 239-1-A.

| 239-1-A |  | EFFPKIFDDLE(PIP)SILPKIL-NH_2_ | | | | | |  |
| --- | --- | --- | --- | --- | --- | --- | --- | --- |
|  | calculated | found | |  | | calculated | | found |
| b_1_^+^ | 131.06 | |  | | y_1_^2+^ | | 1116.65 | 1115.58 |
| b_2_^+^ | 278.13 | |  | | y_2_^+^ | | 1953.17 |  |
| b_3_^+^ | 425.20 | | 425.02 | | y_3_^+^ | | 1807.10 | 1807.07 |
| b_4_^+^ | 522.25 | |  | | y_4_^+^ | | 1710.05 |  |
| b_5_^+^ | 650.34 | |  | | y_5_^+^ | | 1581.95 | 1581.86 |
| b_6_^+^ | 763.43 | |  | | y_6_^+^ | | 1468.87 | 1468.82 |
| b_7_^+^ | 910.50 | |  | | y_7_^+^ | | 1321.80 | 1321.76 |
| b_8_^+^ | 1025.52 | | 1025.55 | | y_8_^+^ | | 1206.77 | 1206.70 |
| b_9_^+^ | 1140.55 | |  | | y_9_^+^ | | 1091.75 | 1091.62 |
| b_10_^+^ | 1253.63 | | 1253.63 | | y_10_^+^ | | 978.66 | 978.61 |
| b_11_^+^ | 1449.75 | |  | | y_11_^+^ | | 782.54 | 782.32 |
| b_12_^+^ | 1536.79 | |  | | y_12_^+^ | | 695.51 | 695.24 |
| b_13_^+^ | 1649.87 | |  | | y_13_^+^ | | 582.43 |  |
| b_14_^+^ | 1762.95 | |  | | y_14_^+^ | | 469.34 | 469.25 |
| b_15_^+^ | 1860.01 | |  | | y_15_^+^ | | 372.29 |  |
| b_16_^+^ | 1988.10 | |  | | y_16_^+^ | | 244.19 |  |
| b_17_^2+^ | 1051.10 | | 1050.70 | | y_17_^+^ | | 131.11 |  |
| b_18_^2+^ | 1107.64 | | 1107.29 | | y_18_^+^ | | 17.03 |  |


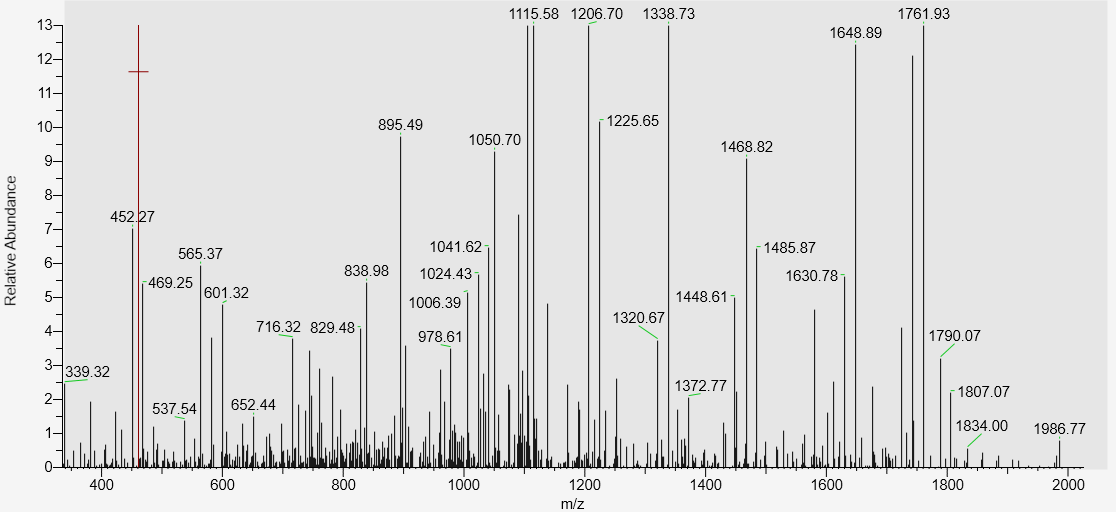


Figure S50. MS/MS spectrum of compound 239-1-A.


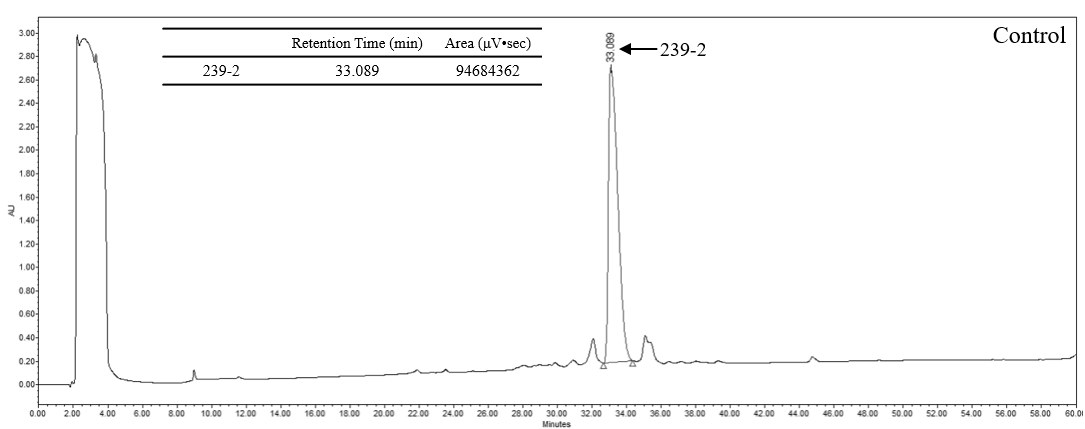

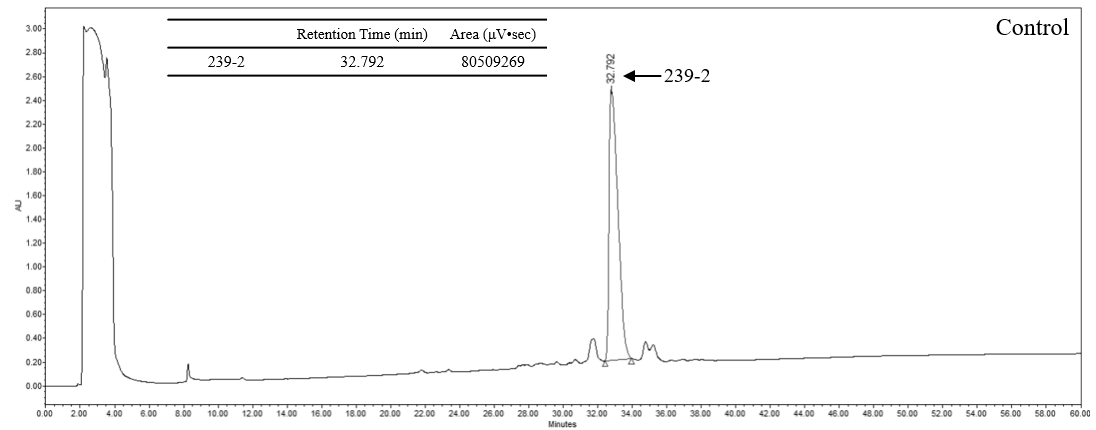


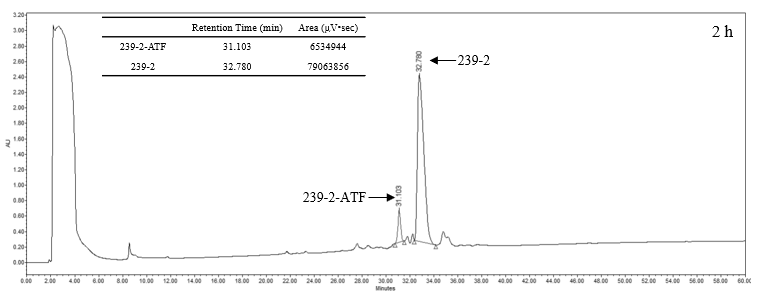

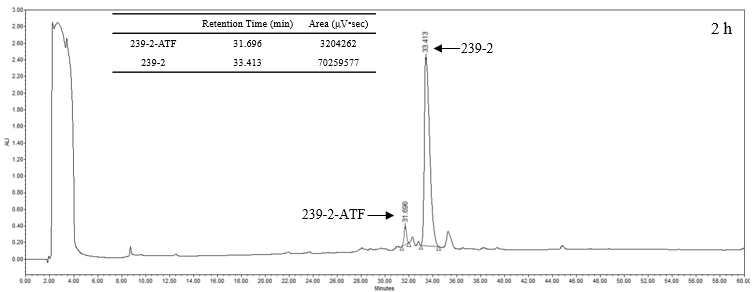


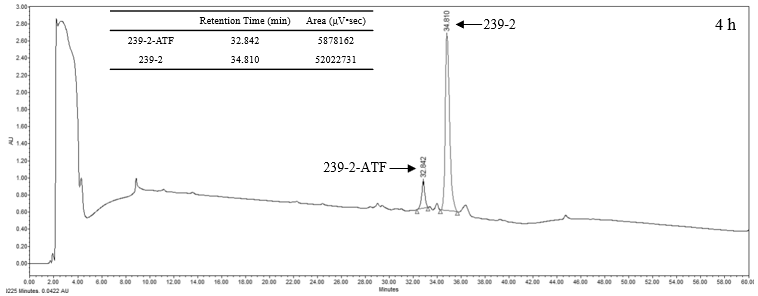

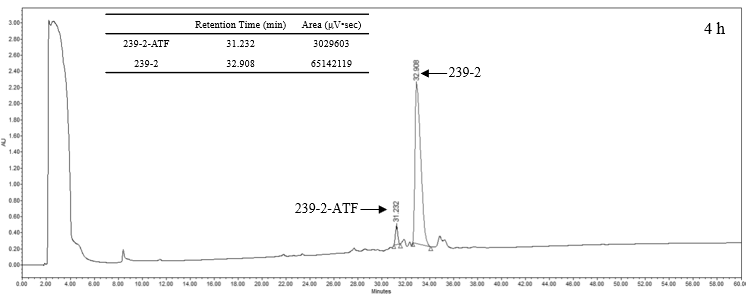


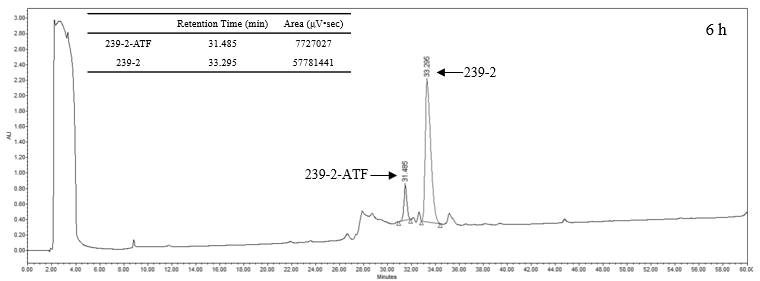

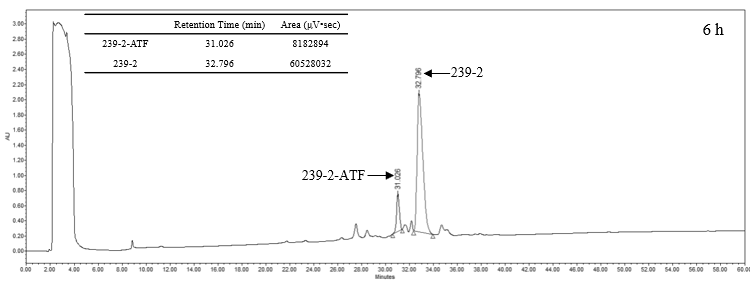


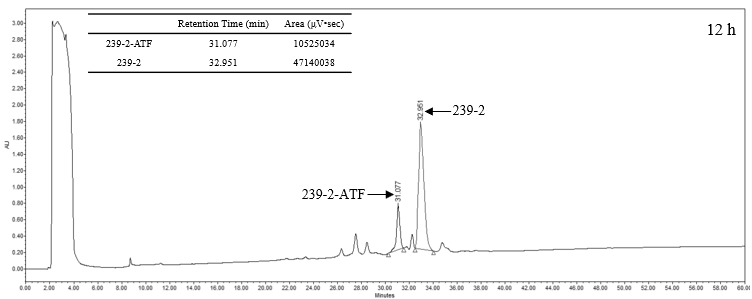

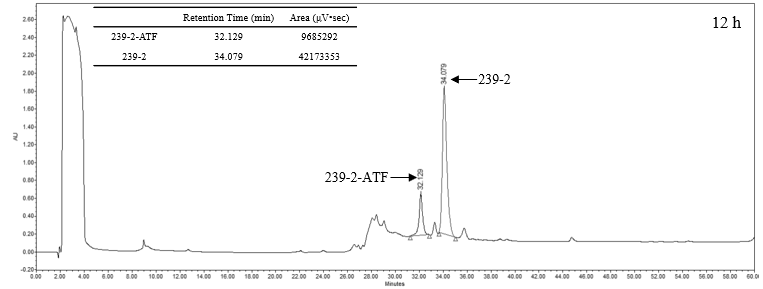


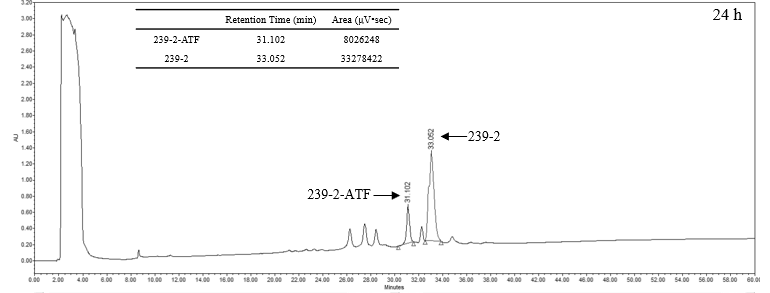

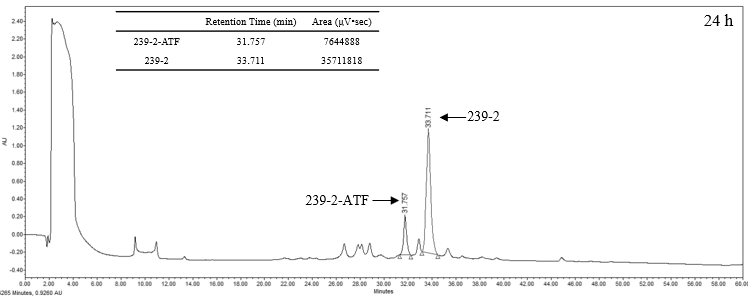


Figure S51. HPLC analysis of compound 239-2 incubated with 50% human serum for 0, 2, 4, 6, 12 and 24 h. Left and right panels represent two independent biological replicates (n = 2).


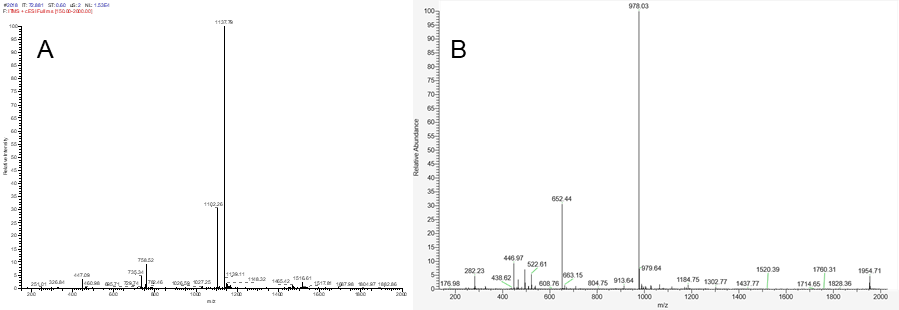


Figure S52. MS spectra of compound 239-2 peak (A) and compound 239-2-ATF peak (B). Compound 239-2-ATF calculated: [M+2H]^2+^: 978.22, [M+3H]^3+^: 652.48; found: [M+2H]^2+^: 978.03, [M+3H]^3+^: 652.44.

Table S6. MS/MS fragmentation sequencing data of compound 239-2-ATF.

| 239-2-ATF | FPKIFDDLE(PIP)SILPKIL-NH_2_ | | | | | | | |
| --- | --- | --- | --- | --- | --- | --- | --- | --- |
|  | calculated | found | |  | | calculated | | found |
| b_1_^+^ | 148.08 | |  | | y_1_^+^ | | 1807.10 |  |
| b_2_^+^ | 245.13 | |  | | y_2_^+^ | | 1710.05 | 1710.52 |
| b_3_^+^ | 373.22 | | 373.16 | | y_3_^+^ | | 1581.95 | 1581.61 |
| b_4_^+^ | 486.31 | | 486.14 | | y_4_^+^ | | 1468.87 | 1468.58 |
| b_5_^+^ | 633.37 | | 633.21 | | y_5_^+^ | | 1321.80 | 1321.54 |
| b_6_^+^ | 748.40 | | 748.22 | | y_6_^+^ | | 1206.77 | 1206.39 |
| b_7_^+^ | 863.43 | | 863.24 | | y_7_^+^ | | 1091.75 | 1091.48 |
| b_8_^+^ | 976.51 | |  | | y_8_^+^ | | 978.66 |  |
| b_9_^+^ | 1172.63 | | 1172.39 | | y_9_^+^ | | 782.54 | 782.28 |
| b_10_^+^ | 1259.67 | | 1259.37 | | y_10_^+^ | | 695.51 |  |
| b_11_^+^ | 1372.75 | | 1372.44 | | y_11_^+^ | | 582.43 | 582.27 |
| b_12_^+^ | 1485.83 | | 1485.51 | | y_12_^+^ | | 469.34 | 469.19 |
| b_13_^+^ | 1582.89 | | 1582.52 | | y_13_^+^ | | 372.29 | 372.25 |
| b_14_^+^ | 1710.98 | | 1710.52 | | y_14_^+^ | | 244.19 |  |
| b_15_^+^ | 1824.07 | |  | | y_15_^+^ | | 131.11 |  |
| b_16_^+^ | 1937.15 | |  | | y_16_^+^ | | 17.03 |  |


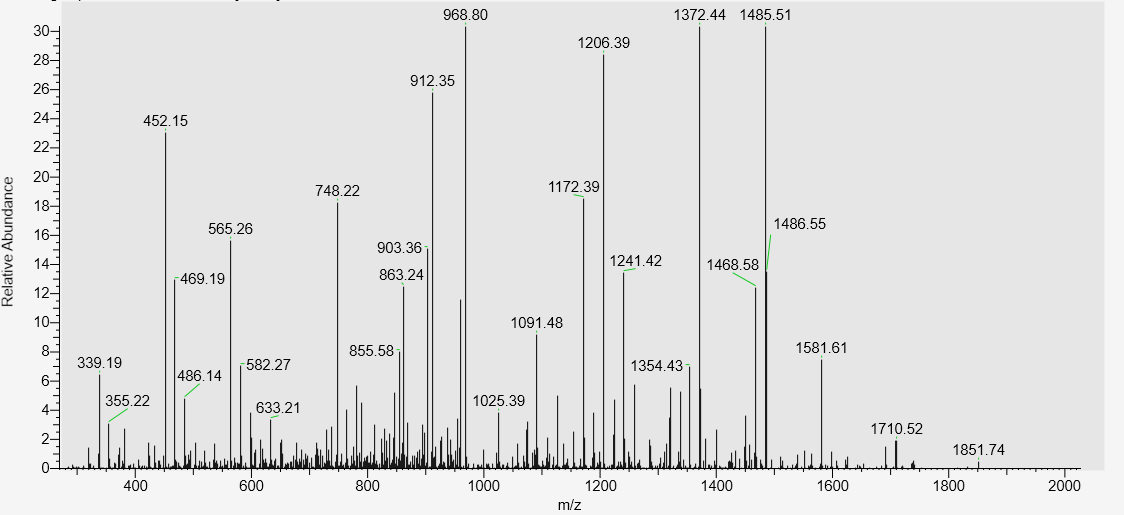


Figure S53. MS/MS spectrum of compound 239-2-ATF.


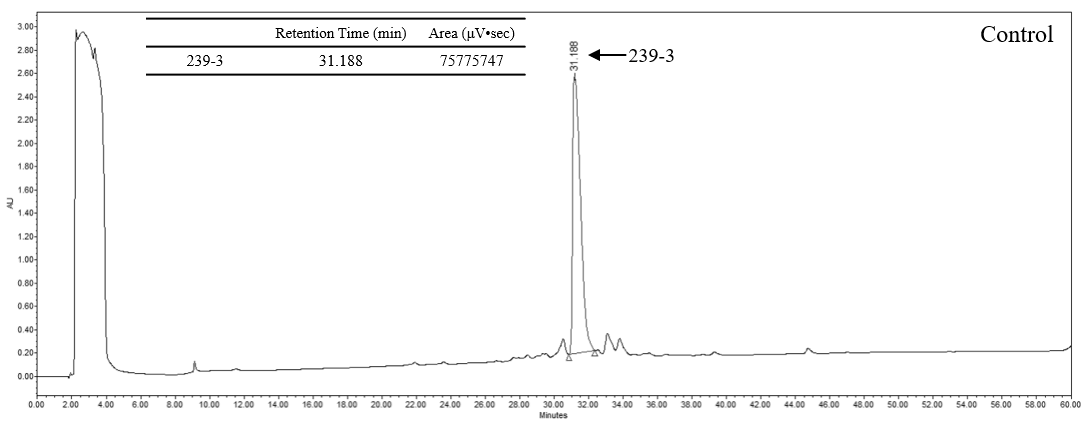

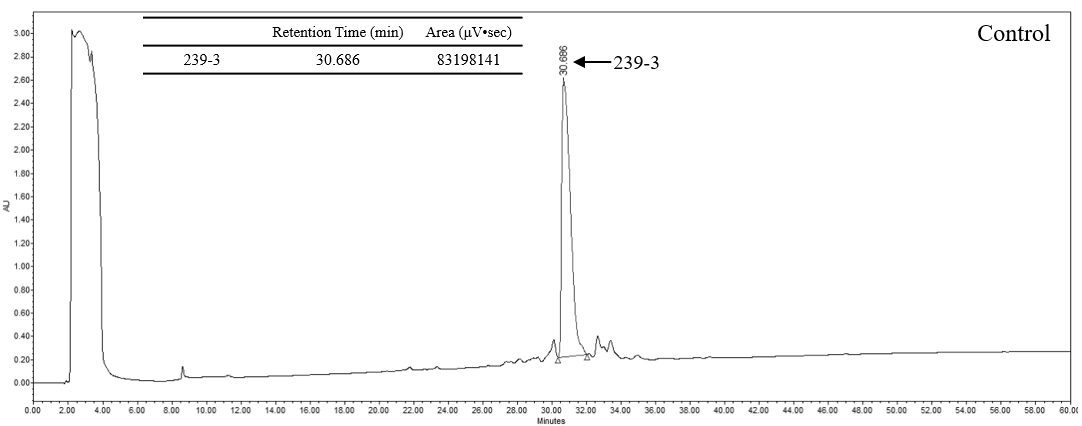


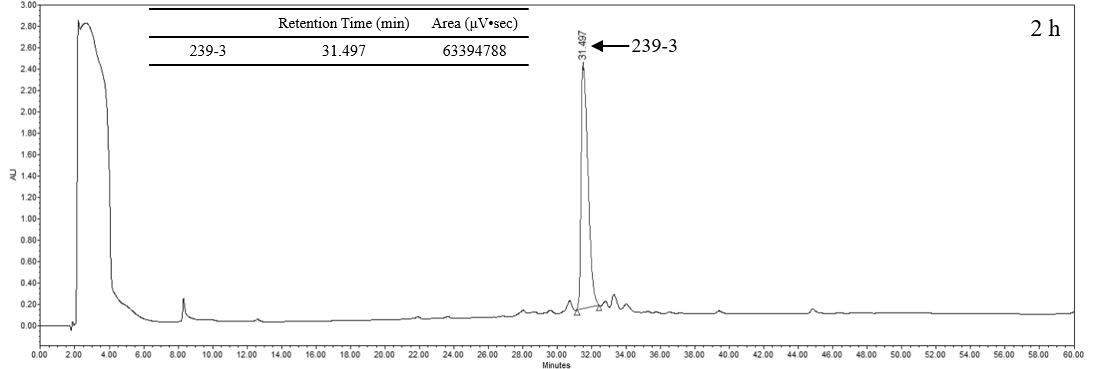

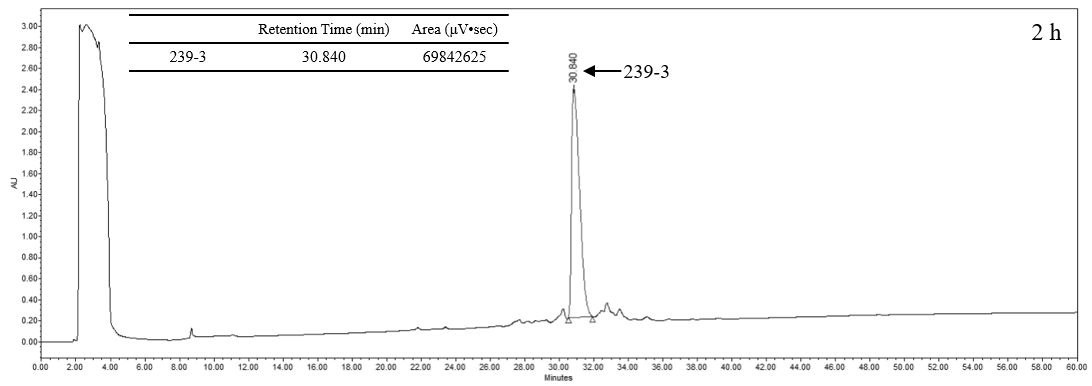


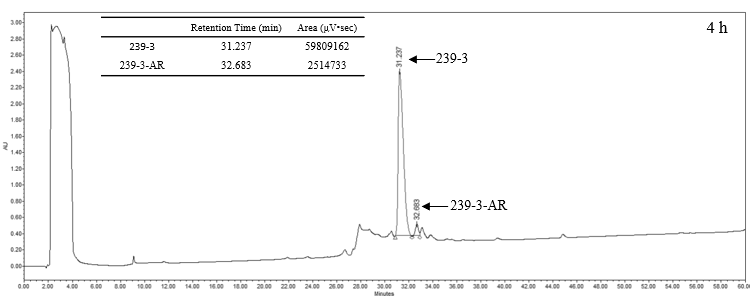

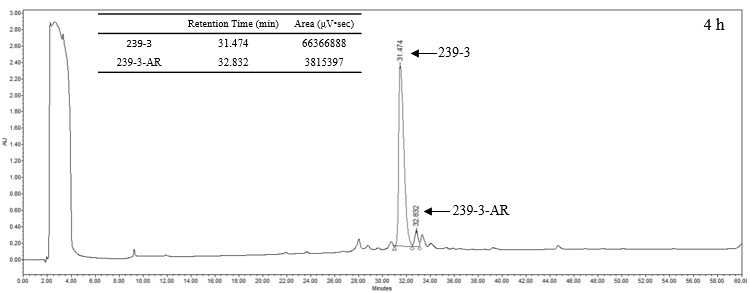


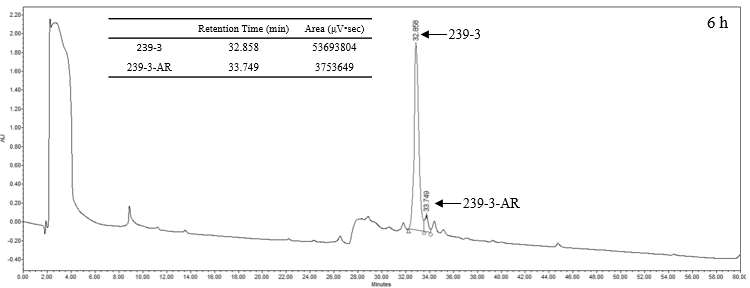

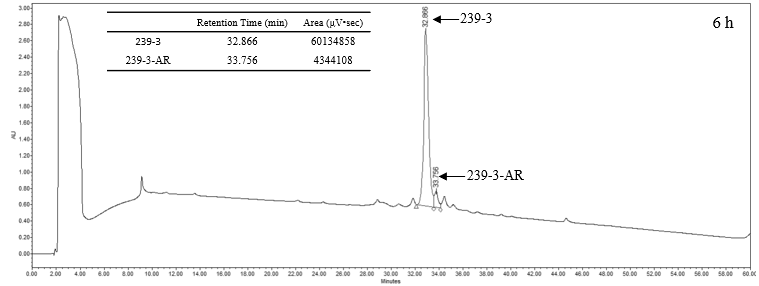


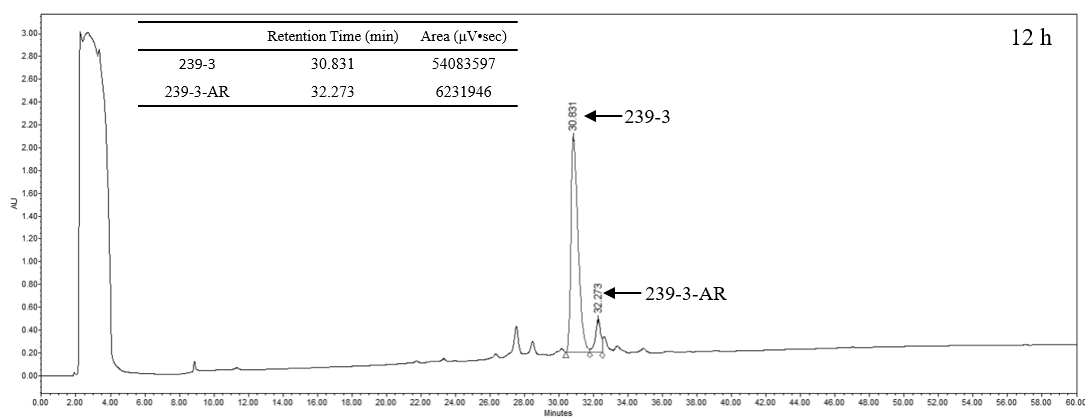

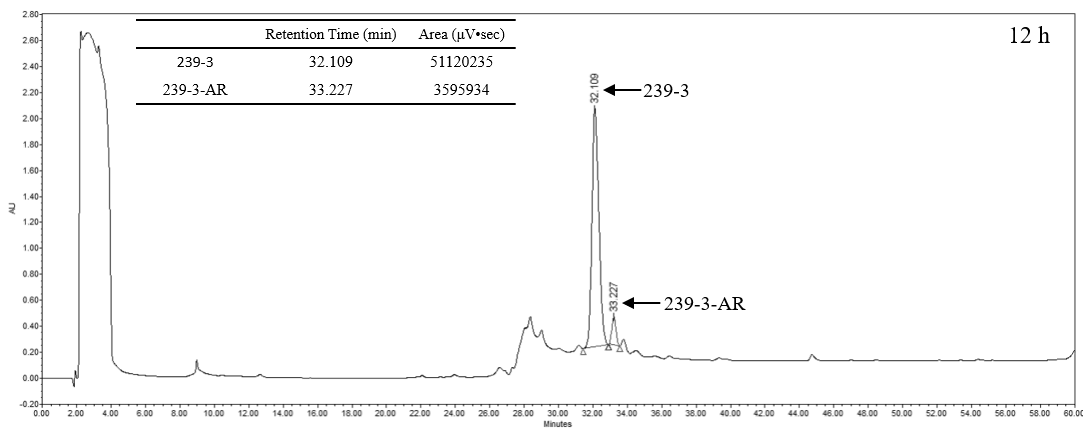


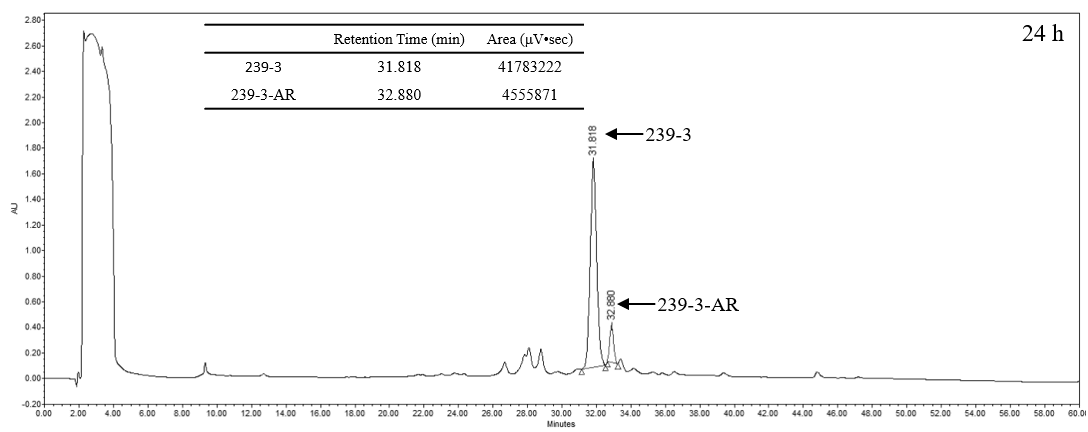

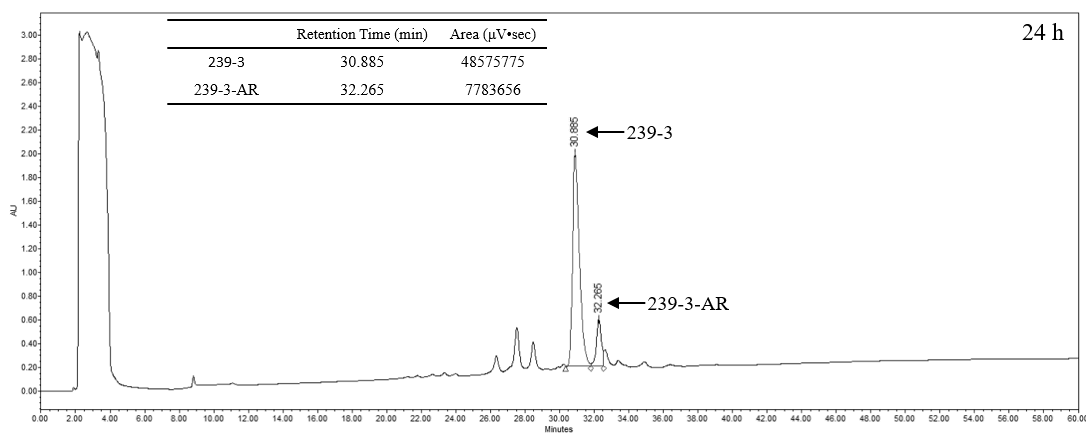


Figure S54. HPLC analysis of compound 239-3 incubated with 50% human serum for 0, 2, 4, 6, 12 and 24 h. Left and right panels represent two independent biological replicates (n = 2).


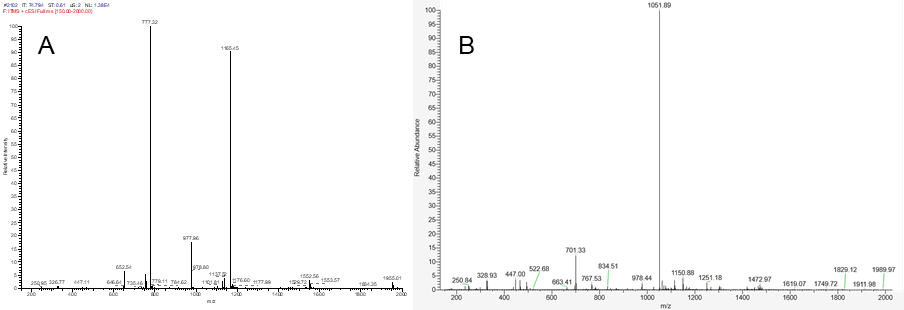


Figure S55. MS spectra of compound 239-3 peak (A) and compound 239-3-AR peak (B). Compound 239-3-AR calculated: [M+2H]^2+^: 1051.81, [M+3H]^3+^: 701.54; found: [M+2H]^2+^: 1051.89, [M+3H]^3+^: 701.33.

Table S7. MS/MS fragmentation sequencing data of compound 239-3-AR.

| 239-3-AR |  | | FFPKIFDDLE(PIP)SILPKIL-NH_2_ | | | | | |  |
| --- | --- | --- | --- | --- | --- | --- | --- | --- | --- |
|  | calculated | found | | |  | | calculated | | found |
| b_1_^+^ | 148.08 | | |  | | y_1_^+^ | | 1954.17 |  |
| b_2_^+^ | 295.14 | | |  | | y_2_^+^ | | 1807.10 |  |
| b_3_^+^ | 392.20 | | |  | | y_3_^+^ | | 1710.05 |  |
| b_4_^+^ | 520.29 | | |  | | y_4_^+^ | | 1581.95 | 1582.58 |
| b_5_^+^ | 633.38 | | |  | | y_5_^+^ | | 1468.87 | 1469.94 |
| b_6_^+^ | 780.44 | | |  | | y_6_^+^ | | 1321.80 | 1322.40 |
| b_7_^+^ | 895.47 | | |  | | y_7_^+^ | | 1206.77 | 1207.54 |
| b_8_^+^ | 1010.50 | | |  | | y_8_^+^ | | 1091.75 |  |
| b_9_^+^ | 1123.58 | | |  | | y_9_^+^ | | 978.66 |  |
| b_10_^+^ | 1319.70 | | |  | | y_10_^+^ | | 782.54 |  |
| b_11_^+^ | 1406.74 | | |  | | y_11_^+^ | | 695.51 |  |
| b_12_^+^ | 1519.82 | | | 1520.48 | | y_12_^+^ | | 582.43 |  |
| b_13_^+^ | 1632.90 | | | 1633.87 | | y_13_^+^ | | 469.34 |  |
| b_14_^+^ | 1729.96 | | |  | | y_14_^+^ | | 372.29 |  |
| b_15_^+^ | 1858.05 | | |  | | y_15_^+^ | | 244.19 |  |
| b_16_^+^ | 1971.14 | | |  | | y_16_^+^ | | 131.11 |  |
| b_17_^+^ | 2084.22 | | |  | | y_17_^+^ | | 17.03 |  |


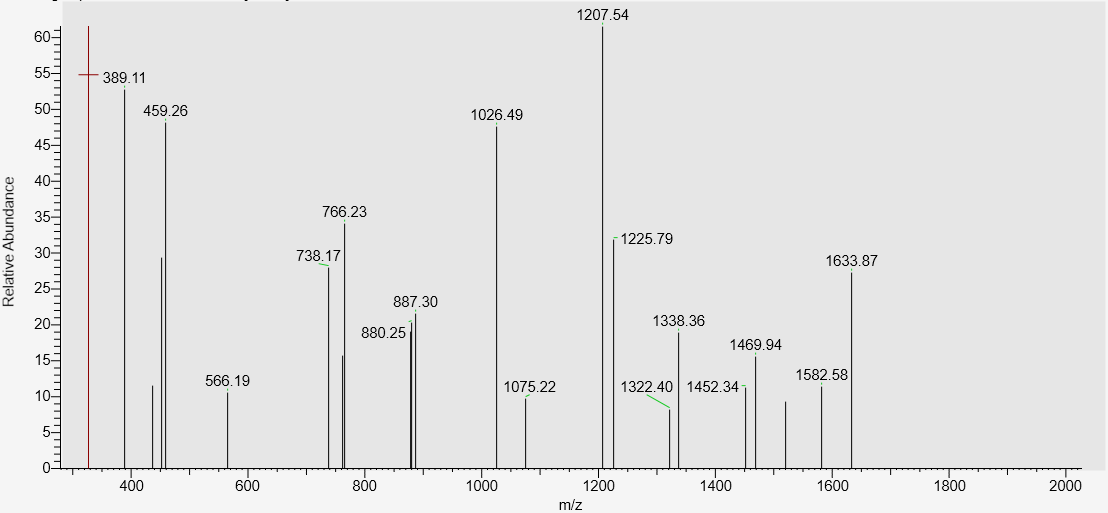


Figure S56. MS/MS spectrum of compound 239-3-AR.


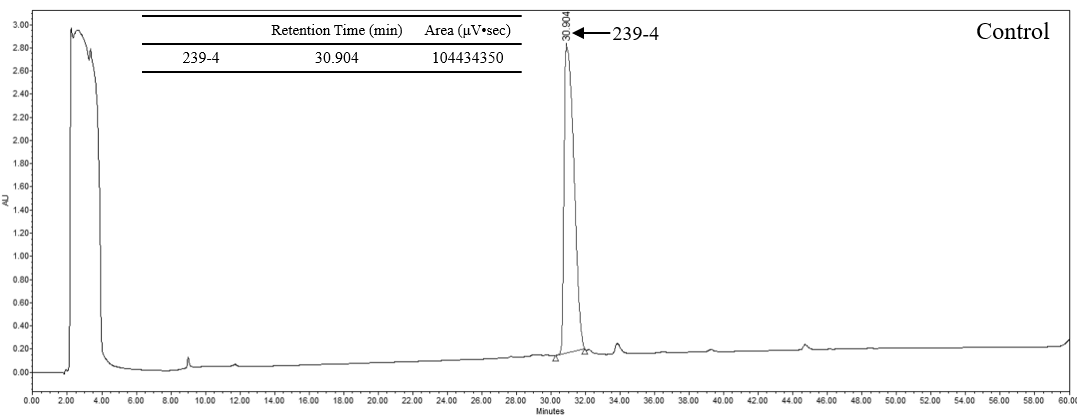

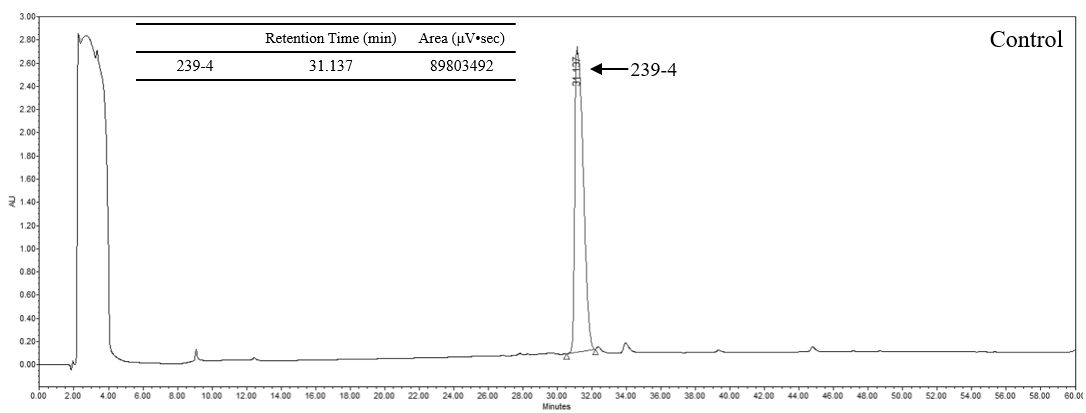


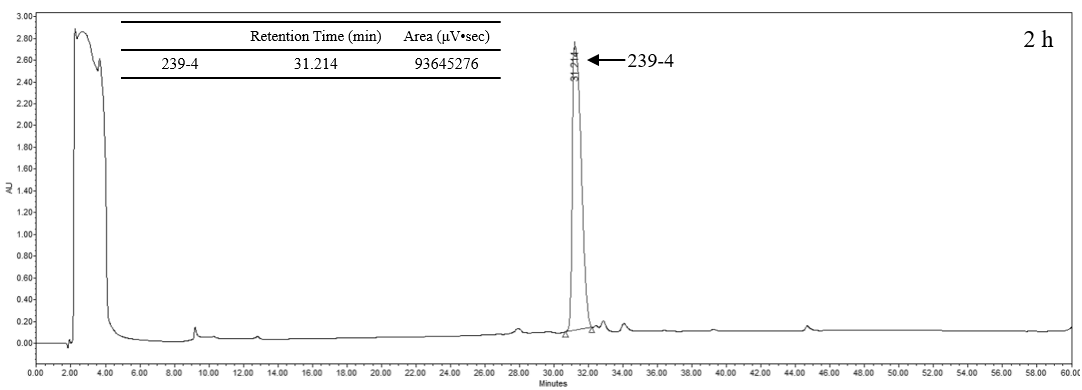

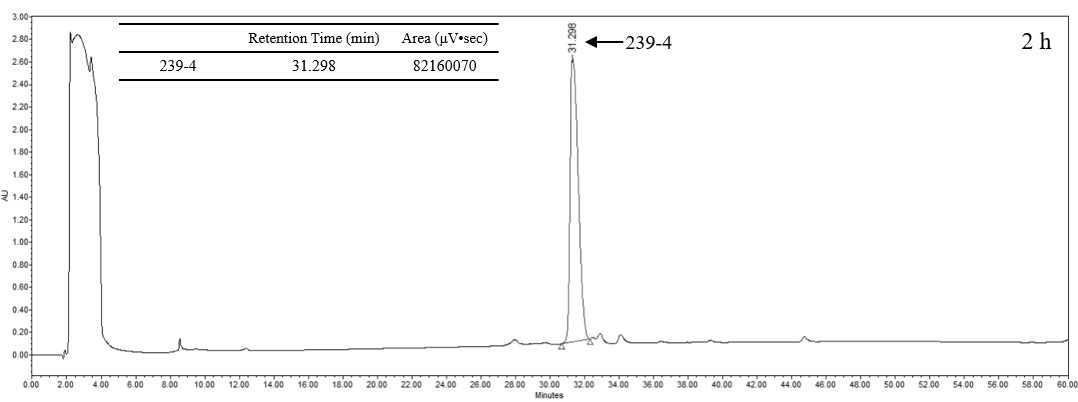


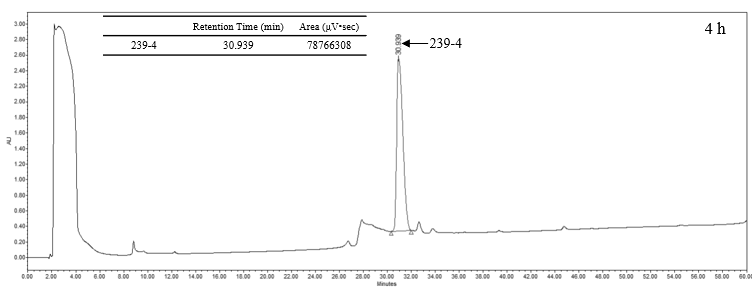

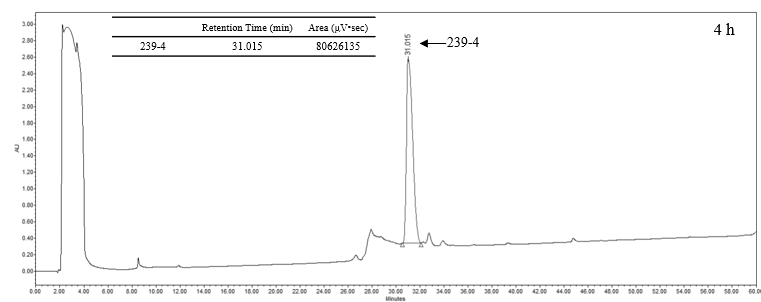

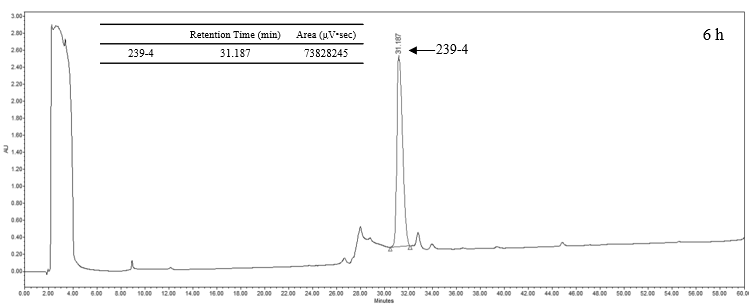

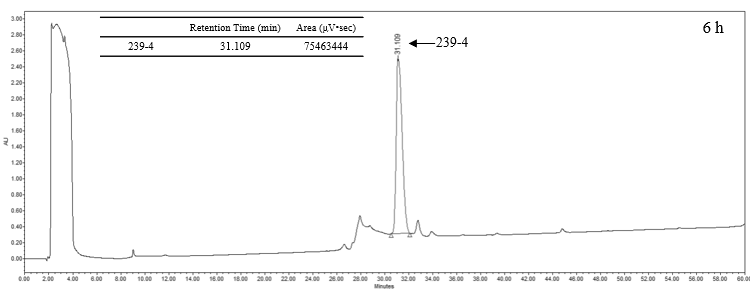


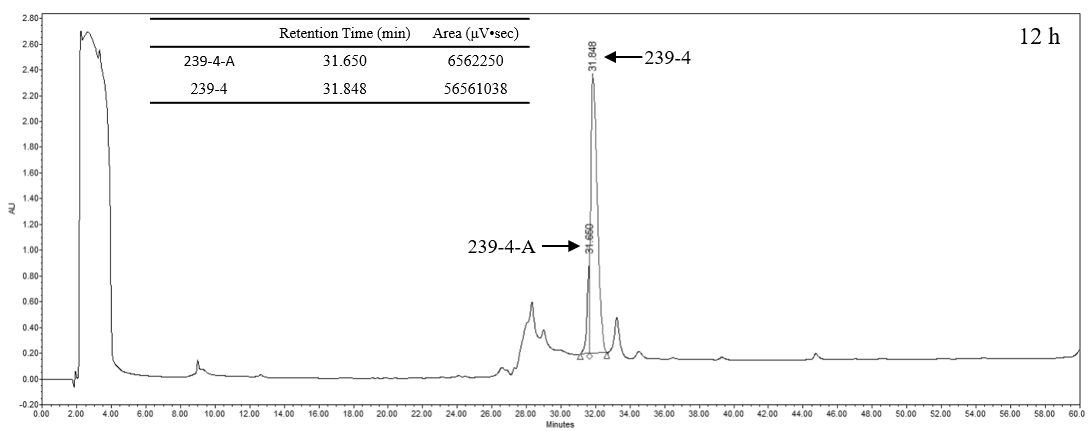

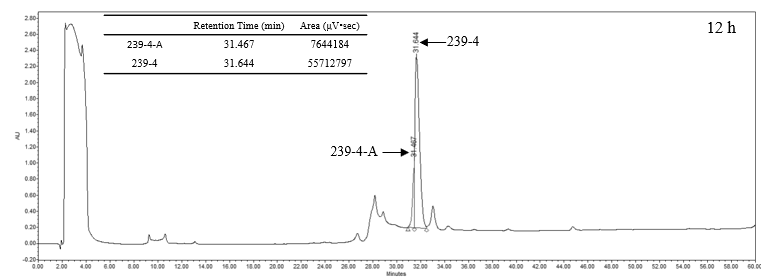


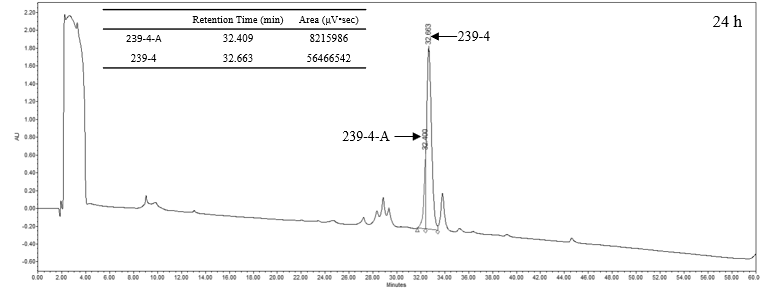

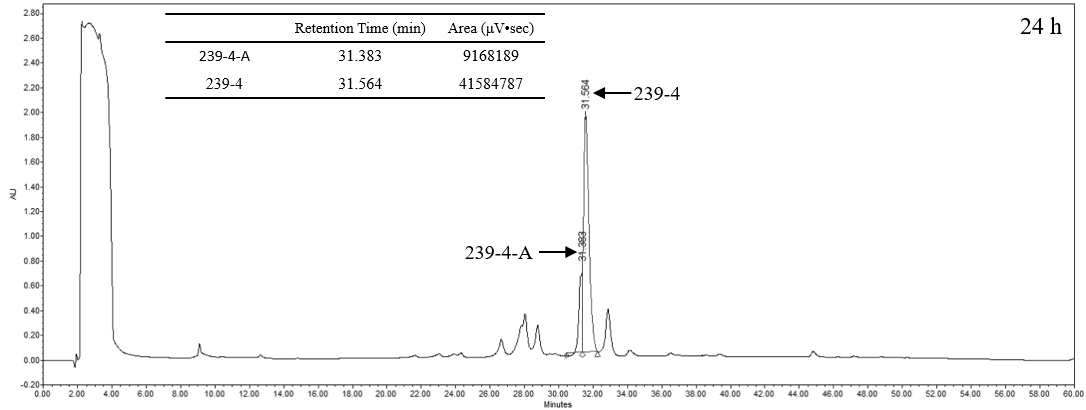


Figure S57. HPLC analysis of compound 239-4 incubated with 50% human serum for 0, 2, 4, 6, 12 and 24 h. Left and right panels represent two independent biological replicates (n = 2).


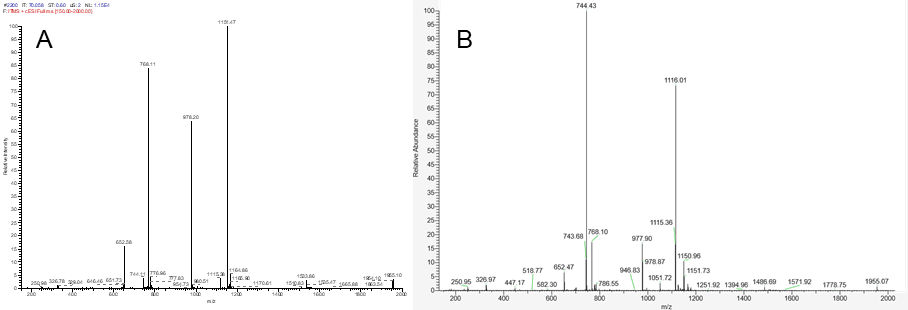


Figure S58. MS spectra of compound 239-4 peak (A) and compound 239-4-A peak (B). Compound 239-4-A calculated: [M+2H]^2+^: 1115.90, [M+3H]^3+^: 744.26; found: [M+2H]^2+^: 1116.01, [M+3H]^3+^: 744.43.

Table S8. MS/MS fragmentation sequencing data of compound 239-4-A.

| 239-4-A |  | | KFFPKIFDDLE(PIP)SILPKIL-NH_2_ | | | | | |  |
| --- | --- | --- | --- | --- | --- | --- | --- | --- | --- |
|  | calculated | found | | |  | | calculated | | found |
| b_1_^+^ | 130.11 | | |  | | y_1_^+^ | | 2101.24 |  |
| b_2_^+^ | 277.18 | | |  | | y_2_^+^ | | 1954.17 |  |
| b_3_^+^ | 424.45 | | | 424.49 | | y_3_^+^ | | 1807.10 | 1807.08 |
| b_4_^+^ | 521.30 | | | 521.27 | | y_4_^+^ | | 1710.05 | 1709.83 |
| b_5_^+^ | 649.40 | | | 649.38 | | y_5_^+^ | | 1581.95 | 1581.86 |
| b_6_^+^ | 762.48 | | | 762.42 | | y_6_^+^ | | 1468.87 | 1468.77 |
| b_7_^+^ | 909.55 | | | 909.55 | | y_7_^+^ | | 1321.80 | 1321.65 |
| b_8_^+^ | 1024.57 | | | 1024.51 | | y_8_^+^ | | 1206.77 | 1206.58 |
| b_9_^+^ | 1139.60 | | | 1139.46 | | y_9_^+^ | | 1091.75 | 1091.66 |
| b_10_^+^ | 1252.69 | | | 1252.62 | | y_10_^+^ | | 978.66 | 978.23 |
| b_11_^+^ | 1448.81 | | | 1448.75 | | y_11_^+^ | | 782.54 | 782.69 |
| b_12_^+^ | 1535.84 | | | 1535.79 | | y_12_^+^ | | 695.51 |  |
| b_13_^+^ | 1648.92 | | | 1648.82 | | y_13_^+^ | | 582.43 | 582.26 |
| b_14_^+^ | 1762.01 | | | 1761.96 | | y_14_^+^ | | 469.34 | 469.20 |
| b_15_^+^ | 1859.06 | | |  | | y_15_^+^ | | 372.29 |  |
| b_16_^+^ | 1987.15 | | |  | | y_16_^+^ | | 244.19 |  |
| b_17_^+^ | 2100.24 | | |  | | y_17_^+^ | | 131.11 |  |
| b_18_^+^ | 2213.32 | | |  | | y_18_^+^ | | 17.03 |  |


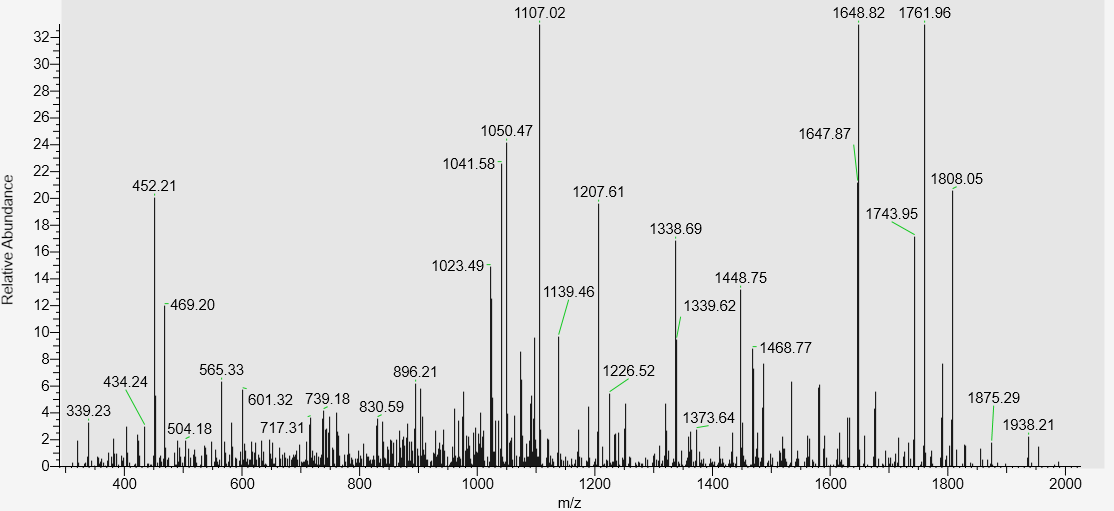


Figure S59. MS/MS spectrum of compound 239-4-A.


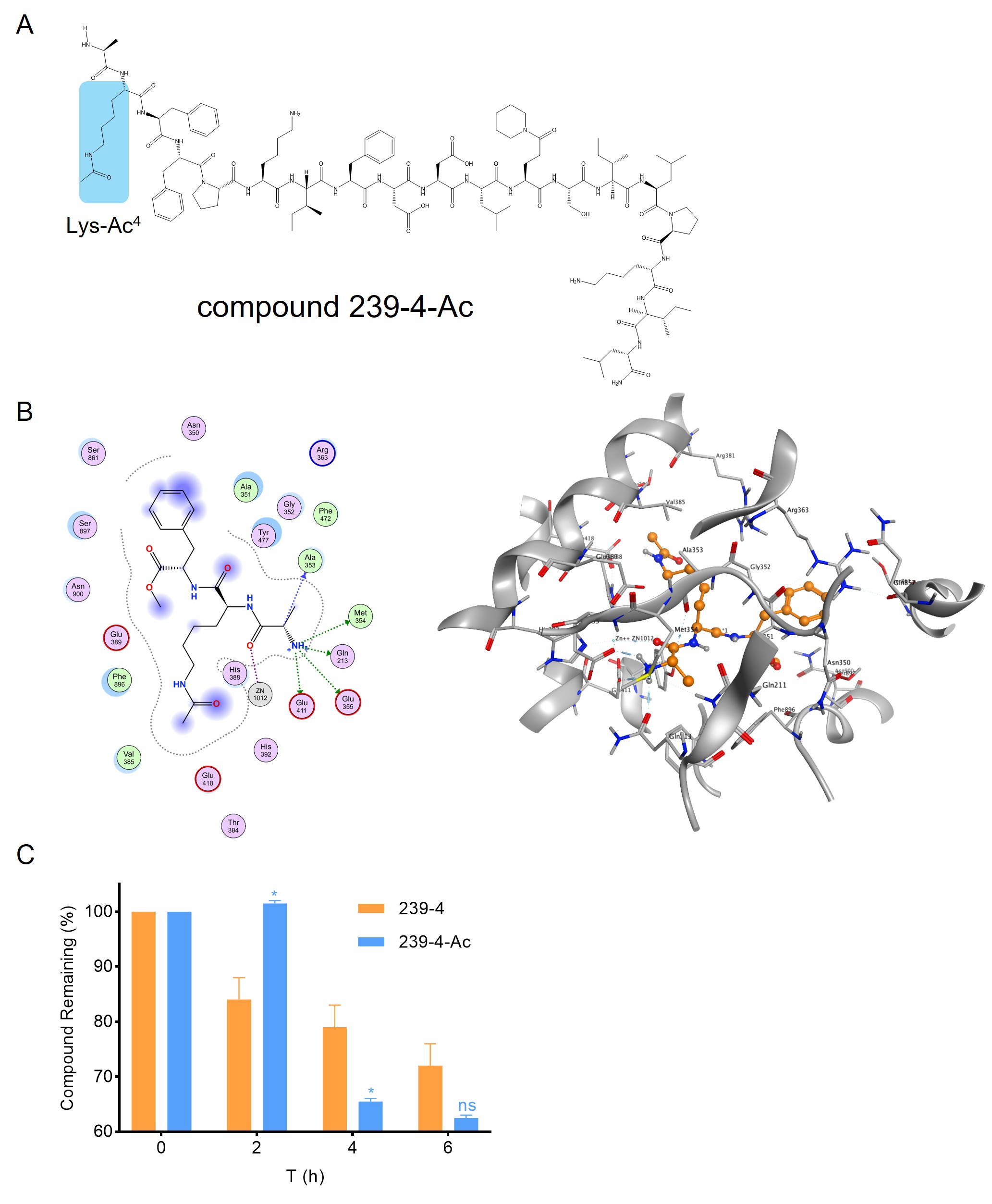


Figure S60. (A) Chemical structure of compound 239-4-Ac. (B) Predicted docking 2D interaction patterns (left) and 3D cartoon interaction patterns (right) between APN and the tripeptide AK(Ac)F. In 3D cartoon interaction, receptors are grey, and ligands are orange. Blue dashed lines represent hydrogen bonds. For interpretation of the legend in the 2D interaction patterns, refer to Figure S38 A. (C) Compound remaining (%) of compounds 239-4 and 239-4-Ac after incubation with human serum for up to 6 h. Data are presented as mean ± SEM, n=2. Unpaired two-tailed t-test was used to compare compounds 239-4-Ac with 239-4 at each time point. ns for P > 0.05 and * for P < 0.05.

Figure S61. HPLC analysis of compound 239-4-Ac incubated with 50% human serum for 0, 2, 4 and 6 h. Left and right panels represent two independent biological replicates (n = 2).

Figure S62. MS spectra of compound 239-4-Ac peak (A), compound 239-4-Ac-1 peak (B) and compound 239-4-Ac-2 peak (C). Compound 239-4-Ac-1 calculated: [M+2H]^2+^: 978.22; found: [M+2H]^2+^: 978.17. Compound 239-4-Ac-2 calculated: [M+2H]^2+^: 1051.81, [M+3H]^3+^: 701.54; found: [M+2H]^2+^: 1051.40, [M+3H]^3+^: 701.48.

Table S9. MS/MS fragmentation sequencing data of compound 239-4-Ac-1.

| 239-4-Ac-1 | FPKIFDDLE(PIP)SILPKIL-NH_2_ | | | | | | | |
| --- | --- | --- | --- | --- | --- | --- | --- | --- |
|  | calculated | found | |  | | calculated | | found |
| b_1_^+^ | 148.08 | |  | | y_1_^+^ | | 1807.10 |  |
| b_2_^+^ | 245.13 | |  | | y_2_^+^ | | 1710.05 |  |
| b_3_^+^ | 373.22 | | 373.38 | | y_3_^+^ | | 1581.95 | 1581.90 |
| b_4_^+^ | 486.31 | | 486.24 | | y_4_^+^ | | 1468.87 | 1468.69 |
| b_5_^+^ | 633.37 | |  | | y_5_^+^ | | 1321.80 |  |
| b_6_^+^ | 748.40 | | 748.19 | | y_6_^+^ | | 1206.77 | 1206.52 |
| b_7_^+^ | 863.43 | | 863.72 | | y_7_^+^ | | 1091.75 | 1091.51 |
| b_8_^+^ | 976.51 | |  | | y_8_^+^ | | 978.66 | 977.99 |
| b_9_^+^ | 1172.63 | | 1172.62 | | y_9_^+^ | | 782.54 | 782.54 |
| b_10_^+^ | 1259.67 | | 1259.47 | | y_10_^+^ | | 695.51 |  |
| b_11_^+^ | 1372.75 | | 1372.54 | | y_11_^+^ | | 582.43 | 582.35 |
| b_12_^+^ | 1485.83 | | 1485.67 | | y_12_^+^ | | 469.34 | 469.27 |
| b_13_^+^ | 1582.89 | |  | | y_13_^+^ | | 372.29 |  |
| b_14_^+^ | 1710.98 | |  | | y_14_^+^ | | 244.19 |  |
| b_15_^+^ | 1824.07 | |  | | y_15_^+^ | | 131.11 |  |
| b_16_^+^ | 1937.15 | |  | | y_16_^+^ | | 17.03 |  |

Figure S63. MS/MS spectrum of compound 239-4-Ac-1.

Table S10. MS/MS fragmentation sequencing data of compound 239-4-Ac-2.

| 239-4-Ac-2 |  | | FFPKIFDDLE(PIP)SILPKIL-NH_2_ | | | | | |  |
| --- | --- | --- | --- | --- | --- | --- | --- | --- | --- |
|  | calculated | found | | |  | | calculated | | found |
| b_1_^+^ | 148.08 | | |  | | y_1_^+^ | | 1954.17 |  |
| b_2_^+^ | 295.14 | | |  | | y_2_^+^ | | 1807.10 | 1807.99 |
| b_3_^+^ | 392.20 | | | 391.79 | | y_3_^+^ | | 1710.05 | 1710.95 |
| b_4_^+^ | 520.29 | | | 520.23 | | y_4_^+^ | | 1581.95 | 1581.86 |
| b_5_^+^ | 633.38 | | | 633.19 | | y_5_^+^ | | 1468.87 | 1468.71 |
| b_6_^+^ | 780.44 | | | 780.34 | | y_6_^+^ | | 1321.80 | 1321.61 |
| b_7_^+^ | 895.47 | | | 895.46 | | y_7_^+^ | | 1206.77 | 1206.53 |
| b_8_^+^ | 1010.50 | | | 1010.37 | | y_8_^+^ | | 1091.75 | 1091.68 |
| b_9_^+^ | 1123.58 | | | 1123.27 | | y_9_^+^ | | 978.66 | 978.34 |
| b_10_^+^ | 1319.70 | | | 1319.56 | | y_10_^+^ | | 782.54 | 782.39 |
| b_11_^+^ | 1406.74 | | | 1406.50 | | y_11_^+^ | | 695.51 |  |
| b_12_^+^ | 1519.82 | | | 1519.48 | | y_12_^+^ | | 582.43 | 582.28 |
| b_13_^+^ | 1632.90 | | | 1632.72 | | y_13_^+^ | | 469.34 | 469.17 |
| b_14_^+^ | 1729.96 | | |  | | y_14_^+^ | | 372.29 |  |
| b_15_^+^ | 1858.05 | | |  | | y_15_^+^ | | 244.19 |  |
| b_16_^+^ | 1971.14 | | | 1971.81 | | y_16_^+^ | | 131.11 |  |
| b_17_^+^ | 2084.22 | | |  | | y_17_^+^ | | 17.03 |  |

Figure S64. MS/MS spectrum of compound 239-4-Ac-2.

Figure S65. (A) LDH release (%) of different cell lines after 24 h-treatment with conjugate 270. (B) Pictures of each group were recorded everyday across the *in vivo* safety evaluation. All the black waxworms were dead. From the third day onward, partial dark brown pigmentation was observed in some waxworms. This represents a normal cocooning process rather than a sign of toxicity. (C) Annexin V/7-AAD-based flow cytometry analysis of H460 cells treated with conjugate 270 at IC_50_ value. (D) Caspase-3/7&7-AAD-based flow cytometry analysis of H460 cells treated with conjugate 270. (E) Membrane potential of H460 cells treated with conjugate 270 at IC_50_ value, evaluated by fluorescence intensity. (F) LDH release (%) of H460 cells after 4 h-treatment with conjugate 270 or etoposide at each IC_50_ value. (G) Fluorescence images of H460 cells treated with conjugate 270 at IC_50_ value for 2 h. The green channel was SYTOX Green and it was merged with bright fields. Scale bar: 250 μm. Data are presented as mean ± SEM, n=3. One-way ANOVA (A, C and D) was used to compare each treatment group with the control group in (C) and (D), or with the H460 dosing group at the same potency level in (A). Unpaired two-tailed t-test was used to compare the treatment group with the control group at 40 min in (E) or the doing group with the etoposide group after 4 h-incubation in (F). ** for P < 0.01 and **** for P < 0.0001.

Figure S66. HPLC analysis of conjugate 270 incubated with 50% human serum for 0, 2, 4, 6, 12 and 24 h. Left and right panels represent two independent biological replicates (n = 2).

Figure S67. MS spectrum of conjugate 270 peak.

Figure S68. Original images of killing rate evaluation. H460 cells were treated with four peptides at IC_50_ (A) or IC_95_ (B) concentrations for 24 h, stained by calcein-AM and PI, and finally recorded with fluorescence microscopy. The control group (C) was treated with 0.5% DMSO in SFM while the etoposide group (D) was treated with etoposide at IC_50_ concentration as a comparison group. Scale bar: 250 μm.

Table S11. IC_50_ values of four chemotherapeutics towards H460 and HEK-293 cell lines.

| **​** | IC_50_ Value (µM)​ | | | |
| --- | --- | --- | --- | --- |
|  | Cisplatin | 5-Fluorouracil | Paclitaxel | Etoposide |
| H460 | 203.45±26.85 | 432.05±61.25 | 220.45±64.95 | 168.95±29.15 |
| HEK-293 | 162.5±18.8 | 430.4±40.2 | <7 | 115.45±14.65 |

Figure S69. Antiproliferative effects of four chemotherapeutics against four H460 chemo-resistant cell lines (A). Antiproliferative effects of four peptides against cisplatin-resistant (B), 5-fluorouracil-resistant (C), paclitaxel-resistant (D) and etoposide-resistant (E) H460 cell lines detected by MTT assays. LDH release (%) of cisplatin-resistant (F), 5-fluorouracil-resistant (G) and paclitaxel-resistant (H) and etoposide-resistant (I) H460 cell lines after incubation with four or three peptides for 24 h.
